# Harnessing tethered nitreniums for diastereoselective amino-sulfonoxylation of alkenes

**DOI:** 10.3762/bjoc.21.78

**Published:** 2025-05-19

**Authors:** Shyam Sathyamoorthi, Appasaheb K Nirpal, Dnyaneshwar A Gorve, Steven P Kelley

**Affiliations:** 1 Department of Medicinal Chemistry, University of Kansas, Lawrence, Kansas 66047, United States; 2 Department of Chemistry, University of Missouri—Columbia, Columbia, Missouri 65211, United States

**Keywords:** alkene, amino-sulfonoxylation, metal-free, tethered nitrenium

## Abstract

We present the first examples of alkene amino-sulfonoxylation reactions that leverage the unique reactivity of carbamate tethered *N*-alkoxy nitrenium ions. In almost all cases examined, the reactions deliver product with exquisite regioselectivity and diastereoselectivity. The protocols followed are operationally very simple and only use commercial I(III) reagents and sulfonic acids, amounting to a metal-free protocol for alkene amino-oxygenation. No special precautions need be taken to exclude air or ambient moisture, and the products are amenable to further transformations.

## Introduction

**Scheme 1:**
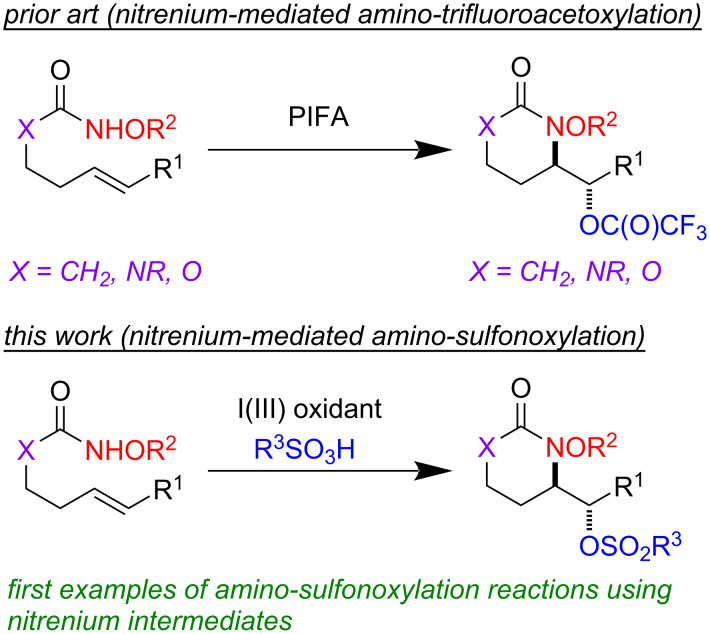
Existing reports of intramolecular alkene functionalization reactions with nitreniums have focused on amino-trifluoroacetoxylations. We show the first examples of amino-sulfonoxylations using tethered nitreniums.

Our laboratory has a programmatic focus on the development of metal-free oxidation reactions that avoid the use of toxic reagents such as osmium and chromium [1–3]. In line with this agenda, we recently explored a mild amino-trifluoroacetoxylation of alkenes [4]. In this protocol, an unusual *N*-alkoxy carbamate tether served as a precursor to a transient nitrenium ion [5–24], which subsequently attacked a pendant alkene to form an aziridinium intermediate. This aziridinium ring was opened in a diastereoselective (S_N_2 type) and *exo*-selective manner by a trifluoroacetate anion. The trifluoroacetate anion was conveniently derived from (bis(trifluoroacetoxy)iodo)benzene (PIFA), which was used as the stoichiometric oxidant in the reaction. Overall, this amounted to a highly regioselective, diastereoselective, and metal-free protocol for alkene amino-hydroxylation, which compared favorably to prior art in this area [25–32]. Naturally, we wondered if other *O*-nucleophiles were competent in the ring-opening of the aziridinium intermediate. Indeed, almost all examples of alkene amino-hydroxylation reactions mediated by *N*-alkoxy nitreniums deliver amino-trifluoroacetate products (Scheme 1) [6]. Here, we describe the first examples of amino-sulfonoxylation reactions of alkenes, which make use of carbamate tethered *N*-alkoxy nitrenium ions.

## Results and Discussion

Reaction exploration began with (*E*)*-*hex-3-en-1-yl methoxycarbamate, prepared in good yield from widely available *trans*-3-hexen-1-ol (Table 1) using a two-step protocol (1. 1,1’-carbonyldiimidazole (CDI), CH_2_Cl_2_; 2. MeONH_2_·HCl, pyridine) [33]. Based on insights from our work with I(III)-promoted alkene disulfonoxylation [2] and alkene amino-trifluoroacetoxylation [4], we hypothesized that treatment of (*E*)*-*hex-3-en-1-yl methoxycarbamate with a mixture of an I(III) oxidant and a sulfonic acid would lead to the formation of amino-sulfonoxylated product. We were thus very pleased to observe 59% of desired product **B** using a combination of 1 equivalent of commercially available 1-acetoxy-1,2-benziodoxol-3-(1*H*)-one (CAS [1829-26-1]) and 1 equivalent of methanesulfonic acid (MsOH) (Table 1, entry 1). Increasing the equivalents of both reagents to 1.5 completely consumed starting material and delivered product **B** in an excellent yield of 73% (Table 1, entry 2). A similar yield was achieved by using 1.5 equivalents of commercially available PhI(OH)(OMs) (CAS [105551-42-6]) (Table 1, entry 3). With these conditions, adding exogenous MsOH was not necessary. Reasonable amounts of product **B** could also be obtained with 1 equivalent of commercial iodomesitylene diacetate (CAS [33035-41-5]) and 1 equivalent of MsOH (Table 1, entry 4). Here, it was necessary to maintain a temperature of 0 °C, as vigorous bubbling and rapid decomposition occurred when the reaction was initiated at room temperature. In all cases (Table 1), only a single regioisomer and diastereomer of **B** was formed (within the limits of ^1^H NMR detection), attesting to very selective reactivity. Here and in related projects, we found that many I(III) sources could generate a nitrenium ion, including iodosobenzene (PhIO) and iodomesitylene diacetate. However, unless there was a weakly basic counter anion with excellent displaceability, such as trifluoroacetate or sulfonate, the nitrenium refused to engage with the pendant alkene, and complex mixtures, presumably containing *N*-*N* dimeric products of starting material, were observed.

**Table 1:** Optimization results.

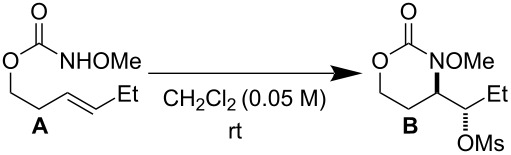			

	I(III) Oxidant	MsOH	Yield

1	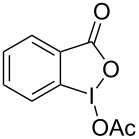 (1 equiv)	1 equiv	59% **B** 25% **A**
2	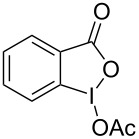 (1.5 equiv)	1.5 equiv	73% **B** 0% **A**
3	PhI(OH)(OMs) (1.5 equiv)	none	70% **B** 0% **A**
4^a^	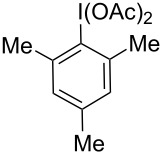 (1 equiv)	1 equiv	58% **B** 0% **A**

^a^Temperature maintained at 0 °C to prevent rapid decomposition.

During reaction optimization, we had only examined reactions with an *N*-methoxy carbamate substrate. We next wished to study the compatibility of other *N*-alkoxy carbamate tethers with our newly developed reaction (Table 2). Our survey revealed that *N*-ethoxy, *N*-*n*-butoxy, and *N*-isobutoxy carbamate tethers were all equally competent in delivering product (Table 2, entries 1 and 3–5). With our standard conditions of stirring substrate with 1.5 equivalents of PhI(OH)(OTs) in CH_2_Cl_2_ at room temperature, the yield of product markedly suffered with *N*-isopropoxy carbamate substrate **3** (Table 2, entry 2). Here, we hypothesize that the increased steric bulk of the isopropyl group unfavorably affected the reaction outcome. However, we found that when **3** was subjected to the more active combination of iodomesitylene diacetate and *p-*TsOH·H_2_O at 0 °C in CH_2_Cl_2_, the desired product **4** formed in a much-improved yield of 57%. Not all tethers were compatible with our optimized conditions (Table 2, poor performers). In many cases, complex mixtures of recovered starting material and multiple products were observed. Carbamate tethers with *N*-alkoxy substituents were essential for clean reactions; indeed, extensive decomposition was observed with *N*-ethyl carbamate substrate **15** and *N*-hydroxy carbamate substrate **16**.

**Table 2:** Structure–reactivity relationship with nitrenium tethers.

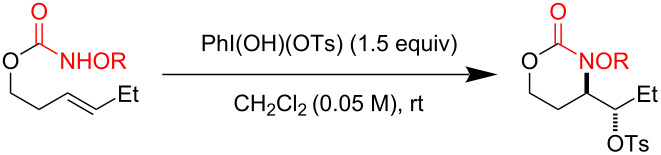				

Entry	Substrate	Product	Substrate/product number	Isolated yield

1	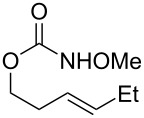	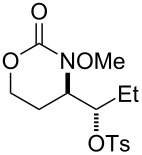	**1**, **2**	73%
2	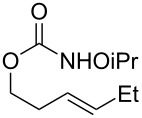	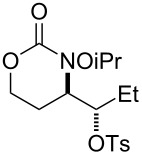	**3**, **4**	34% 57%^a^
3	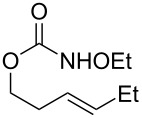	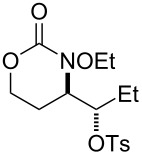	**5**, **6**	79%
4	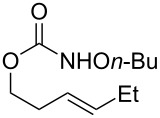	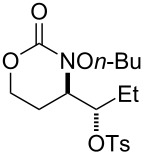	**7**, **8**	74%
5	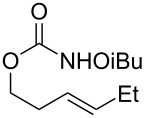	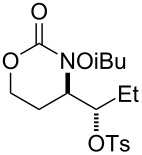	**9**, **10**	84%

^a^Reaction conditions: iodomesitylene diacetate (1.5 equiv), *p-*TsOH·H_2_O (1.5 equiv), CH_2_Cl_2_, 0 °C, 2 h.
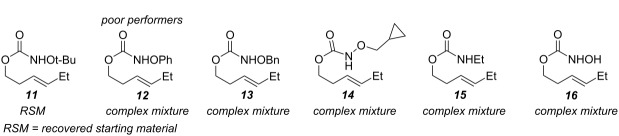

As we had successfully installed tosyloxy and mesyloxy groups, we wondered if other sulfonic acids would be compatible with our reaction conditions (Table 3). We were pleased to find that the amino-sulfonoxylation reaction proceeded with a diverse array of sulfonic acids, both alkyl and aryl. Koser’s reagents [PhI(OH)(OTs) and PhI(OH)(OMs)] are commercially available and contain a sulfonate covalently attached to I(III). Simply stirring these reagents with carbamate substrate cleanly delivered tosyloxylated and mesyloxylated products. For many other sulfonoxylated products, we found that stirring substrate with a combination of 1-acetoxy-1,2-benziodoxol-3-(1*H*)-one and the appropriate sulfonic acid worked best. Here, we hypothesize that rapid exchange of the acetate for the sulfonate occurred at the I(III) nucleus, and this unstable sulfonoxy reagent served to initiate nitrenium formation and alkene oxidation. Not all sulfonic acids, however, were compatible with these conditions and were recalcitrant for steric or electronic reasons. For these acids, replacing 1-acetoxy-1,2-benziodoxol-3-(1*H*)-one with the more reactive iodomesitylene diacetate was necessary for product formation (Table 3, products **20** and **25–28**). X-ray crystallographic analysis of **20** (CCDC 2391529) allowed us to unambiguously determine its identity and relative stereochemistry, and we have assigned other products by analogy.

**Table 3:** Examination of sulfonic acid scope.

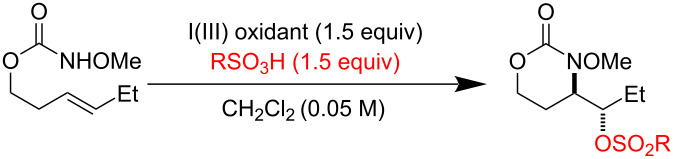			

Entry	Product	Product number	Isolated yield

1	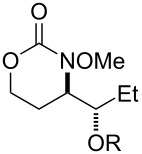	R = PhSO_2_: **17**^a^ R = *p*-BrC_6_H_4_SO_2_: **18**^a^ R = *p*-FC_6_H_4_SO_2_: **19**^a^ R = *p*-NO_2_C_6_H_4_SO_2_: **20** (X-ray)^b^ R = *p*-ClC_6_H_4_SO_2_: **21**^a^ R = *p*-OMeC_6_H_4_SO_2_: **22**^a^ R = MeSO_2_: **23**^c^ R = EtSO_2_: **24**^a^	50% 62% 61% 66% 53% 60% 70% 60%
2	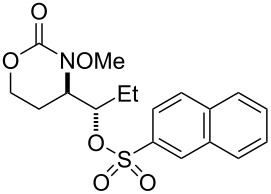	**25** ^b^	54%
3	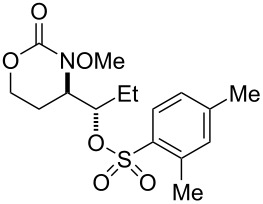	**26** ^b^	63%
4	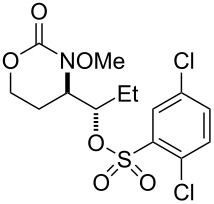	**27** ^b^	62%
5	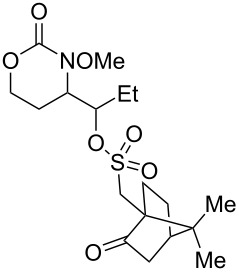	**28** ^b^	70% dr ≈ 1:1

^a^1-Acetoxy-1,2-benziodoxol-3-(1*H*)-one (1.5 equiv), RSO_3_H (1.5 equiv), CH_2_Cl_2_, rt; ^b^iodomesitylene diacetate (1.5 equiv), RSO_3_H (1.5 equiv), CH_2_Cl_2_, 0 °C to rt; ^c^PhI(OH)(OMs) (1.5 equiv), CH_2_Cl_2_, rt (note: no MsOH added).

A variety of carbamate substrates were compatible with our optimized protocols (Table 4). Earlier in the project, we had focused exclusively on substrates derived from *trans*-homo-allylic alcohols. We were thus pleased to find that a substrate synthesized from *cis*-3-hexen-1-ol was equally compatible and gave product in an excellent yield and diastereoselectivity (Table 4, entry 1). A urea substrate also furnished the expected product in a good yield and with high diastereoselectivity (Table 4, entry 3). With tri-substituted allylic carbamate **35**, some amount of expected mesyloxylated product **36** did form, but there was also an isolable amount of a terminal alkene side product, presumably arising from mesylate elimination (Table 4, entry 4). In general, the functional group tolerance of the reaction was good, and aryl halogens, aryl CF_3_ moieties, and benzyl ethers were fully compatible (Table 4, entries 7 and 8). Where applicable, stereoarrays could be synthesized in one pot with good yields and excellent diastereoselectivities (Table 4, entries 5 and 9).

**Table 4:** Substrate scope exploration with an emphasis on stereocontrol and functional group compatibility.

Entry	Substrate	Product	Substrate/product number	Isolated yield

1	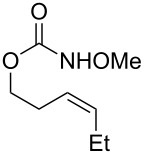	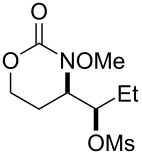	**29**, **30**	72%^a^
2	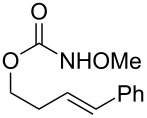	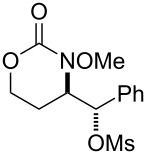	**31**, **32**	60%^b^
3	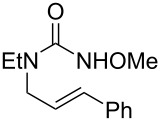	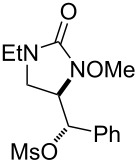	**33**, **34**	53%^a^
4	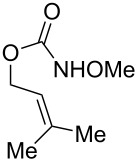	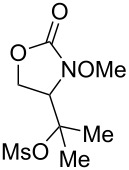	**35**, **36**	37%^a,c^
5	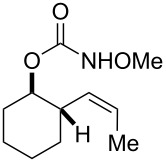	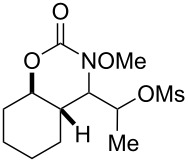	**37**, **38**	92%^a^ dr > 20:1
6	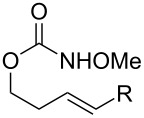	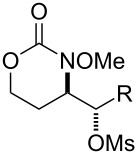	R = iPr: **39**, **40** R = CH_2_-cyhex: **41**, **42**	67%^a^ 80%^a^
7	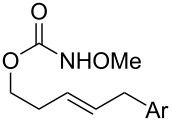	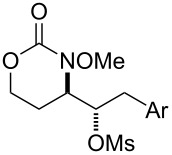	Ar = Ph: **43**, **44** Ar = *m*-CF_3_C_6_H_4_: **45**, **46** Ar = *p*-ClC_6_H_4_: **47**, **48** Ar = *p*-BrC_6_H_4_: **49**, **50** Ar = *p*-FC_6_H_4_: **51**, **52**	68%^a^ 56%^a^ 70%^a^ 69%^a^ 71%^a^
8	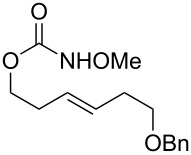	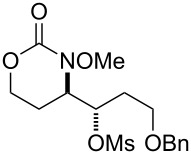	**53**, **54**	68%^a^
9	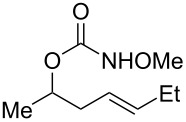	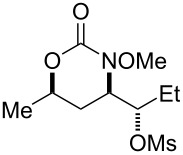	**55**, **56**	80%^a^ dr > 20:1^d^

^a^Reaction conditions: PhI(OH)(OMs) (1.5 equiv), CH_2_Cl_2_, rt. ^b^1-Acetoxy-1,2-benziodoxol-3-(1*H*)-one (1.5 equiv), MeSO_3_H (1.5 equiv), CH_2_Cl_2_, rt; ^c^21% of alkene product arising from mesylate elimination was also isolated. See Supporting Information File 1, compound **S1**. ^d^Relative stereochemistry was determined by nOe correlations. See Supporting Information File 1, Structural Reasoning section.

**Figure 1:**
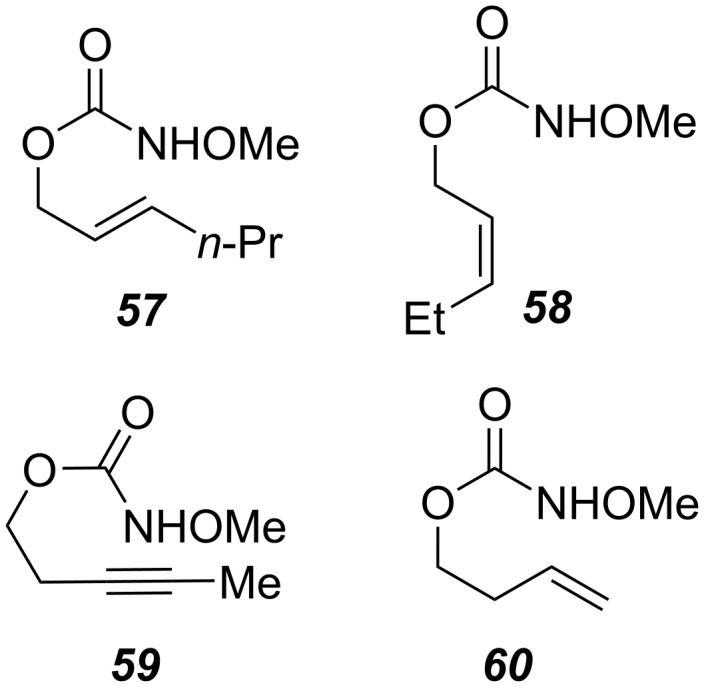
Poor performers.

Surprisingly, carbamate substrates derived from di-substituted allylic alcohols (Figure 1) invariably failed to give the desired amino-sulfonoxylated products. With these substrates, ^1^H NMR analysis of the unpurified reaction residues showed a complex mixture of products. There were some signals suggestive of terminal alkenes, implying that olefin transposition was a competing pathway. With alkyne substrate **59**, decomposition occurred, and the ^1^H NMR of the unpurified reaction mixture was illegible. With terminal alkene substrate **60**, unproductive decomposition of starting material was again observed.

**Scheme 2:**
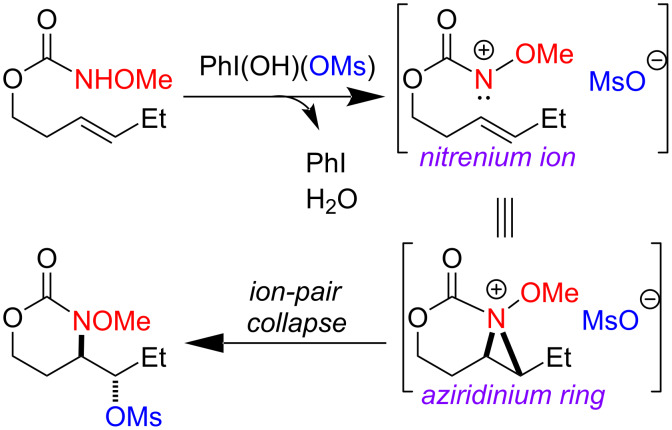
Putative reaction mechanism.

Given the highly predictable diastereoselectivity of this transformation and by analogy to prior art [4,6], we hypothesize that the reaction proceeds through the formation of a transient nitrenium species, which attacks the pendant olefin to form an aziridinium cation (Scheme 2). A sulfonate counter-anion then opens this aziridinium ring in an *exo*-selective, S_N_2 reaction.

**Scheme 3:**
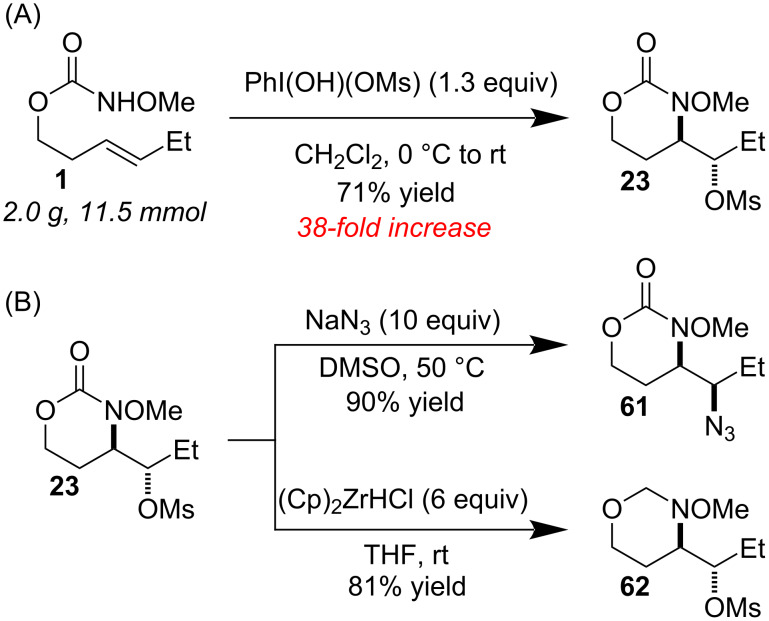
(A) Scale-up and (B) applications.

We were successful in scaling the reaction from 0.3 mmol to 11.5 mmol (38-fold increase) without any erosion in yield or selectivity (Scheme 3A). The mesylate could be cleanly substituted with azide by heating substrate with excess NaN_3_ in DMSO (Scheme 3B). With an excess of Schwartz’s reagent, the carbonyl was cleanly reduced to give 1,3-oxazine **62**. Contrary to what we had initially predicted from literature precedent, there was no trace of amino-alcohol product [34–35]. These transformations are likely difficult or even impossible in a single step with the products from our previously reported amino-trifluoroacetoxylation reaction, highlighting the utility of the present transformation.

## Conclusion

In summary, we have developed a new alkene amino-sulfonoxylation reaction that leverages the unique reactivity of carbamate tethered *N*-alkoxy nitrenium ions. In almost all cases examined, the reactions delivered product with exquisite regioselectivity and diastereoselectivity. The protocols followed were operationally very simple and only used commercial I(III) reagents and sulfonic acids. The products were quite stable in the reaction conditions and withstood standard purification protocols. No special precautions were taken to exclude air or ambient moisture, and the products were amenable to further transformations. We expect this metal-free protocol for alkene amino-oxygenation to find widespread use in both academic and industrial synthetic chemistry.

## Supporting Information

File 1 Additional experimental details including reaction procedures, X-ray crystallographic data, and NMR spectra of synthesized compounds.

## Data Availability

Data generated and analyzed during this study is available from the corresponding author upon reasonable request.
