# Supplementary material for: Harnessing tethered nitreniums for diastereoselective amino-sulfonoxylation of alkenes
Source: Beilstein J Org Chem. 2025 May 19;21:947–54. doi: 10.3762/bjoc.21.78 (PMC12117213; doi:10.3762/bjoc.21.78)

BEILSTEIN JOURNAL OF ORGANIC CHEMISTRY

Supporting Information

for

Harnessing tethered nitreniums for diastereoselective amino-sulfonoxylation of alkenes

Shyam Sathyamoorthi, Appasaheb K. Nirpal, Dnyaneshwar A. Gorve and Steven P. Kelley

Beilstein J. Org. Chem. **2025,** *21,* 947–954. doi:10.3762/bjoc.21.78

Additional experimental details including reaction procedures, X-ray crystallographic data, and NMR spectra of synthesized compounds

License and Terms: This is a supporting information file under the terms of the Creative Commons Attribution License (https://creativecommons.org/licenses/by/4.0). Please note that the reuse, redistribution and reproduction in particular requires that the author(s) and source are credited and that individual graphics may be subject to special legal provisions.
The license is subject to the *Beilstein Journal of Organic Chemistry* terms and conditions: (https://www.beilstein-journals.org/bjoc/terms)

Table of contents

I. General considerations

II. Procedures for syntheses of substrates and products (Tables 2–5 and Figure 1)

III. Characterization of substrates and products (Tables 2–5 and Figure 1)

IV. Procedures for scale-up/applications and product characterization (Scheme 3)

V. Structural reasoning

VI. X-ray crystallographic data

VII. NMR Spectra

I. General considerations

All reagents were obtained commercially unless otherwise noted. Solvents were purified by passage under 10 psi N_2 through activated alumina columns. Infrared (IR) spectra were recorded on a Thermo Scientific™ Nicolet™ iS™5 FT-IR Spectrometer; data are reported in frequency of absorption (cm^{-1}). ^1H NMR spectra were recorded at 400, 500, or 600 MHz. Data are recorded as: chemical shift in ppm referenced internally using residual solvent peaks, multiplicity (s = singlet, br s = broad singlet, d = doublet, t = triplet, q = quartet, m = multiplet or overlap of nonequivalent resonances, qdd = quartet of doublet of doublets, tdt = triplet of doublet of triplets, dtq = doublet of triplet of quartets, qd = quartet of doublets, tdq = triplet of doublet of quartets), integration, coupling constant (Hz). ^{13}C NMR spectra were recorded at 101 or 126 MHz. Exact mass spectra were recorded using an electrospray ion source (ESI) either in positive mode or negative mode and with a time-of-flight (TOF) analyzer on a Waters LCT PremierTM mass spectrometer and are given in m/z. Thin Layer Chromatography (TLC) was performed on pre-coated glass plates (Merck) and visualized either with a UV lamp (254 nm) or by dipping into a solution of $KMnO_4$–K_2CO_3 in water followed by heating. Flash chromatography was performed on silica gel (230–400 mesh) or Florisil (60-100 mesh). “Room temperature” refers to an ambient temperature of 23–25 °C. Structural assignments were made with additional information from gCOSY experiments.

II. Procedures for syntheses of substrates and products (Tables 2–5 and Figure 1)

Substrates were synthesized according to the procedure disclosed in *J. Org. Chem.* 2024, *89*, 15352–15357.

General Procedure A

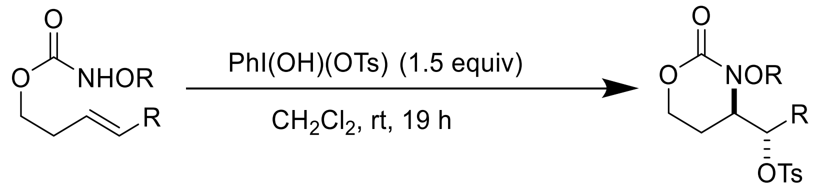

A 10 mL microwave vial equipped with a magnetic stir bar was charged with substrate (0.3 mmol, 1 equiv) and anhydrous CH_2Cl_2 (3 mL). PhI(OH)(OTs) (Koser's reagent) (0.176 g, 0.45 mmol, 1.5 equiv) was added in one bolus. The sides of the vial were rinsed with an additional 3 mL of anhydrous CH_2Cl_2 (final reaction concentration = 0.05 M). The vial was capped, and the reaction was stirred for 19 hours at room temperature. Then, the reaction vial was uncapped, and its contents were transferred to a separatory funnel with EtOAc (25 mL). The organic layer was washed with one portion of saturated, aqueous $Na_2S_2O_3$ solution (10 mL) and one portion of saturated, aqueous $NaHCO_3$ solution (10 mL). The organic layer was collected, dried with $MgSO_4$, filtered, and concentrated under reduced pressure. The resulting residue was purified by chromatography on silica gel (specific conditions are associated with each compound).

General Procedure B

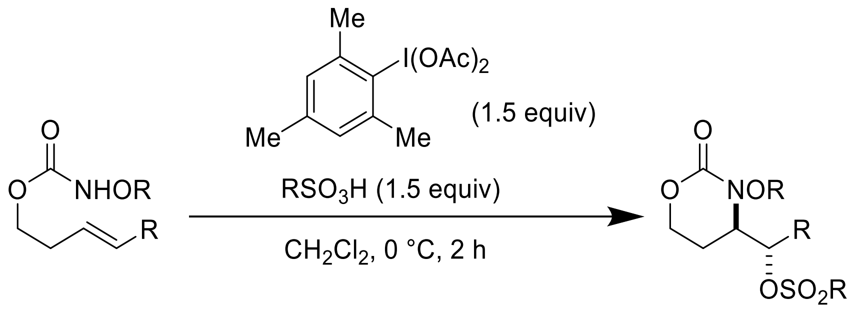

A 10 mL microwave vial equipped with a magnetic stir bar was charged with substrate (0.3 mmol, 1 equiv) and anhydrous CH_2Cl_2 (3 mL). Sulfonic acid (0.45 mmol, 1.5 equiv) was added in one bolus. The reaction flask was cooled to 0 °C using an ice-water bath. Iodomesitylene diacetate (0.164 g, 0.45 mmol, 1.5 equiv) was added in one bolus. The sides of the vial were rinsed with an additional 3 mL of anhydrous CH_2Cl_2 (final reaction concentration = 0.05 M). The vial was capped, and the reaction was stirred for 2 hours at 0 °C. Then, the reaction vial was uncapped, and 2 mL of saturated aqueous $Na_2S_2O_3$ solution was added. After stirring for 2 minutes, the contents of the vial were transferred to a separatory funnel with EtOAc (25 mL). Additional saturated, aqueous $Na_2S_2O_3$ solution (10 mL) was added, and the layers were shaken vigorously. After separating, the aqueous layer was discarded, and the organic layer was washed with one portion of saturated, aqueous $NaHCO_3$ solution (10 mL). The organic layer was collected, dried with $MgSO_4$, filtered, and concentrated under reduced pressure. The resulting residue was purified by chromatography on silica gel (specific conditions are associated with each compound).

Note: For certain substrates, warming the reaction to room temperature over a period of 19–48 h was optimal. Modifications are indicated with characterization data.

General Procedure C

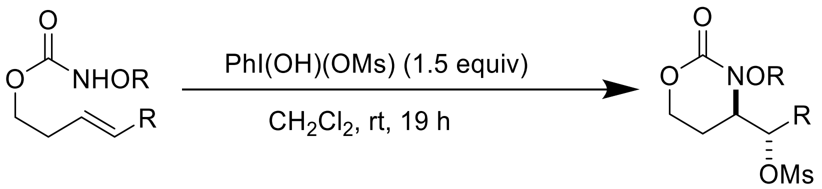

A 10 mL microwave vial equipped with a magnetic stir bar was charged with substrate (0.3 mmol, 1 equiv) and anhydrous CH_2Cl_2 (3 mL). PhI(OH)(OMs) (0.142 g, 0.45 mmol, 1.5 equiv) was added in one bolus. The sides of the vial were rinsed with an additional 3 mL of anhydrous CH_2Cl_2 (final reaction concentration = 0.05 M). The vial was capped, and the reaction was stirred for 19 hours at room temperature. Then, the reaction vial was uncapped, and its contents were transferred to a separatory funnel with EtOAc (25 mL). The organic layer was washed with one portion of saturated, aqueous $Na_2S_2O_3$ solution (10 mL) and one portion of saturated, aqueous $NaHCO_3$ solution (10 mL). The organic layer was collected, dried with $MgSO_4$, filtered, and concentrated under reduced pressure. The resulting residue was purified by chromatography on silica gel (specific conditions are associated with each compound).

General Procedure D

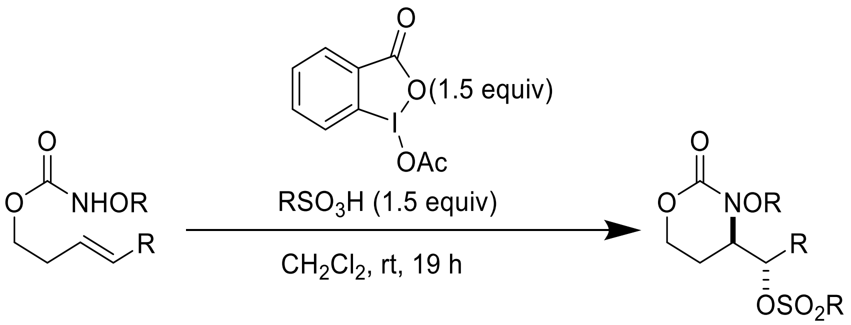

A 10 mL microwave vial equipped with a magnetic stir bar was charged with substrate (0.3 mmol, 1 equiv) and anhydrous CH_2Cl_2 (3 mL). Sulfonic acid (0.45 mmol, 1.5 equiv) was added in one bolus followed by 1-acetoxy-1,2-benziodoxol-3-(1*H*)-one (0.138 g, 0.45 mmol, 1.5 equiv). The sides of the vial were rinsed with an additional 3 mL of anhydrous CH_2Cl_2 (final reaction concentration = 0.05 M). The vial was capped, and the reaction was stirred for 19 hours at room temperature. Then, the reaction vial was uncapped, and its contents were transferred to a separatory funnel with EtOAc (25 mL). Saturated, aqueous $Na_2S_2O_3$ solution (10 mL) was added, and the layers were shaken vigorously. After separating, the aqueous layer was discarded, and the organic layer was washed with saturated, aqueous $NaHCO_3$ solution (2 × 20 mL, required to remove 2-iodobenzoic acid which complicates purification). The organic layer was collected, dried with $MgSO_4$, filtered, and concentrated under reduced pressure. The resulting residue was purified by chromatography on silica gel (specific conditions are associated with each compound).

III. Characterization of substrates and products (Tables 2–5 and Figure 1)

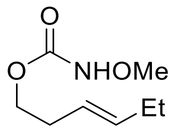

(*E*)-hex-3-en-1-yl methoxycarbamate

Compound 1: Previously characterized in *J. Org. Chem.* **2024**, *89*, 15352–15357.

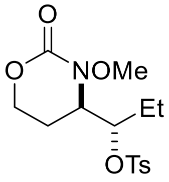

(*S**)-1-((*R**)-3-methoxy-2-oxo-1,3-oxazinan-4-yl)propyl 4-methylbenzenesulfonate

Compound 2: Synthesized using **General Procedure A** on a 0.3 mmol scale; Purified using a gradient of 0 to 100% EtOAc/hexanes followed by a flush of acetone on silica gel; Single diastereomer; (light yellow oil, 0.075 g, 0.218 mmol, 73% yield).

^{1}H NMR (600 MHz, $CDCl_3$) δ 7.77 (d, *J* = 8.0 Hz, 2H), 7.33 (d, *J* = 7.9 Hz, 2H), 4.98 (td, *J* = 6.8, 2.0 Hz, 1H), 4.09 (td, *J* = 10.4, 3.6 Hz, 1H), 4.02 (dt, *J* = 11.1, 4.4 Hz, 1H), 3.79 (s, 3H), 3.74 (ddt, *J* = 7.3, 3.6, 1.7 Hz, 1H), 2.43 (s, 3H), 2.16 – 2.06 (m, 2H), 1.78 (dq, *J* = 14.7, 7.4 Hz, 1H), 1.59 (dp, *J* = 14.3, 7.2 Hz, 1H), 0.93 (t, *J* = 7.5 Hz, 3H).

$^{13}C\{^{1}H\}$ NMR (101 MHz, $CDCl_3$) δ 153.9, 145.1, 134.0, 129.9, 127.6, 82.4, 64.2, 62.0, 59.1, 24.7, 23.0, 21.7, 9.9.

IR ν 1725, 1342, 1171, 1092, 895 cm^{-1}.

HRMS (ESI) m/z = $[M + Na]^+$ Calcd $C_{15}H_{21}NNaO_6S^+$ 366.0982. Found 366.0993 (3 ppm error).

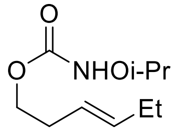

Compound 3: Previously characterized in *J. Org. Chem.* **2024**, *89*, 15352–15357.

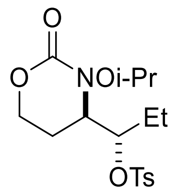

(*S**)-1-((*R**)-3-isopropoxy-2-oxo-1,3-oxazinan-4-yl)propyl 4-methylbenzenesulfonate

Compound 4:

(1) Synthesized using **General Procedure A** on a 0.3 mmol scale; Purified using a gradient of 0 to 100% EtOAc/hexanes followed by a flush of acetone on silica gel; Single diastereomer; (light yellow oil, 0.038 g, 0.102 mmol, 34% yield).

(2) Synthesized using **General Procedure B** on a 0.2 mmol scale; (light yellow oil, 0.042 g, 0.113 mmol, 57% yield).

^{1}H NMR (400 MHz, $CDCl_3$) δ 7.83 – 7.74 (m, 2H), 7.33 (d, *J* = 8.1 Hz, 2H), 4.89 (dt, *J* = 6.8, 3.4 Hz, 1H), 4.24 (dq, *J* = 12.5, 6.3 Hz, 1H), 4.17 (ddd, *J* = 11.0, 6.4, 4.6 Hz, 1H), 4.06 (ddd, *J* = 11.2, 7.0, 4.6 Hz, 1H), 3.93 (td, *J* = 7.0, 2.5 Hz, 1H), 2.43 (s, 3H), 2.17 – 2.09 (m, 2H), 2.05 – 1.94 (m, 1H), 1.64 (dt, *J* = 14.4, 7.2 Hz, 1H), 1.20 – 1.15 (m, 6H), 0.92 (t, *J* = 7.5 Hz, 3H).

$^{13}C\{^1H\}$ NMR (101 MHz, $CDCl_3$) δ 153.8, 145.0, 133.7, 129.9, 128.0, 81.4, 76.5, 63.7, 60.1, 24.2, 23.8, 21.8, 21.0, 20.8, 9.8.

IR ν 1718, 1353, 1174, 1096, 870 cm^{-1}.

HRMS (ESI) m/z = $[M + Na]^+$ Calcd $C_{17}H_{25}NNaO_6S^+$ 394.1295. Found 394.1299 (1.0 ppm error).

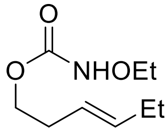

(*E*)-hex-3-en-1-yl ethoxycarbamate

Compound 5: Previously characterized in *J. Org. Chem.* **2024**, *89*, 15352–15357.

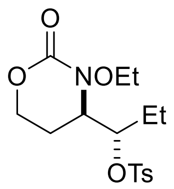

(S*)-1-((R*)-3-ethoxy-2-oxo-1,3-oxazinan-4-yl)propyl 4-methylbenzenesulfonate

Compound 6: Synthesized using **General Procedure A** on a 0.222 mmol scale; Purified using a gradient of 0 to 100% EtOAc/hexanes followed by a flush of acetone on silica gel; Single diastereomer; (light yellow oil, 0.063 g, 0.176 mmol, 79% yield).

^{1}H NMR (400 MHz, $CDCl_3$) δ 7.83 – 7.68 (m, 2H), 7.33 (d, *J* = 8.2 Hz, 2H), 4.93 (td, *J* = 6.8, 2.1 Hz, 1H), 4.12 – 3.93 (m, 4H), 3.77 (ddd, *J* = 6.9, 4.3, 2.2 Hz, 1H), 2.43 (s, 3H), 2.10 (ddd, *J* = 8.3, 5.1, 3.9 Hz, 2H), 1.83 (dq, *J* = 14.6, 7.3 Hz, 1H), 1.61 (dp, *J* = 14.4, 7.3 Hz, 1H), 1.26 – 1.17 (m, 3H), 0.94 (t, *J* = 7.5 Hz, 3H).

^{13}C{^{1}H} NMR (101 MHz, $CDCl_3$) δ 153.9, 145.1, 134.0, 129.9, 127.7, 82.3, 69.9, 64.1, 59.2, 24.6, 23.2, 21.7, 13.5, 9.9.

IR ν 1721, 1346, 1175, 1093, 904 cm^{-1}.

HRMS (ESI) m/z = $[M + Na]^+$ Calcd $C_{16}H_{23}NNaO_6S^+$ 380.1138. Found 380.1143 (1.3 ppm error).

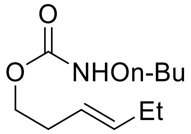

(E)-hex-3-en-1-yl butoxycarbamate

Compound 7: Previously characterized in *J. Org. Chem.* **2024**, *89*, 15352–15357.

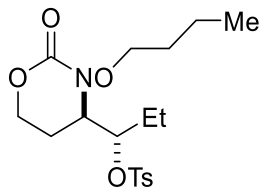

(S*)-1-((R*)-3-butoxy-2-oxo-1,3-oxazinan-4-yl)propyl 4-methylbenzenesulfonate

Compound 8: Synthesized using **General Procedure A** on a 0.3 mmol scale; Purified using a gradient of 0 to 100% EtOAc/hexanes on silica gel; Single diastereomer; (light yellow oil, 0.086 g, 0.223 mmol, 74% yield).

^{1}H NMR (400 MHz, $CDCl_3$) δ 7.84 – 7.66 (m, 2H), 7.33 (d, J = 8.1 Hz, 2H), 4.93 (td, J = 6.9, 2.1 Hz, 1H), 4.14 – 4.06 (m, 1H), 4.06 – 3.97 (m, 1H), 3.93 (t, J = 6.6 Hz, 2H), 3.77 (dq, J = 5.8, 2.0 Hz, 1H), 2.43 (s, 3H), 2.18 – 2.00 (m, 2H), 1.86 (dp, J = 14.6, 7.4 Hz, 1H), 1.69 – 1.52 (m, 3H), 1.39 (h, J = 7.3 Hz, 2H), 0.97 – 0.90 (m, 6H).

^{13}C{^{1}H} NMR (101 MHz, $CDCl_3$) δ 153.7, 145.1, 133.9, 130.0, 127.7, 82.2, 74.0, 64.1, 59.0, 30.2, 24.7, 23.1, 21.7, 19.2, 14.0, 9.9.

IR ν 1721, 1346, 1174, 1099, 907 cm^{-1}.

HRMS (ESI) m/z = $[M + Na]^+$ Calcd $C_{18}H_{27}NNaO_6S^+$ 408.1451. Found 408.1456 (1.2 ppm error).

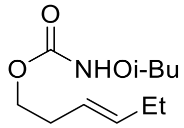

(E)-hex-3-en-1-yl isobutoxycarbamate

Compound 9: Previously characterized in *J. Org. Chem.* **2024**, *89*, 15352–15357.

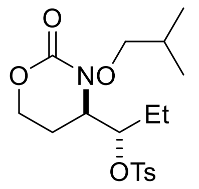

(*S**)-1-((*R**)-3-isobutoxy-2-oxo-1,3-oxazinan-4-yl)propyl 4-methylbenzenesulfonate

Compound 10: Synthesized using **General Procedure A** on a 0.3 mmol scale; Purified using a gradient of 0 to 100% EtOAc/hexanes on silica gel; Single diastereomer; (light yellow oil, 0.097 g, 0.252 mmol, 84% yield).

^{1}H NMR (400 MHz, $CDCl_3$) δ 7.89 – 7.68 (m, 2H), 7.33 (d, *J* = 8.1 Hz, 2H), 4.93 (ddd, *J* = 8.0, 6.4, 1.9 Hz, 1H), 4.15 – 4.08 (m, 1H), 4.03 (dt, *J* = 11.0, 4.5 Hz, 1H), 3.76 (ddd, *J* = 8.0, 3.9, 2.0 Hz, 1H), 3.73 – 3.59 (m, 2H), 2.43 (s, 3H), 2.18 – 2.03 (m, 2H), 1.99 – 1.82 (m, 2H), 1.64 (dq, *J* = 14.5, 7.4 Hz, 1H), 1.01 – 0.86 (m, 9H).

^{13}C{^{1}H} NMR (101 MHz, $CDCl_3$) δ 153.6, 145.1, 133.9, 130.0, 127.8, 82.2, 80.2, 64.1, 58.9, 27.4, 24.7, 23.0, 21.7, 19.32, 19.29, 10.0.

IR ν 1725, 1351, 1276, 1174, 1096, 868 cm^{-1}.

HRMS (ESI) m/z = $[M + Na]^+$ Calcd $C_{18}H_{27}NNaO_6S^+$ 408.1451. Found 408.1456 (1.2 ppm error).

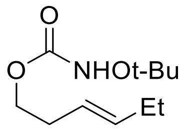

(*E*)-hex-3-en-1-yl *tert*-butoxycarbamate

Compound 11: Previously characterized in *J. Org. Chem.* **2024**, *89*, 15352–15357.

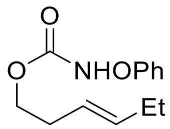

(*E*)-hex-3-en-1-yl phenoxycarbamate

Compound 12: Previously characterized in *J. Org. Chem.* **2024**, *89*, 15352–15357.

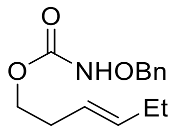

(*E*)-hex-3-en-1-yl (benzyloxy)carbamate

Compound 13: Previously characterized in *J. Org. Chem*. **2024**, *89*, 15352–15357.

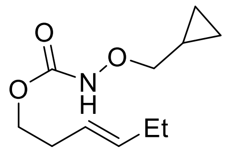

(*E*)-hex-3-en-1-yl (cyclopropylmethoxy)carbamate

Compound 14: Previously characterized in *J. Org. Chem*. **2024**, *89*, 15352–15357.

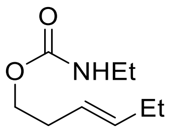

(*E*)-hex-3-en-1-yl ethylcarbamate

Compound 15: Previously characterized in *J. Org. Chem*. **2024**, *89*, 15352–15357.

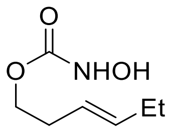

(*E*)-hex-3-en-1-yl hydroxycarbamate

Compound 16: Previously characterized in *J. Org. Chem*. **2024**, *89*, 15352–15357.

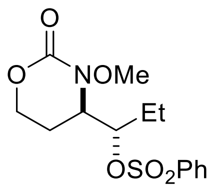

(S*)-1-((R*)-3-methoxy-2-oxo-1,3-oxazinan-4-yl)propyl benzenesulfonate

Compound 17: Synthesized using **General Procedure D** on a 0.3 mmol scale; Purified using a gradient of 0 to 100% EtOAc/hexanes on silica gel; Single diastereomer; (light yellow oil, 0.049 g, 0.149 mmol, 50% yield).

^{1}H NMR (400 MHz, $CDCl_3$) δ 7.95 – 7.82 (m, 2H), 7.73 – 7.62 (m, 1H), 7.56 (dd, J = 8.5, 7.1 Hz, 2H), 5.02 (td, J = 6.9, 2.0 Hz, 1H), 4.18 – 3.97 (m, 2H), 3.79 (s, 3H), 3.75 (ddd, J = 7.7, 3.9, 2.0 Hz, 1H), 2.12 (tdd, J = 9.7, 8.1, 5.4 Hz, 2H), 1.88 – 1.74 (m, 1H), 1.62 (dp, J = 14.4, 7.3 Hz, 1H), 0.95 (t, J = 7.5 Hz, 3H).

^{13}C{^{1}H} NMR (101 MHz, $CDCl_3$) δ 153.8, 137.0, 134.1, 129.4, 127.6, 82.6, 64.2, 62.0, 59.0, 24.7, 23.0, 9.9.

IR ν 1717, 1343, 1273, 1185, 1096, 906, 749 cm^{-1}.

HRMS (ESI) m/z = $[M + Na]^+$ Calcd $C_{14}H_{19}NNaO_6S^+$ 352.0825. Found 352.0829 (1.1 ppm error).

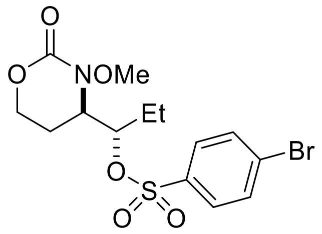

(S*)-1-((R*)-3-methoxy-2-oxo-1,3-oxazinan-4-yl)propyl 4-bromobenzenesulfonate

Compound 18: Synthesized using **General Procedure D** on a 0.3 mmol scale; Purified using a gradient of 0 to 100% EtOAc/hexanes on silica gel; Single diastereomer; (light yellow oil, 0.076 g, 0.186 mmol, 62% yield).

^{1}H NMR (400 MHz, $CDCl_3$) δ 7.77 – 7.73 (m, 2H), 7.72 – 7.65 (m, 2H), 5.04 (td, J = 7.0, 2.0 Hz, 1H), 4.17 – 4.00 (m, 2H), 3.87 – 3.69 (m, 4H), 2.18 – 2.07 (m, 2H), 1.82 (dt, J = 14.7, 7.4 Hz, 1H), 1.60 (dt, J = 14.4, 7.2 Hz, 1H), 0.95 (t, J = 7.5 Hz, 3H).

^{13}C{^{1}H} NMR (101 MHz, $CDCl_3$) δ 153.6, 136.0, 132.7, 129.3, 129.2, 82.7, 64.1, 62.0, 58.9, 24.7, 22.9, 10.0.

IR ν 1718, 1350, 1185, 904, 749 cm^{-1}.

HRMS (ESI) m/z = $[M + Na]^+$ Calcd $C_{14}H_{18}BrNNaO_6S^+$ 429.9930. Found 429.9955 (5.8 ppm error).

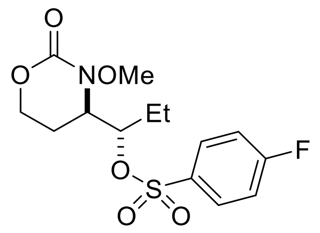

(*S**)-1-((*R**)-3-methoxy-2-oxo-1,3-oxazinan-4-yl)propyl 4-fluorobenzenesulfonate

Compound 19: Synthesized using **General Procedure D** on a 0.3 mmol scale; Purified using a gradient of 0 to 100% EtOAc/hexanes on silica gel; Single diastereomer; (light yellow oil, 0.064 g, 0.184 mmol, 61% yield).

^{1}H NMR (400 MHz, $CDCl_3$) δ 7.99 – 7.79 (m, 2H), 7.27 – 7.13 (m, 2H), 5.04 (td, *J* = 6.9, 2.0 Hz, 1H), 4.07 (dddd, *J* = 15.8, 11.1, 8.3, 3.7 Hz, 2H), 3.83 – 3.73 (m, 4H), 2.13 (tt, *J* = 12.1, 3.8 Hz, 2H), 1.84 (dq, *J* = 14.7, 7.5 Hz, 1H), 1.62 (dp, *J* = 14.3, 7.3 Hz, 1H), 0.96 (t, *J* = 7.5 Hz, 3H).

^{13}C{^{1}H} NMR (101 MHz, $CDCl_3$) δ 165.9 (d, *J* = 256.9 Hz), 153.5, 133.0 (d, *J* = 3.2 Hz), 130.6 (d, *J* = 9.6 Hz), 116.7 (d, *J* = 22.9 Hz), 82.5, 64.1, 61.9, 58.8, 24.7, 22.9, 10.0.

^{19}F{^{1}H} NMR (377 MHz, $CDCl_3$) δ -102.54.

IR ν 1716, 1350, 1181, 1093, 901, 840 cm^{-1}.

HRMS (ESI) m/z = $[M + Na]^+$ Calcd $C_{14}H_{18}FNNaO_6S^+$ 370.0731. Found 370.0739 (2.2 ppm error).

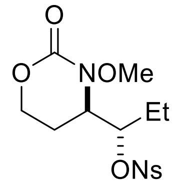

(*S**)-1-((*R**)-3-methoxy-2-oxo-1,3-oxazinan-4-yl)propyl 4-nitrobenzenesulfonate

Compound 20: Synthesized using a modified version of **General Procedure B** (0 °C to 23 °C over a period of 48 hours) on a 0.3 mmol scale; Purified using a gradient of 0 to 100% EtOAc/hexanes on silica gel; Single diastereomer; (light yellow oil which crystallizes upon standing, 0.074 g, 0.198 mmol, 66% yield).

^{1}H NMR (400 MHz, $CDCl_3$) δ 8.50 – 8.28 (m, 2H), 8.25 – 7.94 (m, 2H), 5.14 (td, *J* = 7.0, 2.0 Hz, 1H), 4.14 – 4.10 (m, 1H), 4.10 – 3.99 (m, 1H), 3.85 (ddd, *J* = 7.6, 5.6, 2.0 Hz, 1H), 3.74 (s, 3H), 2.23 – 2.07 (m, 2H), 1.88 (dq, *J* = 15.0, 7.5 Hz, 1H), 1.68 (dq, *J* = 14.3, 7.1 Hz, 1H), 1.01 (t, *J* = 7.5 Hz, 3H).

^{13}C{^{1}H} NMR (101 MHz, $CDCl_3$) δ 153.0, 150.9, 142.4, 129.2, 124.6, 83.0, 64.0, 61.7, 58.4, 24.8, 22.8, 10.1.

IR ν 1716, 1529, 1349, 1183, 901, 853 cm^{-1}.

HRMS (ESI) m/z = $[M + Na]^+$ Calcd $C_{14}H_{18}N_2NaO_8S^+$ 397.0676. Found 397.0651 (6.3 ppm error).

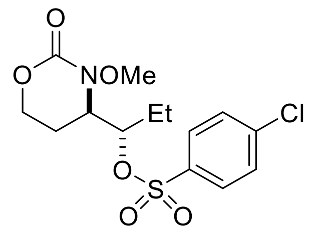

(*S**)-1-((*R**)-3-methoxy-2-oxo-1,3-oxazinan-4-yl)propyl 4-chlorobenzenesulfonate

Compound 21: Synthesized using **General Procedure D** on a 0.3 mmol scale; Purified using a gradient of 0 to 100% EtOAc/hexanes on silica gel; Single diastereomer; (light yellow oil, 0.058 g, 0.159 mmol, 53% yield).

^{1}H NMR (400 MHz, $CDCl_3$) δ 7.95 – 7.74 (m, 2H), 7.57 – 7.49 (m, 2H), 5.05 (td, *J* = 6.9, 1.9 Hz, 1H), 4.19 – 3.95 (m, 2H), 3.86 – 3.73 (m, 4H), 2.17 – 2.05 (m, 2H), 1.84 (dq, *J* = 14.7, 7.5 Hz, 1H), 1.64 (dq, *J* = 14.3, 7.1 Hz, 1H), 0.97 (t, *J* = 7.5 Hz, 3H).

^{13}C{^{1}H} NMR (101 MHz, $CDCl_3$) δ 153.6, 140.8, 135.5, 129.7, 129.2, 82.6, 64.1, 62.0, 58.9, 24.7, 23.0, 10.0.

IR ν 1718, 1347, 1184, 1086, 901, 870, 761 cm^{-1}.

HRMS (ESI) m/z = $[M + Na]^+$ Calcd $C_{14}H_{18}ClNNaO_6S^+$ 386.0436. Found 386.0412 (6.2 ppm error).

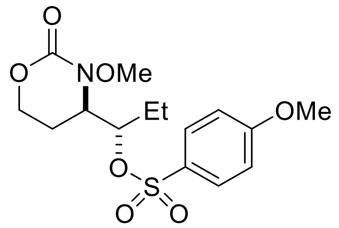

(*S**)-1-((*R**)-3-methoxy-2-oxo-1,3-oxazinan-4-yl)propyl 4-methoxybenzenesulfonate

Compound 22: Synthesized using **General Procedure D** on a 0.3 mmol scale; Purified using a gradient of 0 to 100% EtOAc/hexanes on silica gel; Single diastereomer; (light yellow oil, 0.065 g, 0.181 mmol, 60% yield).

^{1}H NMR (400 MHz, $CDCl_3$) δ 7.95 – 7.68 (m, 2H), 7.10 – 6.86 (m, 2H), 4.97 (td, *J* = 6.9, 2.0 Hz, 1H), 4.07 (dtd, *J* = 21.2, 8.1, 4.0 Hz, 2H), 3.87 (s, 3H), 3.80 (s, 3H), 3.78 – 3.71 (m, 1H), 2.19 – 2.04 (m, 2H), 1.79 (dp, *J* = 14.3, 7.0 Hz, 1H), 1.60 (dp, *J* = 14.4, 7.2 Hz, 1H), 0.94 (t, *J* = 7.5 Hz, 3H).

^{13}C{^{1}H} NMR (101 MHz, $CDCl_3$) δ 163.9, 153.9, 129.9, 128.4, 114.5, 82.1, 64.2, 62.0, 59.0, 55.8, 24.7, 23.0, 9.9.

IR ν 1718, 1343, 1260, 1165, 1102, 903 cm^{-1}.

HRMS (ESI) m/z = $[M + Na]^+$ Calcd $C_{15}H_{21}NNaO_7S^+$ 382.0931. Found 382.0909 (5.8 ppm error).

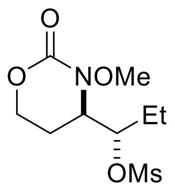

(S*)-1-((R*)-3-methoxy-2-oxo-1,3-oxazinan-4-yl)propyl methanesulfonate

Compound 23: Synthesized using **General Procedure C** on a 0.3 mmol scale; Purified using a gradient of 0 to 100% EtOAc/hexanes on silica gel followed by a gradient of 10 to 50% acetone in EtOAc on silica gel; Single diastereomer; (light yellow oil, 0.056 g, 0.210 mmol, 70% yield).

^{1}H NMR (400 MHz, $CDCl_3$) δ 5.04 (ddd, J = 7.6, 6.5, 2.1 Hz, 1H), 4.26 (ddd, J = 10.5, 6.2, 4.0 Hz, 1H), 4.12 (ddd, J = 11.1, 8.6, 3.6 Hz, 1H), 3.93 (dd, J = 6.4, 2.1 Hz, 1H), 3.80 (s, 3H), 3.06 (s, 3H), 2.25 (dddd, J = 14.9, 8.6, 6.5, 4.0 Hz, 1H), 2.21 – 2.11 (m, 1H), 1.88 (dt, J = 14.1, 7.5 Hz, 1H), 1.64 (dp, J = 14.4, 7.3 Hz, 1H), 1.05 (t, J = 7.6 Hz, 3H).

^{13}C{^{1}H} NMR (101 MHz, $CDCl_3$) δ 153.9, 80.8, 64.1, 62.0, 58.4, 39.2, 25.0, 22.8, 10.0.

IR ν 1716, 1339, 1171, 931, 904, 873 cm^{-1}.

HRMS (ESI) m/z = $[M + Na]^+$ Calcd $C_9H_{17}NNaO_6S^+$ 290.0669. Found 290.0654 (5.2 ppm error).

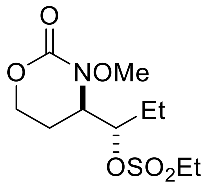

(S*)-1-((R*)-3-methoxy-2-oxo-1,3-oxazinan-4-yl)propyl ethanesulfonate

Compound 24: Synthesized using **General Procedure D** on a 0.3 mmol scale; Purified using a gradient of 0 to 100% EtOAc/hexanes on silica gel followed by a flush of 30% acetone/EtOAc; Single diastereomer; (light yellow oil, 0.051 g, 0.181 mmol, 60% yield).

^{1}H NMR (400 MHz, $CDCl_3$) δ 5.04 (td, J = 7.0, 2.1 Hz, 1H), 4.26 (ddd, J = 10.8, 6.7, 3.9 Hz, 1H), 4.12 (ddd, J = 11.3, 7.9, 3.6 Hz, 1H), 3.91 (ddd, J = 8.1, 6.1, 2.1 Hz, 1H), 3.81 (s, 3H), 3.17 (q, J = 7.5 Hz, 2H), 2.24 (ddq, J = 10.3, 6.2, 2.2 Hz, 1H), 2.20 – 2.12 (m, 1H), 1.89 (dp, J = 14.9, 7.5 Hz, 1H), 1.64 (dd, J = 14.3, 7.2 Hz, 1H), 1.39 (t, J = 7.4 Hz, 3H), 1.05 (t, J = 7.5 Hz, 3H).

^{13}C{^{1}H} NMR (101 MHz, $CDCl_3$) δ 153.8, 80.4, 64.1, 62.0, 58.5, 46.6, 25.1, 22.9, 10.0, 8.2.

IR ν 1716, 1341, 1163, 1106, 904, 871 cm^{-1}.

HRMS (ESI) m/z = $[M + Na]^+$ Calcd $C_{10}H_{19}NNaO_6S^+$ 304.0825. Found 304.0825 (0 ppm error).

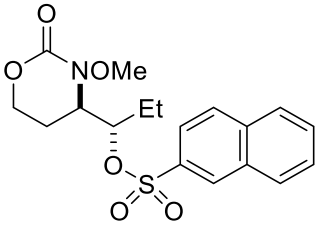

(S*)-1-((R*)-3-methoxy-2-oxo-1,3-oxazinan-4-yl)propyl naphthalene-2-sulfonate

Compound 25: Synthesized using a modified version of **General Procedure B** (0 °C to 23 °C over a period of 19 hours) on a 0.3 mmol scale; Purified using a gradient of 0 to 100% EtOAc/hexanes on silica gel; Single diastereomer; (light yellow oil, 0.061 g, 0.161 mmol, 54% yield).

^{1}H NMR (600 MHz, $CDCl_3$) δ 8.49 (d, J = 1.9 Hz, 1H), 7.99 (t, J = 9.0 Hz, 2H), 7.92 (d, J = 8.2 Hz, 1H), 7.85 (dd, J = 8.6, 2.0 Hz, 1H), 7.68 (t, J = 7.2 Hz, 1H), 7.66 – 7.63 (m, 1H), 5.08 (td, J = 6.9, 2.1 Hz, 1H), 4.14 (td, J = 10.5, 3.3 Hz, 1H), 4.03 (dt, J = 11.0, 4.3 Hz, 1H), 3.84 – 3.73 (m, 4H), 2.19 – 2.08 (m, 2H), 1.82 (dp, J = 14.7, 7.4 Hz, 1H), 1.63 (dq, J = 14.4, 7.2 Hz, 1H), 0.94 (t, J = 7.5 Hz, 3H).

^{13}C{^{1}H} NMR (101 MHz, $CDCl_3$) δ 154.0, 135.4, 133.8, 132.0, 129.8, 129.6, 129.4, 128.1, 128.0, 122.3, 82.6, 64.1, 62.1, 59.2, 24.7, 23.0, 9.9.

IR ν 1716, 1342, 1174, 1076, 901, 869, 749 cm^{-1}.

HRMS (ESI) m/z = $[M + Na]^+$ Calcd $C_{18}H_{21}NNaO_6S^+$ 402.0982. Found 402.0955 (6.7 ppm error).

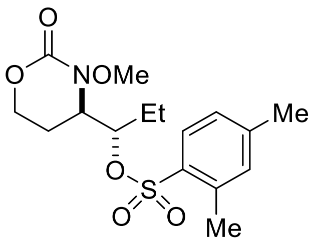

(S*)-1-((R*)-3-methoxy-2-oxo-1,3-oxazinan-4-yl)propyl 2,4-dimethylbenzenesulfonate

Compound 26: Synthesized using a modified version of **General Procedure B** (0 °C to 23 °C over a period of 19 h) on a 0.3 mmol scale; Purified using a gradient of 0 to 100% EtOAc/hexanes on silica gel; Single diastereomer; (light yellow oil, 0.067 g, 0.188 mmol, 63% yield).

^{1}H NMR (400 MHz, $CDCl_3$) δ 7.82 (d, J = 7.9 Hz, 1H), 7.22 – 7.02 (m, 2H), 4.92 (td, J = 6.8, 2.2 Hz, 1H), 4.26 – 4.15 (m, 1H), 4.09 (dt, J = 11.1, 4.3 Hz, 1H), 3.87 – 3.69 (m, 4H), 2.60 (s, 3H), 2.38 (s, 3H), 2.22 – 2.08 (m, 2H), 1.78 (dp, J = 14.4, 7.4 Hz, 1H), 1.60 (dt, J = 14.4, 7.3 Hz, 1H), 0.87 (t, J = 7.5 Hz, 3H).

^{13}C{^{1}H} NMR (101 MHz, $CDCl_3$) δ 154.1, 144.9, 137.9, 133.5, 132.9, 129.3, 127.0, 82.4, 64.1, 62.2, 59.2, 24.4, 23.3, 21.5, 20.4, 9.8.

IR ν 1720, 1341, 1179, 933, 901, 864 cm^{-1}.

HRMS (ESI) m/z = $[M + Na]^+$ Calcd $C_{16}H_{23}NNaO_6S^+$ 380.1138. Found 380.1136 (0.5 ppm error).

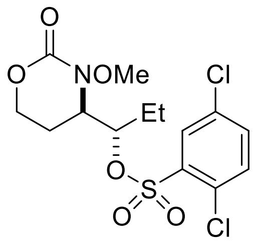

(S*)-1-((R*)-3-methoxy-2-oxo-1,3-oxazinan-4-yl)propyl 2,5-dichlorobenzenesulfonate

Compound 27: Synthesized using a modified version of **General Procedure B** (0 °C to 23 °C over a period of 19 h) on a 0.3 mmol scale; Purified using a gradient of 0 to 100% EtOAc/hexanes on silica gel; Single diastereomer; (light yellow oil, 0.074 g, 0.186 mmol, 62% yield).

^{1}H NMR (400 MHz, $CDCl_3$) δ 8.02 (d, J = 2.2 Hz, 1H), 7.61 – 7.38 (m, 2H), 5.05 (td, J = 7.0, 2.0 Hz, 1H), 4.31 (ddd, J = 10.9, 9.2, 3.3 Hz, 1H), 4.14 – 4.05 (m, 1H), 3.79 (ddd, J = 8.1, 4.5, 2.0 Hz, 1H), 3.70 (s, 3H), 2.24 (ddt, J = 14.7, 5.5, 3.6 Hz, 1H), 2.13 (dddd, J = 14.3, 9.2, 7.9, 4.0 Hz, 1H), 1.89 (dp, J = 14.7, 7.4 Hz, 1H), 1.74 – 1.60 (m, 1H), 0.97 (t, J = 7.5 Hz, 3H).

^{13}C{^{1}H} NMR (101 MHz, $CDCl_3$) δ 153.3, 136.4, 134.7, 133.4, 133.3, 130.9, 130.8, 83.8, 64.1, 61.8, 58.7, 24.7, 22.9, 10.0.

IR ν 1741, 1346, 1181, 1042, 884, 728 cm^{-1}.

HRMS (ESI) m/z = $[M + Na]^+$ Calcd $C_{14}H_{17}Cl_2NNaO_6S^+$ 420.0046. Found 420.0043 (0.7 ppm error).

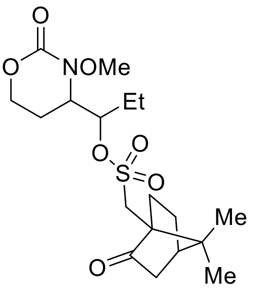

1-(3-methoxy-2-oxo-1,3-oxazinan-4-yl)propyl ((1*S*,4*S*)-7,7-dimethyl-2-oxobicyclo[2.2.1]heptan-1-yl)methanesulfonate

Compound 28: Synthesized using a modified version of **General Procedure B** (0 °C to 23 °C over a period of 19 h) on a 0.3 mmol scale; Purified using a gradient of 0 to 100% EtOAc/hexanes on silica gel; ~1:1 mixture of diastereomers; (light yellow oil, 0.085 g, 0.211 mmol, 70% yield).

Note: Characterization is for mixture of diastereomers

^{1}H NMR (600 MHz, $CDCl_3$) δ 5.10 (td, *J* = 7.0, 2.1 Hz, 2H), 4.29 (tdd, *J* = 11.1, 7.5, 3.7 Hz, 2H), 4.20 – 4.07 (m, 2H), 3.89 (tdd, *J* = 6.2, 4.8, 2.3 Hz, 2H), 3.83 (s, 6H), 3.76 (d, *J* = 14.7 Hz, 1H), 3.56 (d, *J* = 14.7 Hz, 1H), 3.17 (d, *J* = 14.7 Hz, 1H), 3.00 (d, *J* = 14.8 Hz, 1H), 2.38 (ddd, *J* = 17.7, 7.7, 4.0 Hz, 4H), 2.28 – 2.20 (m, 2H), 2.15 (qd, *J* = 7.3, 3.4 Hz, 2H), 2.11 (q, *J* = 4.2 Hz, 2H), 2.04 (tq, *J* = 12.2, 3.8 Hz, 2H), 2.00 – 1.90 (m, 3H), 1.89 – 1.83 (m, 1H), 1.78 – 1.59 (m, 4H), 1.46 – 1.40 (m, 2H), 1.13 – 1.03 (m, 12H), 0.89 (s, 6H).

^{13}C{^{1}H} NMR (101 MHz, $CDCl_3$) δ 214.1, 213.9, 154.3, 153.8, 81.0, 80.9, 64.2, 64.1, 62.3, 62.1, 59.0, 58.8, 58.07, 58.01, 48.9, 48.32, 48.30, 42.9, 42.8, 42.7, 27.1, 25.2, 25.16, 25.12, 25.08, 23.0, 19.7, 19.6, 10.2, 10.1.

IR ν 1731, 1356, 1175, 1108, 904, 726 cm^{-1}.

HRMS (ESI) m/z = $[M + Na]^+$ Calcd $C_{18}H_{29}NNaO_7S^+$ 426.1557. Found 426.1551 (1.4 ppm error).

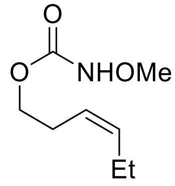

(*Z*)-hex-3-en-1-yl methoxycarbamate

Compound 29: Previously characterized in *J. Org. Chem.* **2024**, *89*, 15352–15357.

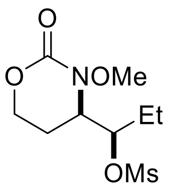

(R*)-1-((R*)-3-methoxy-2-oxo-1,3-oxazinan-4-yl)propyl methanesulfonate

Compound 30: Synthesized using **General Procedure C** on a 0.3 mmol scale; Purified using a gradient of 0 to 100% EtOAc/hexanes on silica gel; Single diastereomer; (light yellow oil, 0.058 g, 0.217 mmol, 72% yield).

^{1}H NMR (400 MHz, $CDCl_3$) δ 4.94 – 4.86 (m, 1H), 4.33 (td, J = 7.4, 4.2 Hz, 1H), 4.25 – 4.07 (m, 2H), 3.76 (s, 3H), 3.07 (s, 3H), 2.19 (tddt, J = 18.9, 14.6, 11.9, 4.2 Hz, 2H), 1.79 (dqd, J = 14.9, 7.5, 2.8 Hz, 1H), 1.73 – 1.54 (m, 1H), 1.06 (t, J = 7.3 Hz, 3H).

$^{13}C\{^1H\}$ NMR (101 MHz, $CDCl_3$) δ 153.4, 82.0, 64.4, 61.7, 57.9, 38.4, 23.5, 21.5, 10.4.

IR ν 1725, 1331, 1169, 924, 858 cm^{-1}.

HRMS (ESI) m/z = $[M + Na]^+$ Calcd $C_9H_{17}NNaO_6S^+$ 290.0669. Found 290.0643 (9 ppm error).

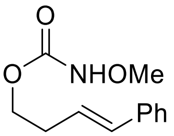

(E)-4-phenylbut-3-en-1-yl methoxycarbamate

Compound 31: Previously characterized in *J. Org. Chem.* **2024**, *89*, 15352–15357.

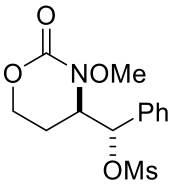

(S*)-((R*)-3-methoxy-2-oxo-1,3-oxazinan-4-yl)(phenyl)methyl methanesulfonate

Compound 32: Synthesized using **General Procedure D** on a 0.3 mmol scale; Purified using a gradient of 0 to 100% EtOAc/hexanes on silica gel; Single diastereomer; (light yellow oil, 0.057 g, 0.181 mmol, 60% yield).

^{1}H NMR (600 MHz, $CDCl_3$) δ 7.47 – 7.32 (m, 5H), 6.08 (d, J = 2.9 Hz, 1H), 4.27 (ddd, J = 11.0, 7.0, 3.6 Hz, 1H), 4.12 – 4.01 (m, 2H), 3.84 (s, 3H), 3.03 (s, 3H), 2.28 – 2.21 (m, 1H), 1.84 (dtd, J = 15.0, 7.5, 3.3 Hz, 1H).

$^{13}C\{^1H\}$ NMR (101 MHz, $CDCl_3$) δ 154.0, 135.0, 129.1, 125.8, 79.7, 64.1, 62.4, 61.6, 39.5, 22.6.

IR ν 1716, 1353, 1175, 1108, 957, 863 cm^{-1}.

HRMS (ESI) m/z = $[M + H]^+$ Calcd $C_{13}H_{18}NO_6S^+$ 316.0849. Found 316.0825 (7.6 ppm error).

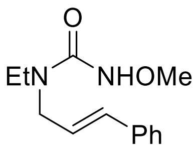

Compound 33: Previously characterized in *J. Org. Chem.* **2024**, *89*, 15352–15357.

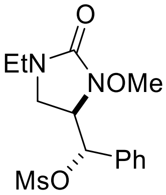

(*S**)-((*R**)-1-ethyl-3-methoxy-2-oxoimidazolidin-4-yl)(phenyl)methyl methanesulfonate

Compound 34: Synthesized using **General Procedure C** on a 0.3 mmol scale; Purified using a gradient of 0 to 100% EtOAc/hexanes on silica gel; Single diastereomer; (light yellow oil, 0.052 g, 0.158 mmol, 53% yield).

^{1}H NMR (600 MHz, $CDCl_3$) δ 7.56 – 7.33 (m, 5H), 5.80 (d, *J* = 5.0 Hz, 1H), 3.88 (dt, *J* = 6.4, 4.3 Hz, 1H), 3.62 (s, 3H), 3.36 (t, *J* = 8.9 Hz, 1H), 3.34 – 3.28 (m, 2H), 3.24 (p, *J* = 7.1 Hz, 1H), 2.80 (s, 3H), 1.11 (t, *J* = 7.3 Hz, 3H).

^{13}C{^{1}H} NMR (101 MHz, $CDCl_3$) δ 161.8, 134.9, 129.8, 129.2, 127.1, 81.8, 63.9, 62.6, 41.9, 39.7, 38.4, 12.3.

IR ν 1728, 1452, 1435, 1356, 1275, 1247, 1172, 951 cm^{-1}.

HRMS (ESI) m/z = $[M + Na]^+$ Calcd $C_{14}H_{20}N_2NaO_5S^+$ 351.0985. Found 351.0963 (6.3 ppm error).

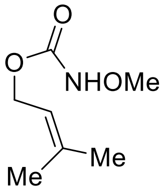

3-methylbut-2-en-1-yl methoxycarbamate

Compound 35: Previously characterized in *J. Org. Chem.* **2024**, *89*, 15352–15357.

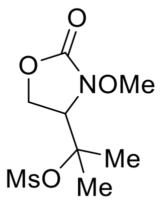

2-(3-methoxy-2-oxooxazolidin-4-yl)propan-2-yl methanesulfonate

Compound 36: Synthesized using **General Procedure C** on a 0.3 mmol scale; Purified using a gradient of 0 to 100% EtOAc/hexanes on silica gel; (light yellow oil, 0.028 g, 0.110 mmol, 37% yield).

^{1}H NMR (400 MHz, $CDCl_3$) δ 4.37 (t, *J* = 8.3 Hz, 1H), 4.23 (t, *J* = 7.8 Hz, 1H), 4.20 – 4.12 (m, 1H), 3.87 (s, 3H), 3.04 (s, 3H), 1.75 (s, 3H), 1.69 (s, 3H).

^{13}C{^{1}H} NMR (101 MHz, $CDCl_3$) δ 158.6, 90.3, 63.6, 63.1, 63.0, 40.8, 24.8, 23.1.

IR ν 1778, 1329, 1176, 1080, 891 cm^{-1}.

HRMS (ESI) m/z = $[M + Na]^+$ Calcd $C_8H_{15}NNaO_6S^+$ 276.0512. Found 276.0493 (6.9 ppm error).

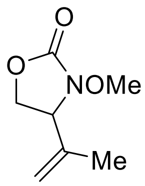

3-methoxy-4-(prop-1-en-2-yl)oxazolidin-2-one

Compound S1: Synthesized using **General Procedure C** on a 0.3 mmol scale; Purified using a gradient of 0 to 100% EtOAc/hexanes on silica gel; (light yellow oil, 0.010 g, 0.064 mmol, 21% yield).

^{1}H NMR (400 MHz, $CDCl_3$) δ 5.16 (s, 1H), 5.11 (p, *J* = 1.4 Hz, 1H), 4.39 – 4.28 (m, 2H), 4.02 (t, *J* = 7.6 Hz, 1H), 3.80 (s, 3H), 1.82 (t, *J* = 1.2 Hz, 3H).

^{13}C{^{1}H} NMR (101 MHz, $CDCl_3$) δ 159.0, 139.3, 118.0, 65.0, 64.4, 63.7, 16.9.

IR ν 1778, 1331, 1275, 1176, 1079, 897 cm^{-1}.

HRMS (ESI) m/z = $[M + Na]^+$ Calcd $C_7H_{11}NNaO_3^+$ 180.0631. Found 180.0644 (7.2 ppm error).

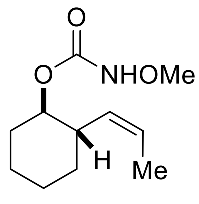

(1*R**,2*S**)-2-((*Z*)-prop-1-en-1-yl)cyclohexyl methoxycarbamate

Compound 37: Previously characterized in *J. Org. Chem.* **2024**, *89*, 15352–15357.

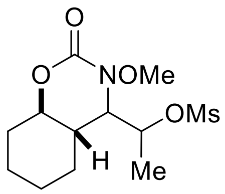

1-((4a*S**,8a*R**)-3-methoxy-2-oxooctahydro-2*H*-benzo[*e*][1,3]oxazin-4-yl)ethyl methanesulfonate

Compound 38: Synthesized using **General Procedure C** on a 0.3 mmol scale; Purified using a gradient of 0 to 100% EtOAc/hexanes on silica gel; Single diastereomer but full relative stereochemistry remains unassigned; (light yellow oil, 0.085 g, 0.277 mmol, 92% yield).

^{1}H NMR (400 MHz, $CDCl_3$) δ 5.24 (qd, *J* = 6.6, 3.9 Hz, 1H), 3.88 (dd, *J* = 8.1, 3.9 Hz, 1H), 3.83 (td, *J* = 10.9, 4.3 Hz, 1H), 3.72 (s, 3H), 3.03 (s, 3H), 2.20 – 2.07 (m, 2H), 1.84 (qd, *J* = 7.0, 3.6 Hz, 2H), 1.75 – 1.68 (m, 1H), 1.48 – 1.39 (m, 1H), 1.37 (d, *J* = 6.6 Hz, 3H), 1.32 – 1.20 (m, 3H).

^{13}C{^{1}H} NMR (101 MHz, $CDCl_3$) δ 154.6, 77.1, 76.3, 63.5, 61.8, 39.6, 38.4, 30.9, 30.6, 24.5, 23.7, 15.3.

IR ν 1720, 1354, 1174, 942, 922 cm^{-1}.

HRMS (ESI) m/z = $[M + Na]^+$ Calcd $C_{12}H_{21}NNaO_6S^+$ 330.0982. Found 330.0997 (4.5 ppm error).

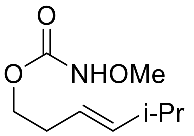

(*E*)-5-methylhex-3-en-1-yl methoxycarbamate

Compound 39: Synthesized using the procedure provided in *J. Org. Chem.* **2024**, *89,* 15352–15357 on a 3.75 mmol scale. Purified using a gradient of 0 to 50% EtOAc/hexanes on silica gel; (colorless oil, 0.483 g, 2.58 mmol, 69% yield).

^{1}H NMR (400 MHz, $CDCl_3$) δ 7.46 (br s, 1H), 5.48 (ddd, *J* = 15.4, 6.6, 1.2 Hz, 1H), 5.37 – 5.25 (m, 1H), 4.15 (t, *J* = 6.9 Hz, 2H), 3.72 (s, 3H), 2.36 – 2.29 (m, 2H), 2.23 (dp, *J* = 14.0, 7.0 Hz, 1H), 0.95 (d, *J* = 6.9 Hz, 6H).

^{13}C{^{1}H} NMR (101 MHz, $CDCl_3$) δ 157.8, 141.0, 121.7, 65.6, 64.7, 32.2, 31.1, 22.5.

IR ν 1728, 1466, 1120, 967 cm^{-1}.

HRMS (ESI) m/z = $[M + Na]^+$ Calcd $C_9H_{17}NNaO_3^+$ 210.1101. Found 210.1102 (0.5 ppm error).

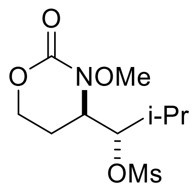

(*S**)-1-((*R**)-3-methoxy-2-oxo-1,3-oxazinan-4-yl)-2-methylpropyl methanesulfonate

Compound 40: Synthesized using **General Procedure C** on a 0.3 mmol scale; Purified using a gradient of 0 to 100% EtOAc/hexanes on silica gel; Single diastereomer; (white solid, 0.057 g, 0.202 mmol, 67% yield).

^{1}H NMR (600 MHz, $CDCl_3$) δ 4.90 – 4.85 (m, 1H), 4.28 (tt, *J* = 7.0, 3.7 Hz, 1H), 4.21 – 4.10 (m, 1H), 4.10 – 3.97 (m, 1H), 3.82 (s, 3H), 3.08 (s, 3H), 2.33 – 2.22 (m, 1H), 2.15 (ddt, *J* = 14.9, 7.2, 3.9 Hz, 1H), 2.04 – 1.90 (m, 1H), 1.13 (d, *J* = 6.8 Hz, 3H), 1.01 (d, *J* = 6.9 Hz, 3H).

^{13}C{^{1}H} NMR (101 MHz, $CDCl_3$) δ 153.7, 84.7, 64.1, 61.9, 57.6, 39.4, 30.1, 23.1, 19.8, 19.0.

IR ν 1721, 1339, 1171, 1108, 917 cm^{-1}.

HRMS (ESI) m/z = $[M + Na]^+$ Calcd $C_{10}H_{19}NNaO_6S^+$ 304.0825. Found 304.0806 (6.3 ppm error).

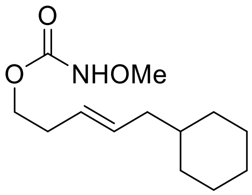

(*E*)-5-cyclohexylpent-3-en-1-yl methoxycarbamate

Compound 41: Synthesized using the procedure provided in *J. Org. Chem.* **2024**, *89,* 15352–15357 on a 4.05 mmol scale. Purified using a gradient of 0 to 50% EtOAc/hexanes on silica gel; (colorless oil, 0.433 g, 1.79 mmol, 44% yield).

^{1}H NMR (400 MHz, $CDCl_3$) δ 7.44 (br s, 1H), 5.55 – 5.43 (m, 1H), 5.38 – 5.26 (m, 1H), 4.15 (t, *J* = 6.9 Hz, 2H), 3.72 (s, 3H), 2.34 (qd, *J* = 6.7, 1.1 Hz, 2H), 1.87 (td, *J* = 6.9, 1.1 Hz, 2H), 1.73 – 1.62 (m, 5H), 1.26 – 1.11 (m, 4H), 0.93 – 0.78 (m, 2H).

^{13}C{^{1}H} NMR (101 MHz, $CDCl_3$) δ 157.7, 132.4, 125.7, 65.6, 64.7, 40.7, 38.0, 33.1, 32.3, 26.6, 26.4.

IR ν 2920, 1723, 1448, 1244, 1118, 969 cm^{-1}.

HRMS (ESI) m/z = $[M + Na]^+$ Calcd $C_{13}H_{23}NNaO_3^+$ 264.1570. Found 264.1547 (8.7 ppm error).

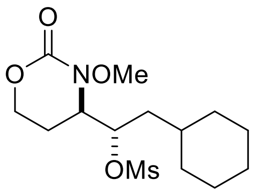

(*S**)-2-cyclohexyl-1-((*R**)-3-methoxy-2-oxo-1,3-oxazinan-4-yl)ethyl methanesulfonate

Compound 42: Synthesized using **General Procedure C** on a 0.3 mmol scale; Purified using a gradient of 0 to 100% EtOAc/hexanes on silica gel; Single diastereomer; (colorless oil, 0.080 g, 0.239 mmol, 80% yield).

^{1}H NMR (400 MHz, $CDCl_3$) δ 5.22 (td, *J* = 6.9, 2.0 Hz, 1H), 4.25 (dd, *J* = 6.5, 4.1 Hz, 1H), 4.12 (ddd, *J* = 10.9, 8.3, 3.6 Hz, 1H), 3.88 (ddd, *J* = 8.3, 6.3, 2.0 Hz, 1H), 3.82 (s, 3H), 3.05 (s, 3H), 2.32 – 2.21 (m, 1H), 2.21 – 2.10 (m, 1H), 1.85 – 1.58 (m, 6H), 1.46 – 1.33 (m, 2H), 1.29 – 1.10 (m, 3H), 1.02 (ddt, *J* = 10.7, 7.1, 2.3 Hz, 1H), 0.98 – 0.86 (m, 1H).

^{13}C{^{1}H} NMR (101 MHz, $CDCl_3$) δ 153.9, 77.7, 64.1, 62.1, 58.9, 39.4, 39.3, 33.8, 33.3, 33.2, 26.3, 26.0, 25.9, 22.9.

IR ν 1723, 1343, 1171, 1105, 908 cm^{-1}.

HRMS (ESI) m/z = $[M + Na]^+$ Calcd $C_{14}H_{25}NNaO_6S^+$ 358.1295. Found 358.1268 (7.5 ppm error).

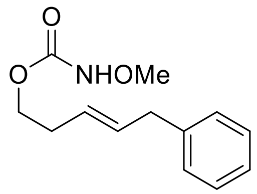

(*E*)-5-phenylpent-3-en-1-yl methoxycarbamate

Compound 43: Synthesized using the procedure provided in *J. Org. Chem.* **2024**, *89,* 15352–15357 on a 6.16 mmol scale. Purified using a gradient of 0 to 50% EtOAc/hexanes on silica gel; (colorless oil, 0.699 g, 2.97 mmol, 48% yield).

^{1}H NMR (400 MHz, $CDCl_3$) δ 7.38 (broad s, 1H), 7.32 – 7.27 (m, 2H), 7.22 – 7.16 (m, 3H), 5.70 (dtt, *J* = 15.1, 6.8, 1.3 Hz, 1H), 5.52 – 5.43 (m, 1H), 4.19 (t, *J* = 6.8 Hz, 2H), 3.71 (s, 3H), 3.35 (d, *J* = 6.7 Hz, 2H), 2.40 (qd, *J* = 6.8, 1.3 Hz, 2H).

^{13}C{^{1}H} NMR (101 MHz, $CDCl_3$) δ 157.7, 140.5, 132.3, 128.6, 128.5, 126.5, 126.1, 65.3, 64.7, 39.1, 32.2.

IR ν 1721, 1454, 1339, 1253, 1118, 969 cm^{-1}.

HRMS (ESI) m/z = $[M + Na]^+$ Calcd $C_{13}H_{17}NNaO_3^+$ 258.1101. Found 258.1107 (2.3 ppm error).

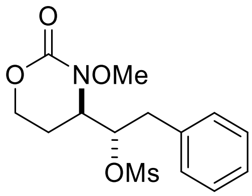

(S*)-1-((R*)-3-methoxy-2-oxo-1,3-oxazinan-4-yl)-2-phenylethyl methanesulfonate

Compound 44: Synthesized using **General Procedure C** on a 0.3 mmol scale; Purified using a gradient of 0 to 100% EtOAc/hexanes on silica gel; Single diastereomer; (light yellow oil, 0.068 g, 0.206 mmol, 68% yield).

^{1}H NMR (400 MHz, $CDCl_3$) δ 7.39 – 7.20 (m, 5H), 5.33 (ddd, J = 8.8, 6.8, 2.0 Hz, 1H), 4.29 (ddd, J = 10.9, 6.8, 3.9 Hz, 1H), 4.10 (ddd, J = 11.3, 8.1, 3.6 Hz, 1H), 3.79 (ddd, J = 8.1, 5.8, 2.0 Hz, 1H), 3.71 (s, 3H), 3.28 (dd, J = 14.0, 6.9 Hz, 1H), 2.93 (dd, J = 14.0, 8.7 Hz, 1H), 2.82 (s, 3H), 2.35 (dddd, J = 14.1, 8.1, 5.9, 4.0 Hz, 1H), 2.24 – 2.14 (m, 1H).

^{13}C{^{1}H} NMR (101 MHz, $CDCl_3$) δ 154.2, 135.1, 129.24, 129.22, 127.7, 80.1, 63.9, 62.0, 57.8, 38.9, 38.4, 22.9.

IR ν 1721, 1409, 1348, 1213, 1173, 907 cm^{-1}.

HRMS (ESI) m/z = $[M + Na]^+$ Calcd $C_{14}H_{19}NNaO_6S^+$ 352.0825. Found 352.0832 (2 ppm error).

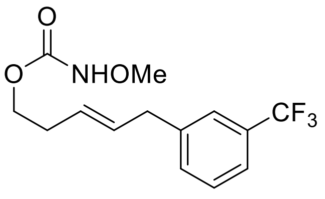

(E)-5-(3-(trifluoromethyl)phenyl)pent-3-en-1-yl methoxycarbamate

Compound 45: Synthesized using the procedure provided in *J. Org. Chem.* **2024**, *89,* 15352–15357 on a 4.77 mmol scale. Purified using a gradient of 0 to 50% EtOAc/hexanes on silica gel; (colorless oil, 0.450 g, 1.48 mmol, 31% yield).

^{1}H NMR (400 MHz, $CDCl_3$) δ 7.55 – 7.31 (m, 5H), 5.67 (dtt, J = 14.8, 6.6, 1.3 Hz, 1H), 5.51 (dtt, J = 15.2, 6.7, 1.4 Hz, 1H), 4.20 (t, J = 6.7 Hz, 2H), 3.70 (s, 3H), 3.40 (d, J = 6.7 Hz, 2H), 2.41 (dddd, J = 8.0, 6.8, 5.6, 1.2 Hz, 2H).

^{13}C{^{1}H} NMR (101 MHz, $CDCl_3$) δ 157.6, 141.4, 132.0, 131.2, 130.8 (q, J = 32.0 Hz), 128.9, 127.6, 125.7 (q, J = 273 Hz), 125.41 – 125.23 (m), 123.59 – 122.73 (m), 65.2, 64.7, 38.8, 32.2.

^{19}F{^{1}H} NMR (377 MHz, $CDCl_3$) δ -62.5.

IR ν 1709, 1329, 1249, 1119, 909 cm^{-1}.

HRMS (ESI) m/z = $[M + Na]^+$ Calcd $C_{14}H_{16}F_3NNaO_3^+$ 326.0974. Found 326.0985 (3.4 ppm error).

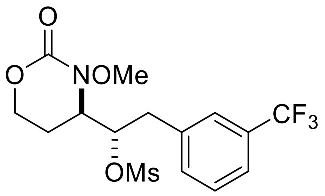

(S*)-1-((R*)-3-methoxy-2-oxo-1,3-oxazinan-4-yl)-2-(3-(trifluoromethyl)phenyl)ethyl methanesulfonate

Compound 46: Synthesized using **General Procedure C** on a 0.3 mmol scale; Purified using a gradient of 0 to 100% EtOAc/hexanes on silica gel; Single diastereomer; (colorless oil, 0.067 g, 0.169 mmol, 56% yield).

^{1}H NMR (600 MHz, $CDCl_3$) δ 7.59 – 7.45 (m, 4H), 5.36 – 5.30 (m, 1H), 4.29 (ddd, J = 10.8, 6.4, 4.1 Hz, 1H), 4.12 (ddd, J = 11.5, 8.5, 3.5 Hz, 1H), 3.87 – 3.79 (m, 1H), 3.73 (s, 3H), 3.31 (dd, J = 14.2, 7.0 Hz, 1H), 3.00 (dd, J = 14.2, 8.1 Hz, 1H), 2.88 (s, 3H), 2.34 (qd, J = 9.2, 6.0 Hz, 1H), 2.26 – 2.14 (m, 1H).

^{13}C{^{1}H} NMR (101 MHz, $CDCl_3$) δ 154.1, 136.1, 132.7, 131.5 (q, J = 32.4 Hz), 129.7, 126.0 (q, J = 3.8 Hz), 124.6 (q, J = 3.8 Hz), 122.5 (q, J = 274 Hz), 79.3, 63.8, 62.0, 58.0, 39.0, 38.1, 23.0.

^{19}F{^{1}H} NMR (377 MHz, $CDCl_3$) δ -62.6.

IR ν 1723, 1329, 1172, 1120, 1073, 910 cm^{-1}.

HRMS (ESI) m/z = $[M + Na]^+$ Calcd $C_{15}H_{18}F_3NNaO_6S^+$ 420.0699. Found 420.0699 (0 ppm error).

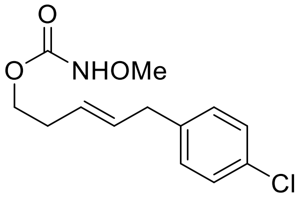

(E)-5-(4-chlorophenyl)pent-3-en-1-yl methoxycarbamate

Compound 47: Synthesized using the procedure provided in *J. Org. Chem.* **2024**, *89,* 15352–15357 on a 6.61 mmol scale. Purified using a gradient of 0 to 50% EtOAc/hexanes on silica gel; (amorphous white solid, 0.673 g, 2.5 mmol, 38% yield).

^{1}H NMR (400 MHz, $CDCl_3$) δ 7.36 (broad s, 1H), 7.25 (dd, J = 8.3, 2.0 Hz, 2H), 7.13 – 7.06 (m, 2H), 5.70 – 5.59 (m, 1H), 5.52 – 5.41 (m, 1H), 4.19 (t, J = 6.7 Hz, 2H), 3.71 (s, 3H), 3.30 (d, J = 6.7 Hz, 2H), 2.39 (dddd, J = 8.0, 6.8, 5.6, 1.3 Hz, 2H).

^{13}C{^{1}H} NMR (101 MHz, $CDCl_3$) δ 157.6, 138.9, 131.9, 131.7, 129.9, 128.6, 127.0, 65.2, 64.7, 38.4, 32.2.

IR ν 1716, 1489, 1330, 1247, 1119, 968 cm^{-1}.

HRMS (ESI) m/z = $[M + Na]^+$ Calcd $C_{13}H_{16}ClNNaO_3^+$ 292.0711. Found 292.0714 (1.0 ppm error).

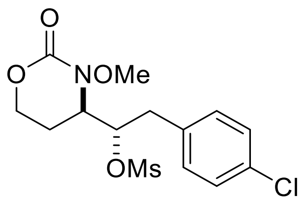

(S*)-2-(4-chlorophenyl)-1-((R*)-3-methoxy-2-oxo-1,3-oxazinan-4-yl)ethyl methanesulfonate

Compound 48: Synthesized using **General Procedure C** on a 0.3 mmol scale; Purified using a gradient of 0 to 100% EtOAc/hexanes on silica gel; Single diastereomer; (colorless oil, 0.077 g, 0.212 mmol, 70% yield).

^{1}H NMR (600 MHz, $CDCl_3$) δ 7.33 (d, J = 8.0 Hz, 2H), 7.18 (d, J = 8.0 Hz, 2H), 5.28 (ddd, J = 8.8, 6.4, 2.0 Hz, 1H), 4.28 (ddd, J = 10.7, 6.3, 3.9 Hz, 1H), 4.10 (ddd, J = 11.3, 8.6, 3.5 Hz, 1H), 3.79 (ddd, J = 8.3, 6.0, 2.1 Hz, 1H), 3.72 (s, 3H), 3.25 (dd, J = 14.1, 6.7 Hz, 1H), 2.99 – 2.82 (m, 4H), 2.32 (ddt, J = 14.4, 9.0, 5.0 Hz, 1H), 2.17 (tdt, J = 9.0, 6.5, 3.4 Hz, 1H).

^{13}C{^{1}H} NMR (101 MHz, $CDCl_3$) δ 154.1, 133.7, 133.5, 130.5, 129.4, 79.5, 63.9, 62.1, 57.8, 39.0, 37.8, 22.9.

IR ν 1718, 1346, 1282, 1171, 906 cm^{-1}.

HRMS (ESI) m/z = $[M + Na]^+$ Calcd $C_{14}H_{18}ClNNaO_6S^+$ 386.0436. Found 386.0435 (0.3 ppm error).

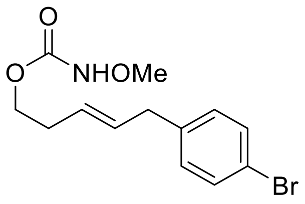

(E)-5-(4-bromophenyl)pent-3-en-1-yl methoxycarbamate

Compound 49: Synthesized using the procedure provided in *J. Org. Chem.* **2024**, *89,* 15352–15357 on a 5.81 mmol scale. Purified using a gradient of 0 to 50% EtOAc/hexanes on silica gel; (white solid, 0.612 g, 1.95 mmol, 33% yield).

^{1}H NMR (400 MHz, $CDCl_3$) δ 7.42 – 7.36 (m, 3H), 7.06 – 7.01 (m, 2H), 5.70 – 5.58 (m, 1H), 5.52 – 5.39 (m, 1H), 4.18 (t, J = 6.7 Hz, 2H), 3.70 (s, 3H), 3.28 (d, J = 6.6 Hz, 2H), 2.39 (qd, J = 6.7, 1.2 Hz, 2H).

^{13}C{^{1}H} NMR (101 MHz, $CDCl_3$) δ 157.6, 139.4, 131.6, 131.5, 130.3, 127.1, 119.9, 65.2, 64.7, 38.4, 32.2.

IR ν 1721, 1486, 1246, 1116, 1069, 1010, 969 cm^{-1}.

HRMS (ESI) m/z = $[M + Na]^+$ Calcd $C_{13}H_{16}BrNNaO_3^+$ 336.0206. Found 336.0234 (8.3 ppm error).

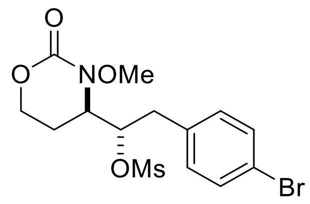

(*S**)-2-(4-bromophenyl)-1-((*R**)-3-methoxy-2-oxo-1,3-oxazinan-4-yl)ethyl methanesulfonate

Compound 50: Synthesized using **General Procedure C** on a 0.3 mmol scale; Purified using a gradient of 0 to 100% EtOAc/hexanes on silica gel; Single diastereomer; (light yellow oil, 0.085 g, 0.208 mmol, 69% yield).

^{1}H NMR (400 MHz, $CDCl_3$) δ 7.54 – 7.41 (m, 2H), 7.18 – 7.03 (m, 2H), 5.28 (ddd, *J* = 8.6, 6.6, 2.0 Hz, 1H), 4.27 (ddd, *J* = 10.6, 6.3, 4.0 Hz, 1H), 4.18 – 4.01 (m, 1H), 3.78 (ddd, *J* = 8.3, 6.1, 2.0 Hz, 1H), 3.71 (s, 3H), 3.23 (dd, *J* = 14.1, 6.6 Hz, 1H), 2.95 – 2.80 (m, 4H), 2.39 – 2.27 (m, 1H), 2.24 – 2.14 (m, 1H).

^{13}C{^{1}H} NMR (101 MHz, $CDCl_3$) δ 154.1, 134.0, 132.3, 130.9, 121.7, 79.4, 63.9, 62.1, 57.8, 39.0, 37.8, 22.9.

IR ν 1716, 1489, 1407, 1393, 1267, 1171, 904 cm^{-1}.

HRMS (ESI) m/z = $[M + Na]^+$ Calcd $C_{14}H_{18}BrNNaO_6S^+$ 429.9930. Found 429.9900 (6.9 ppm error).

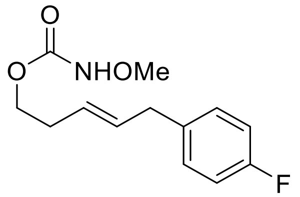

(*E*)-5-(4-fluorophenyl)pent-3-en-1-yl methoxycarbamate

Compound 51: Synthesized using the procedure provided in *J. Org. Chem.* **2024***, 89,* 15352–15357 on a 6.60 mmol scale. Purified using a gradient of 0 to 50% EtOAc/hexanes on silica gel; (amorphous white solid, 0.501 g, 1.98 mmol, 30% yield).

^{1}H NMR (400 MHz, $CDCl_3$) δ 7.45 (broad s, 1H), 7.15 – 7.06 (m, 2H), 7.01 – 6.92 (m, 2H), 5.65 (dtt, *J* = 14.8, 6.7, 1.3 Hz, 1H), 5.52 – 5.40 (m, 1H), 4.18 (t, *J* = 6.8 Hz, 2H), 3.70 (s, 3H), 3.30 (d, *J* = 6.4 Hz, 2H), 2.39 (dddd, *J* = 7.9, 6.8, 5.7, 1.2 Hz, 2H).

^{13}C{^{1}H} NMR (101 MHz, $CDCl_3$) δ 161.5 (d, *J* = 243.7 Hz), 157.7, 136.0 (d, *J* = 3.1 Hz), 132.1, 129.9 (d, *J* = 7.8 Hz), 126.7, 115.2 (d, *J* = 21.1 Hz), 65.2, 64.7, 38.2, 32.1.

^{19}F{^{1}H} NMR (377 MHz, $CDCl_3$) δ -117.5.

IR ν 1721, 1508, 1219, 1119, 969 cm^{-1}.

HRMS (ESI) m/z = $[M + Na]^+$ Calcd $C_{13}H_{16}FNNaO_3^+$ 276.1006. Found 276.0988 (6.5 ppm error).

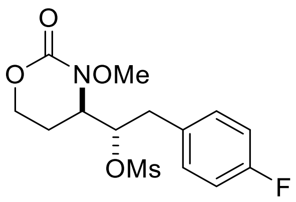

(*S**)-2-(4-fluorophenyl)-1-((*R**)-3-methoxy-2-oxo-1,3-oxazinan-4-yl)ethyl methanesulfonate

Compound 52: Synthesized using **General Procedure C** on a 0.3 mmol scale; Purified using a gradient of 0 to 100% EtOAc/hexanes on silica gel; Single diastereomer; (light yellow oil, 0.074 g, 0.213 mmol, 71% yield).

^{1}H NMR (400 MHz, $CDCl_3$) δ 7.21 (dd, *J* = 8.3, 5.2 Hz, 2H), 7.04 (t, *J* = 8.4 Hz, 2H), 5.37 – 5.15 (m, 1H), 4.28 (ddd, *J* = 10.8, 6.4, 3.9 Hz, 1H), 4.10 (ddd, *J* = 11.5, 8.3, 3.6 Hz, 1H), 3.79 (ddd, *J* = 8.3, 6.0, 2.0 Hz, 1H), 3.71 (s, 3H), 3.23 (dd, *J* = 14.1, 6.8 Hz, 1H), 3.00 – 2.80 (m, 4H), 2.32 (ddt, *J* = 14.0, 9.3, 4.2 Hz, 1H), 2.26 – 2.09 (m, 1H).

^{13}C{^{1}H} NMR (101 MHz, $CDCl_3$) δ 162.2 (d, *J* = 246.7 Hz), 154.1, 131.83 – 129.47 (m, 2C), 116.15 (d, *J* = 21.4 Hz), 79.8, 63.9, 62.0, 57.8, 39.0, 37.6, 22.9.

^{19}F{^{1}H} NMR (377 MHz, $CDCl_3$) δ -114.3.

IR ν 1717, 1511, 1343, 1172, 961, 904 cm^{-1}.

HRMS (ESI) m/z = $[M + Na]^+$ Calcd $C_{14}H_{18}FNNaO_6S^+$ 370.0731. Found 370.0717 (3.8 ppm error).

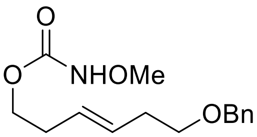

(*E*)-6-(benzyloxy)hex-3-en-1-yl methoxycarbamate

Compound 53: Previously characterized in *J. Org. Chem.* **2024**, *89*, 15352–15357.

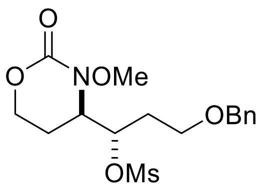

(*S**)-3-(benzyloxy)-1-((*R**)-3-methoxy-2-oxo-1,3-oxazinan-4-yl)propyl methanesulfonate

Compound 54: Synthesized using **General Procedure C** on a 0.3 mmol scale; Purified using a gradient of 0 to 100% EtOAc/hexanes on silica gel; Single diastereomer; (light yellow oil which solidifies upon standing, 0.077 g, 0.206 mmol, 68% yield).

^{1}H NMR (600 MHz, $CDCl_3$) δ 7.39 – 7.27 (m, 5H), 5.24 (td, *J* = 6.8, 2.1 Hz, 1H), 4.53 (d, *J* = 11.7 Hz, 1H), 4.48 (d, *J* = 11.7 Hz, 1H), 4.27 – 4.21 (m, 1H), 4.13 – 4.03 (m, 2H), 3.75 (s, 3H), 3.66 – 3.57 (m, 2H), 3.05 (s, 3H), 2.24 (dddd, *J* = 13.6, 9.2, 6.8, 4.1 Hz, 1H), 2.18 (dddd, *J* = 10.8, 8.5, 5.4, 2.9 Hz, 1H), 2.12 (ddt, *J* = 15.3, 6.7, 4.5 Hz, 1H), 1.94 (ddt, *J* = 14.7, 8.2, 6.3 Hz, 1H).

^{13}C{^{1}H} NMR (101 MHz, $CDCl_3$) δ 153.8, 137.8, 128.6, 128.0, 127.9, 77.9, 73.4, 65.8, 64.1, 61.8, 58.8, 39.0, 32.3, 23.0.

IR ν 1719, 1344, 1172, 1100, 1078, 940, 910, 876 cm^{-1}.

HRMS (ESI) m/z = $[M + Na]^+$ Calcd $C_{16}H_{23}NNaO_7S^+$ 396.1087. Found 396.1073 (3.5 ppm error).

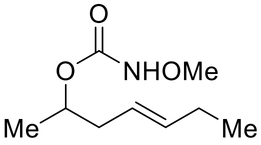

(*E*)-hept-4-en-2-yl methoxycarbamate

Compound 55: Synthesized using the procedure provided in *J. Org. Chem.* **2024***, 89,* 15352–15357 on a 6.44 mmol scale. Purified using a gradient of 0 to 50% EtOAc/hexanes on silica gel; (light yellow oil, 0.978 g, 5.22 mmol, 81% yield).

^{1}H NMR (400 MHz, $CDCl_3$) δ 7.33 (br s, 1H), 5.53 (dtd, *J* = 15.3, 6.3, 1.4 Hz, 1H), 5.43 – 5.27 (m, 1H), 4.89 (qd, *J* = 6.3, 1.7 Hz, 1H), 3.72 (s, 3H), 2.37 – 2.16 (m, 2H), 2.00 (qdt, *J* = 7.4, 6.4, 1.2 Hz, 2H), 1.24 (dd, *J* = 6.2, 1.5 Hz, 3H), 1.03 – 0.90 (m, 3H).

^{13}C{^{1}H} NMR (101 MHz, $CDCl_3$) δ 157.4, 135.9, 123.6, 72.7, 64.7, 39.3, 25.7, 19.6, 13.8.

IR ν 1712, 1461, 1381, 1256, 1115, 967 cm^{-1}.

HRMS (ESI) m/z = $[M + Na]^+$ Calcd $C_9H_{17}NNaO_3^+$ 210.1101. Found 210.1090 (5.2 ppm error).

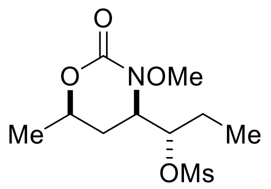

(S^*)-1-((4R^*,6R^*)-3-methoxy-6-methyl-2-oxo-1,3-oxazinan-4-yl)propyl methanesulfonate

Compound 56: Synthesized using **General Procedure C** on a 0.3 mmol scale; Purified using a gradient of 0 to 100% EtOAc/hexanes on silica gel; Single diastereomer; (light yellow oil which solidifies upon standing, 0.068 g, 0.242 mmol, 80% yield).

^{1}H NMR (400 MHz, $CDCl_3$) δ 5.03 (td, *J* = 7.0, 2.1 Hz, 1H), 4.32 (dqd, *J* = 12.5, 6.2, 2.3 Hz, 1H), 4.03 (ddd, *J* = 10.1, 8.1, 2.1 Hz, 1H), 3.76 (s, 3H), 3.07 (s, 3H), 2.17 – 1.95 (m, 2H), 1.87 (dp, *J* = 15.1, 7.6 Hz, 1H), 1.60 (dp, *J* = 14.4, 7.4 Hz, 1H), 1.37 (d, *J* = 6.1 Hz, 3H), 1.03 (t, *J* = 7.5 Hz, 3H).

^{13}C{^{1}H} NMR (101 MHz, $CDCl_3$) δ 153.6, 79.9, 71.3, 61.9, 57.6, 39.3, 29.7, 25.1, 20.2, 10.1.

IR ν 1717, 1346, 1172, 1111, 1095, 906 cm^{-1}.

HRMS (ESI) m/z = $[M + Na]^+$ Calcd $C_{10}H_{19}NNaO_6S^+$ 304.0825. Found 304.0832 (2.3 ppm error).

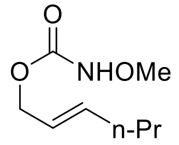

(E)-hex-2-en-1-yl methoxycarbamate

Compound 57: Previously characterized in *J. Org. Chem.* **2024**, *89*, 15352–15357.

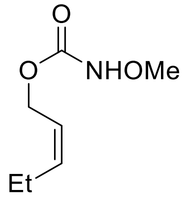

(Z)-pent-2-en-1-yl methoxycarbamate

Compound 58: Previously characterized in *J. Org. Chem.* **2024**, *89*, 15352–15357.

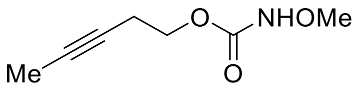

pent-3-yn-1-yl methoxycarbamate

Compound 59: Synthesized using the procedure provided in *J. Org. Chem.* **2024***, 89,* 15352–15357 on a 5 mmol scale; Purified using a gradient of 15 to 50% EtOAc/hexanes on silica gel; (colorless oil, 0.745 g, 4.74 mmol, 95% yield).

^{1}H NMR (600 MHz, $CDCl_3$) δ 7.39 (broad s, 1H), 4.21 (t, *J* = 6.9 Hz, 2H), 3.75 (s, 3H), 2.49 (tq, *J* = 7.0, 2.5 Hz, 2H), 1.78 (t, *J* = 2.6 Hz, 3H).

$^{13}C\{^{1}H\}$ NMR (101 MHz, $CDCl_3$) δ 157.3, 77.5, 74.5, 64.7, 64.1, 19.5, 3.5.

IR ν 1721, 1464, 1341, 1247, 1116, 933 cm^{-1}.

HRMS (ESI) m/z = $[M + Na]^+$ Calcd $C_7H_{11}NNaO_3^+$ 180.0631. Found 180.0648 (9.4 ppm error).

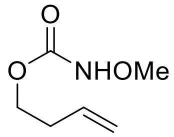

but-3-en-1-yl methoxycarbamate

Compound 60: Previously characterized in *J. Org. Chem*. **2024**, *89*, 15352–15357.

IV. Procedures for scale-up/applications and product characterization (Scheme 3)

Scale-up reaction

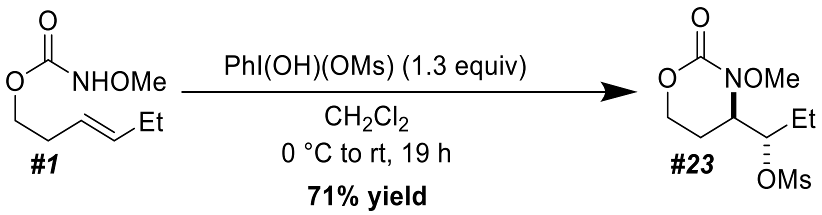

A 500 mL round-bottom flask equipped with a magnetic stir bar was charged with substrate **1** (2.0 g, 11.5 mmol, 1 equiv) and CH_2Cl_2 (200 mL). The flask was cooled to 0 °C using an ice-water bath. PhI(OH)(OMs) (4.75 g, 15.0 mmol, 1.3 equiv) was added in one bolus. The sides of the flask were rinsed with an additional 30 mL of CH_2Cl_2 (final reaction concentration = 0.05 M). With stirring, the reaction was warmed to room temperature over a period of 19 hours. Then, the contents were transferred to a separatory funnel with CH_2Cl_2 (100 mL). The organic layer was washed with one portion of saturated, aqueous $Na_2S_2O_3$ solution (100 mL). The organic layer was collected, dried with $MgSO_4$, filtered, and concentrated under reduced pressure. The resulting residue was purified by chromatography on silica gel (gradient of 0 to 100% EtOAc in hexanes) to give (*S**)-1-((*R**)-3-methoxy-2-oxo-1,3-oxazinan-4-yl)propyl methanesulfonate (product **23**) as a pale yellow solid (2.18 g, 8.16 mmol, 71% yield).

Azide displacement

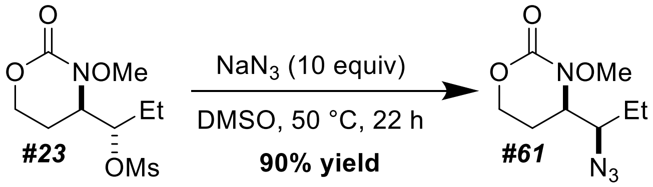

A 5 mL microwave vial equipped with a magnetic stir bar was charged with compound **23** (0.054 g, 0.2 mmol, 1 equiv), NaN_3 (0.131 g, 2 mmol, 10 equiv), and anhydrous DMSO (2 mL, final reaction concentration = 0.1 M). The vial was sealed and immersed into an oil-bath pre-heated to 50 °C. The reaction was stirred at this temperature for 22 hours. Then, the vial was removed from the oil bath, cooled to room temperature, and the seal was broken. The contents of the vial were transferred to a separatory funnel with EtOAc (40 mL). The organic layer was washed with brine (2 × 30 mL), collected, dried with $MgSO_4$, filtered, and concentrated in vacuo. The resulting residue was purified by chromatography on silica gel (gradient of 10 to 40% acetone/hexanes) to give **61** (light yellow oil, 0.0385 g, 0.180 mmol, 90% yield).

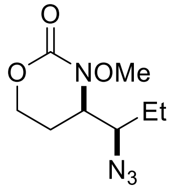

(*R**)-4-((*R**)-1-azidopropyl)-3-methoxy-1,3-oxazinan-2-one

Compound 61:

^{1}H NMR (400 MHz, $CDCl_3$) δ 4.21 (ddd, *J* = 11.1, 7.2, 3.9 Hz, 1H), 4.14 (ddd, *J* = 11.1, 7.4, 3.8 Hz, 1H), 3.98 (ddd, *J* = 7.5, 6.0, 3.8 Hz, 1H), 3.78 (s, 3H), 3.65 (dt, *J* = 10.7, 3.5 Hz, 1H), 2.17 (dtd, *J* = 14.7, 7.4, 3.8 Hz, 1H), 2.10 – 1.99 (m, 1H), 1.82 – 1.69 (m, 1H), 1.54 (ddq, *J* = 14.2, 10.7, 7.3 Hz, 1H), 1.11 (t, *J* = 7.4 Hz, 3H).

^{13}C{^{1}H} NMR (101 MHz, $CDCl_3$) δ 154.1, 65.1, 64.5, 62.1, 59.4, 24.5, 22.4, 11.5.

IR ν 2102, 1715, 1411, 1273, 1197, 1102 cm^{-1}.

HRMS (ESI) m/z = $[M + Na]^+$ Calcd $C_8H_{14}N_4NaO_3^+$ 237.0958. Found 237.0954 (1.7 ppm error).

Reduction

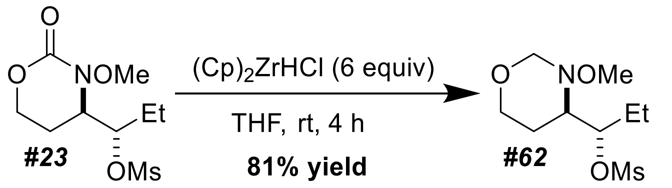

A 5 mL microwave vial equipped with a magnetic stir bar was charged with compound **23** (0.054 g, 0.2 mmol, 1 equiv), $(Cp)_2ZrHCl$ (0.309 g, 1.2 mmol, 6 equiv), and anhydrous THF (2 mL, final reaction concentration = 0.1 M). Vigorous effervescence was noted upon addition of THF. The vial was sealed, and the reaction was stirred at ambient temperature for 4 hours. Then, the seal was broken, and the contents of the vial were transferred to a separatory funnel with EtOAc (50 mL). The organic layer was washed with brine (30 mL), collected, dried with $MgSO_4$, filtered, and concentrated in vacuo. The resulting residue was purified by chromatography on silica gel (gradient of 10 to 50% EtOAc/hexanes) to give **62** (light yellow oil, 0.041 g, 0.162 mmol, 81% yield).

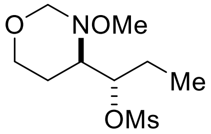

(*S**)-1-((*R**)-3-methoxy-1,3-oxazinan-4-yl)propyl methanesulfonate

Compound 62:

^1H NMR (500 MHz, $CDCl_3$) δ 4.99 – 4.85 (m, 1H), 4.78 (d, J = 10.1 Hz, 1H), 4.18 – 3.93 (m, 2H), 3.68 – 3.45 (m, 4H), 3.08 (s, 3H), 2.98 – 2.74 (m, 1H), 2.19 – 2.03 (m, 1H), 1.89 – 1.78 (m, 2H), 1.70 – 1.50 (m, 1H), 1.04 (t, J = 7.4 Hz, 3H). (*Note: temperature was 55 °C*)

$^{13}C\{^1H\}$ NMR (126 MHz, $CDCl_3$) δ 83.7, 82.8, 66.5, 63.8, 60.1, 39.1, 25.0, 23.4, 9.1. (*Note: temperature was 55 °C*)

IR ν 1333, 1168, 1079, 1039, 923 cm^{-1}.

HRMS (ESI) m/z = $[M + H]^+$ Calcd $C_9H_{20}NO_5S^+$ 254.1057. Found 254.1054 (1.2 ppm error).

V. Structural Reasoning

Compound 56

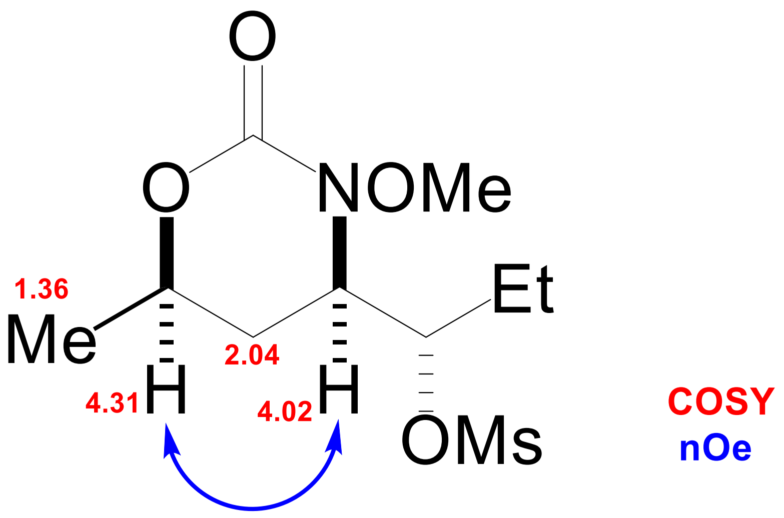

COSY, $CDCl_3$

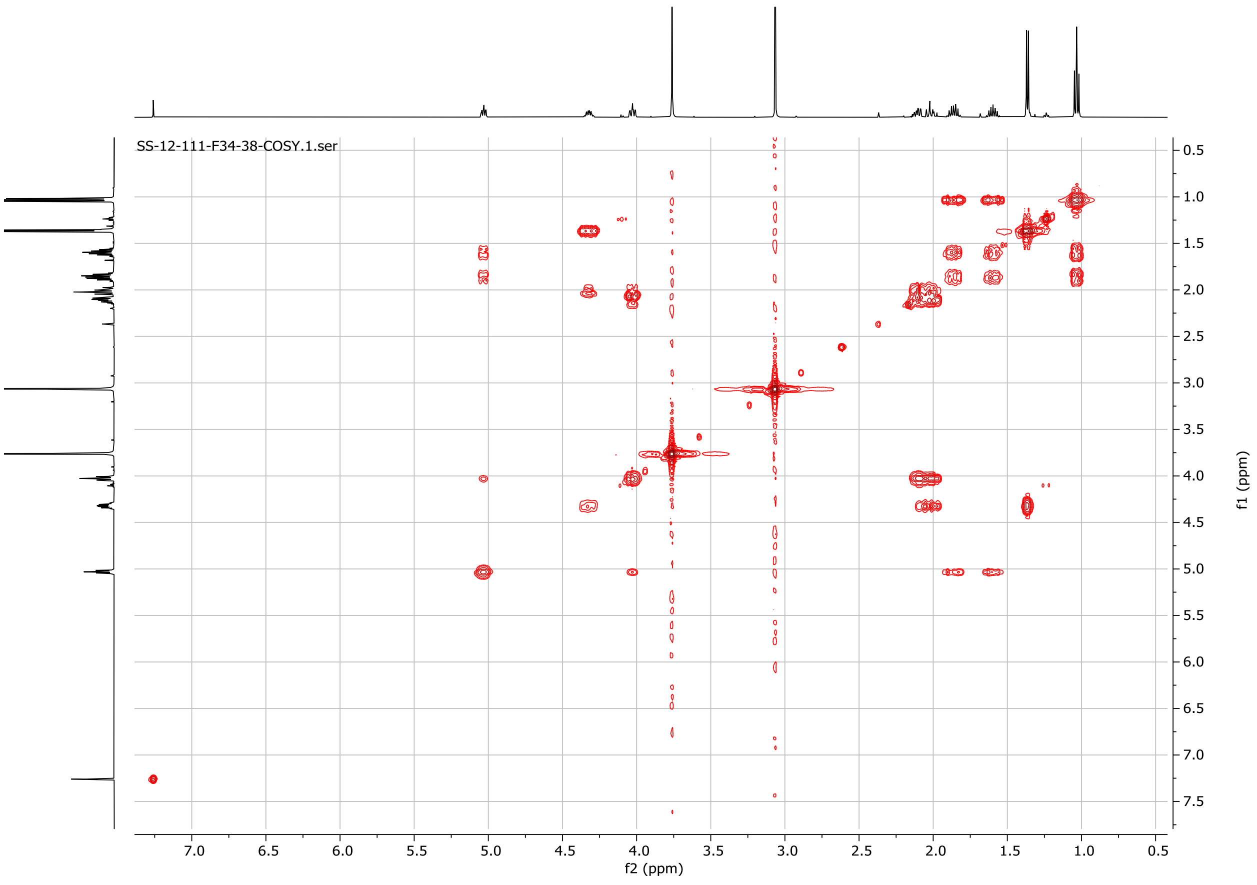

1-D nOe

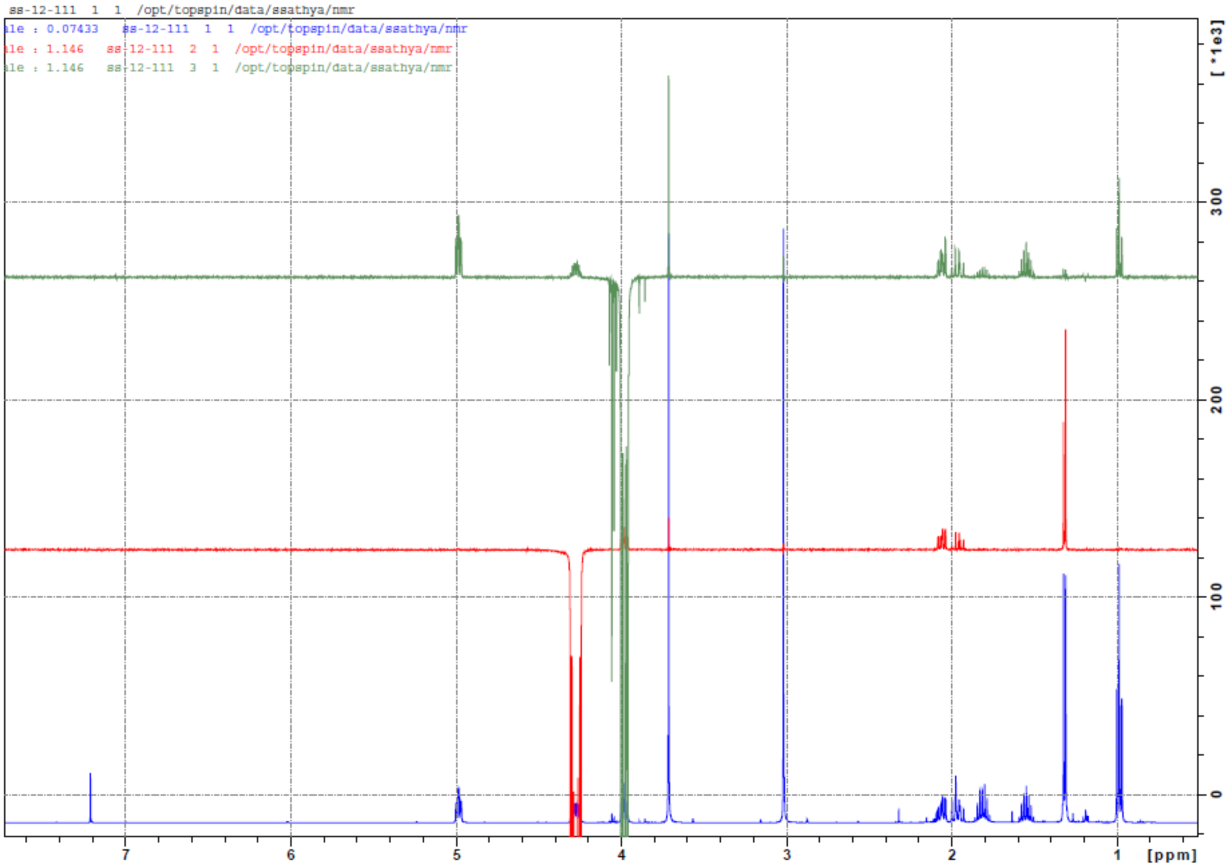
ss-12-111 1 1 /opt/topspin/data/ssathya/nmr
ale : 0.07433 ss-12-111 1 1 /opt/topspin/data/ssathya/nmr
ale : 1.146 ss-12-111 2 1 /opt/topspin/data/ssathya/nmr
ale : 1.146 ss-12-111 3 1 /opt/topspin/data/ssathya/nmr
[*1e3]
300
200
100
0
7
6
5
4
3
2
1
[ppm]

VI. X-Ray Crystallographic Data

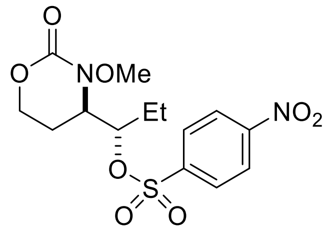

Compound 20 (CCDC 2391529)

Crystals grown by slow evaporation from ethanol.

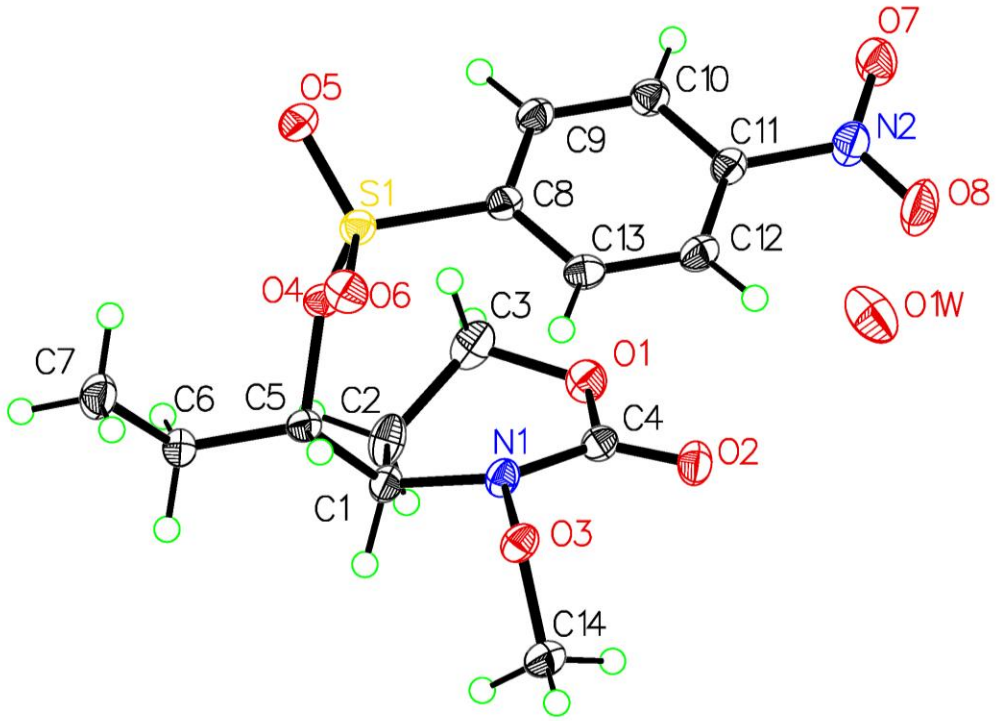

Labeled 50% probability ellipsoid plot of asymmetric unit of SS-12-69.

Single crystal X-ray diffraction data was collected on a Rigaku Synergy 4-circle diffractometer equipped with a Hypix photon-counting detector using Cu-Kα radiation from a microfocus source (Rigaku Americas, The Woodlands, TX). The crystal was cooled to the collection temperature under a stream of cold N_2 using a Cryostream 1000 cryostat (Oxford Cryosystems, Oxford, UK). Data was collected using shutterless scans with 0.5° frame widths and variable scanning rates. Data collection, unit cell determination, data reduction absorption correction, and scaling were done using Rigaku CrysAlisPro.[1]

The structure was solved by direct methods using SHELXS[2] and refined by full matrix least squares refinement against F^2 using SHELXL v.2019/3.[3] Olex2 was used as a graphical interface for model building and structure visualization.[4] Full occupancy non-hydrogen atoms were located from the difference map and refined anisotropically. Hydrogen atoms were placed in calculated positions, and their coordinates were allowed to refine while their thermal parameters were constrained to ride on the carrier atoms. A significant electron density peak with no connectivity to the main moiety was assigned as the partial occupancy oxygen atom of a water molecule of crystallization, and its occupancy and anisotropic thermal parameters were allowed to refine. The occupancy converged to 22.5(5)% which has been approximated as 25% in the formula to give an integer number of atoms per unit cell. The hydrogen atoms could not be located or inferred from neighboring atoms and are likely further disordered over multiple orientations in addition to the substitutional disorder of the entire molecule.

References

1. CrysAlisPro, version 1.171.43.124a, Rigaku Oxford Diffraction, Tokyo, Japan, 2024.
2. Sheldrick, G. M. SHELXS, v.2013-1, 2013.
3. Sheldrick, G. M. *SHELXT* – Integrated space-group and crystal-structure determination. *Acta Cryst. Sect. A: Found. Adv.* **2015**, *71*, 3-8.
4. Dolomanov, O.V.; Bourhis, L.J.; Gildea, R.J.; Howard, J.A.K.; Puschmann, H. *OLEX2:* A complete structure solution, refinement, and analysis program. *J. Appl. Cryst.* **2009**, *42*, 339-341.

Table S1. Crystal data and structure refinement for SS-12-69.

Identification code	s1	
Empirical formula	C14 H18.50 N2 O8.25 S	
Formula weight	378.87	
Temperature	100(2) K	
Wavelength	1.54178 Å	
Crystal system	Monoclinic	
Space group	$P2_1/c$	
Unit cell dimensions	a = 7.41513(7) Å	α= 90°.
	b = 16.76461(16) Å	β= 101.4851(9)°.
	c = 13.62560(11) Å	γ = 90°.
Volume	1659.91(3) $Å^3$	
Z	4	
Density (calculated)	1.516 Mg/m^3	
Absorption coefficient	2.192 mm^{-1}	
F(000)	794	
Crystal size	0.12 x 0.10 x 0.04 mm^3	
Theta range for data collection	4.233 to 80.145°.	
Index ranges	-9<=h<=9, -21<=k<=21, -14<=l<=17	
Reflections collected	36760	
Independent reflections	3618 [R(int) = 0.0410]	
Completeness to theta = 67.679°	100.0 %	
Absorption correction	Semi-empirical from equivalents	
Max. and min. transmission	1.00000 and 0.91574	
Refinement method	Full-matrix least-squares on F^2	
Data / restraints / parameters	3618 / 0 / 290	
Goodness-of-fit on F^2	1.077	
Final R indices [I>2sigma(I)]	R1 = 0.0312, wR2 = 0.0847	
R indices (all data)	R1 = 0.0333, wR2 = 0.0862	
Extinction coefficient	n/a	
Largest diff. peak and hole	0.295 and -0.401 e.$Å^{-3}$	

Table S2. Atomic coordinates (x 10^4) and equivalent isotropic displacement parameters (Å^{2}x 10^3) for SS-12-69. U(eq) is defined as one third of the trace of the orthogonalized U^{ij} tensor.

	x	y	z	U(eq)
S(1)	3833(1)	6564(1)	3783(1)	18(1)
O(1)	1174(1)	4892(1)	1155(1)	24(1)
O(1W)	-2768(8)	4010(3)	1730(4)	41(2)
O(2)	-1512(1)	5488(1)	1085(1)	24(1)
O(3)	-95(1)	6937(1)	1149(1)	20(1)
O(4)	4460(1)	6400(1)	2767(1)	18(1)
O(5)	5352(1)	6326(1)	4544(1)	25(1)
O(6)	3100(1)	7351(1)	3792(1)	24(1)
O(7)	-1798(1)	3534(1)	3997(1)	30(1)
O(8)	-3788(2)	4430(1)	3365(1)	44(1)
N(1)	1035(1)	6264(1)	1186(1)	19(1)
N(2)	-2210(2)	4207(1)	3677(1)	25(1)
C(1)	2876(2)	6441(1)	1010(1)	18(1)
C(2)	3740(2)	5660(1)	760(1)	26(1)
C(3)	3163(2)	4972(1)	1344(1)	28(1)
C(4)	132(2)	5550(1)	1127(1)	20(1)
C(5)	3952(2)	6929(1)	1880(1)	18(1)
C(6)	5728(2)	7272(1)	1667(1)	22(1)
C(7)	6736(2)	7803(1)	2508(1)	28(1)
C(8)	2035(2)	5867(1)	3750(1)	18(1)
C(9)	2487(2)	5081(1)	4026(1)	21(1)
C(10)	1082(2)	4530(1)	3994(1)	21(1)
C(11)	-718(2)	4787(1)	3682(1)	20(1)
C(12)	-1182(2)	5564(1)	3398(1)	21(1)
C(13)	223(2)	6116(1)	3434(1)	20(1)
C(14)	-971(2)	7107(1)	129(1)	24(1)

Table S3. Bond lengths [Å] and angles [°] for SS-12-69.

S(1)-O(5)	1.4272(9)
S(1)-O(6)	1.4288(9)
S(1)-O(4)	1.5709(9)
S(1)-C(8)	1.7662(13)
O(1)-C(4)	1.3436(16)
O(1)-C(3)	1.4520(17)
O(2)-C(4)	1.2134(16)
O(3)-N(1)	1.4002(13)
O(3)-C(14)	1.4412(15)
O(4)-C(5)	1.4860(14)
O(7)-N(2)	1.2261(16)
O(8)-N(2)	1.2212(16)
N(1)-C(4)	1.3664(16)
N(1)-C(1)	1.4633(15)
N(2)-C(11)	1.4717(16)
C(1)-C(2)	1.5264(17)
C(1)-C(5)	1.5288(16)
C(2)-C(3)	1.510(2)
C(5)-C(6)	1.5176(17)
C(6)-C(7)	1.5240(18)
C(8)-C(13)	1.3911(17)
C(8)-C(9)	1.3925(17)
C(9)-C(10)	1.3864(18)
C(10)-C(11)	1.3871(17)
C(11)-C(12)	1.3828(18)
C(12)-C(13)	1.3873(18)
O(5)-S(1)-O(6)	119.91(6)
O(5)-S(1)-O(4)	105.17(5)
O(6)-S(1)-O(4)	110.44(5)
O(5)-S(1)-C(8)	108.44(6)
O(6)-S(1)-C(8)	108.96(6)
O(4)-S(1)-C(8)	102.49(5)
C(4)-O(1)-C(3)	119.15(10)

N(1)-O(3)-C(14)	110.18(9)
C(5)-O(4)-S(1)	122.69(7)
C(4)-N(1)-O(3)	114.92(9)
C(4)-N(1)-C(1)	129.08(10)
O(3)-N(1)-C(1)	113.69(9)
O(8)-N(2)-O(7)	123.92(12)
O(8)-N(2)-C(11)	117.85(11)
O(7)-N(2)-C(11)	118.22(11)
N(1)-C(1)-C(2)	108.01(10)
N(1)-C(1)-C(5)	109.91(10)
C(2)-C(1)-C(5)	117.06(10)
C(3)-C(2)-C(1)	111.27(11)
O(1)-C(3)-C(2)	111.23(11)
O(2)-C(4)-O(1)	119.85(11)
O(2)-C(4)-N(1)	123.62(12)
O(1)-C(4)-N(1)	116.49(11)
O(4)-C(5)-C(6)	107.00(9)
O(4)-C(5)-C(1)	108.65(9)
C(6)-C(5)-C(1)	113.12(10)
C(5)-C(6)-C(7)	113.07(11)
C(13)-C(8)-C(9)	122.26(12)
C(13)-C(8)-S(1)	119.11(10)
C(9)-C(8)-S(1)	118.62(9)
C(10)-C(9)-C(8)	118.91(11)
C(9)-C(10)-C(11)	118.23(11)
C(12)-C(11)-C(10)	123.36(12)
C(12)-C(11)-N(2)	118.45(11)
C(10)-C(11)-N(2)	118.17(11)
C(11)-C(12)-C(13)	118.37(11)
C(12)-C(13)-C(8)	118.86(11)

Symmetry transformations used to generate equivalent atoms:

Table S4. Anisotropic displacement parameters ($Å^2$x 10^3) for SS-12-69. The anisotropic displacement factor exponent takes the form: $-2\pi^2[h^2 a^{*2}U^{11} + ... + 2 h k a^* b^* U^{12}]$

	U^{11}	U^{22}	U^{33}	U^{23}	U^{13}	U^{12}
S(1)	19(1)	17(1)	18(1)	0(1)	1(1)	-1(1)
O(1)	25(1)	19(1)	27(1)	-2(1)	3(1)	-1(1)
O(1W)	56(4)	31(3)	41(3)	-2(2)	20(2)	-10(2)
O(2)	20(1)	28(1)	24(1)	-4(1)	4(1)	-6(1)
O(3)	17(1)	20(1)	23(1)	-1(1)	1(1)	3(1)
O(4)	17(1)	16(1)	19(1)	2(1)	2(1)	2(1)
O(5)	23(1)	27(1)	22(1)	2(1)	-3(1)	-4(1)
O(6)	30(1)	18(1)	25(1)	-3(1)	7(1)	1(1)
O(7)	27(1)	20(1)	42(1)	1(1)	3(1)	-3(1)
O(8)	17(1)	35(1)	74(1)	10(1)	-3(1)	-2(1)
N(1)	14(1)	18(1)	23(1)	-1(1)	2(1)	1(1)
N(2)	20(1)	24(1)	30(1)	-2(1)	1(1)	-2(1)
C(1)	15(1)	21(1)	19(1)	-1(1)	2(1)	-1(1)
C(2)	18(1)	27(1)	33(1)	-11(1)	5(1)	-1(1)
C(3)	24(1)	21(1)	36(1)	-8(1)	-2(1)	4(1)
C(4)	21(1)	22(1)	15(1)	-2(1)	1(1)	-3(1)
C(5)	17(1)	17(1)	18(1)	2(1)	1(1)	1(1)
C(6)	18(1)	22(1)	24(1)	3(1)	2(1)	-3(1)
C(7)	22(1)	24(1)	34(1)	-1(1)	-1(1)	-6(1)
C(8)	18(1)	20(1)	16(1)	0(1)	2(1)	1(1)
C(9)	17(1)	21(1)	22(1)	2(1)	1(1)	2(1)
C(10)	21(1)	19(1)	23(1)	1(1)	2(1)	2(1)
C(11)	18(1)	21(1)	19(1)	-1(1)	1(1)	-1(1)
C(12)	17(1)	25(1)	21(1)	1(1)	1(1)	4(1)
C(13)	21(1)	19(1)	20(1)	1(1)	3(1)	4(1)
C(14)	21(1)	26(1)	24(1)	3(1)	-1(1)	2(1)

Table S5. Hydrogen coordinates (x 10^4) and isotropic displacement parameters (Å^{2}x 10 ^{3}) for SS-12-69.

	x	y	z	U(eq)
H(1)	2750(20)	6799(10)	450(13)	22
H(2A)	3330(30)	5562(11)	46(15)	31
H(2B)	5080(30)	5707(11)	926(14)	31
H(3A)	3630(30)	4478(12)	1130(14)	34
H(3B)	3590(30)	5022(12)	2074(15)	34
H(5)	3190(20)	7338(10)	2052(12)	21
H(6A)	5400(20)	7580(11)	1042(13)	26
H(6B)	6480(30)	6850(11)	1542(13)	26
H(7A)	7910(30)	7990(12)	2367(15)	41
H(7B)	7060(30)	7500(13)	3143(16)	41
H(7C)	5980(30)	8257(13)	2630(15)	41
H(9)	3790(20)	4910(10)	4249(13)	25
H(10)	1370(20)	3975(11)	4221(13)	25
H(12)	-2370(30)	5704(10)	3195(13)	26
H(13)	-70(20)	6665(11)	3228(13)	24
H(14A)	-10(30)	7230(12)	-260(15)	37
H(14B)	-1690(30)	7572(13)	173(15)	37
H(14C)	-1740(30)	6669(12)	-147(15)	37

Table S6. Torsion angles [°] for SS-12-69.

O(5)-S(1)-O(4)-C(5)	-143.36(8)
O(6)-S(1)-O(4)-C(5)	-12.63(10)
C(8)-S(1)-O(4)-C(5)	103.33(9)
C(14)-O(3)-N(1)-C(4)	-79.77(12)
C(14)-O(3)-N(1)-C(1)	84.58(12)
C(4)-N(1)-C(1)-C(2)	-1.70(17)
O(3)-N(1)-C(1)-C(2)	-163.32(10)
C(4)-N(1)-C(1)-C(5)	-130.50(12)
O(3)-N(1)-C(1)-C(5)	67.87(12)
N(1)-C(1)-C(2)-C(3)	-35.07(14)
C(5)-C(1)-C(2)-C(3)	89.56(13)
C(4)-O(1)-C(3)-C(2)	-42.83(16)
C(1)-C(2)-C(3)-O(1)	57.32(14)
C(3)-O(1)-C(4)-O(2)	-171.59(12)
C(3)-O(1)-C(4)-N(1)	6.01(16)
O(3)-N(1)-C(4)-O(2)	-2.80(17)
C(1)-N(1)-C(4)-O(2)	-164.24(12)
O(3)-N(1)-C(4)-O(1)	179.69(9)
C(1)-N(1)-C(4)-O(1)	18.25(18)
S(1)-O(4)-C(5)-C(6)	117.02(9)
S(1)-O(4)-C(5)-C(1)	-120.53(9)
N(1)-C(1)-C(5)-O(4)	72.38(12)
C(2)-C(1)-C(5)-O(4)	-51.29(14)
N(1)-C(1)-C(5)-C(6)	-168.96(10)
C(2)-C(1)-C(5)-C(6)	67.37(14)
O(4)-C(5)-C(6)-C(7)	-64.17(13)
C(1)-C(5)-C(6)-C(7)	176.21(11)
O(5)-S(1)-C(8)-C(13)	151.62(10)
O(6)-S(1)-C(8)-C(13)	19.51(12)
O(4)-S(1)-C(8)-C(13)	-97.51(10)
O(5)-S(1)-C(8)-C(9)	-29.51(11)
O(6)-S(1)-C(8)-C(9)	-161.61(10)
O(4)-S(1)-C(8)-C(9)	81.36(10)
C(13)-C(8)-C(9)-C(10)	-0.65(19)

S(1)-C(8)-C(9)-C(10)	-179.49(9)
C(8)-C(9)-C(10)-C(11)	0.27(18)
C(9)-C(10)-C(11)-C(12)	0.30(19)
C(9)-C(10)-C(11)-N(2)	-178.19(11)
O(8)-N(2)-C(11)-C(12)	4.29(19)
O(7)-N(2)-C(11)-C(12)	-174.89(12)
O(8)-N(2)-C(11)-C(10)	-177.15(13)
O(7)-N(2)-C(11)-C(10)	3.67(18)
C(10)-C(11)-C(12)-C(13)	-0.49(19)
N(2)-C(11)-C(12)-C(13)	177.99(11)
C(11)-C(12)-C(13)-C(8)	0.11(18)
C(9)-C(8)-C(13)-C(12)	0.46(19)
S(1)-C(8)-C(13)-C(12)	179.29(9)

Symmetry transformations used to generate equivalent atoms:

VII. NMR Spectra

Compound 2 ($CDCl_3$, ^1H NMR: 600 MHz, $^{13}C\{^1H\}$ NMR: 101 MHz)

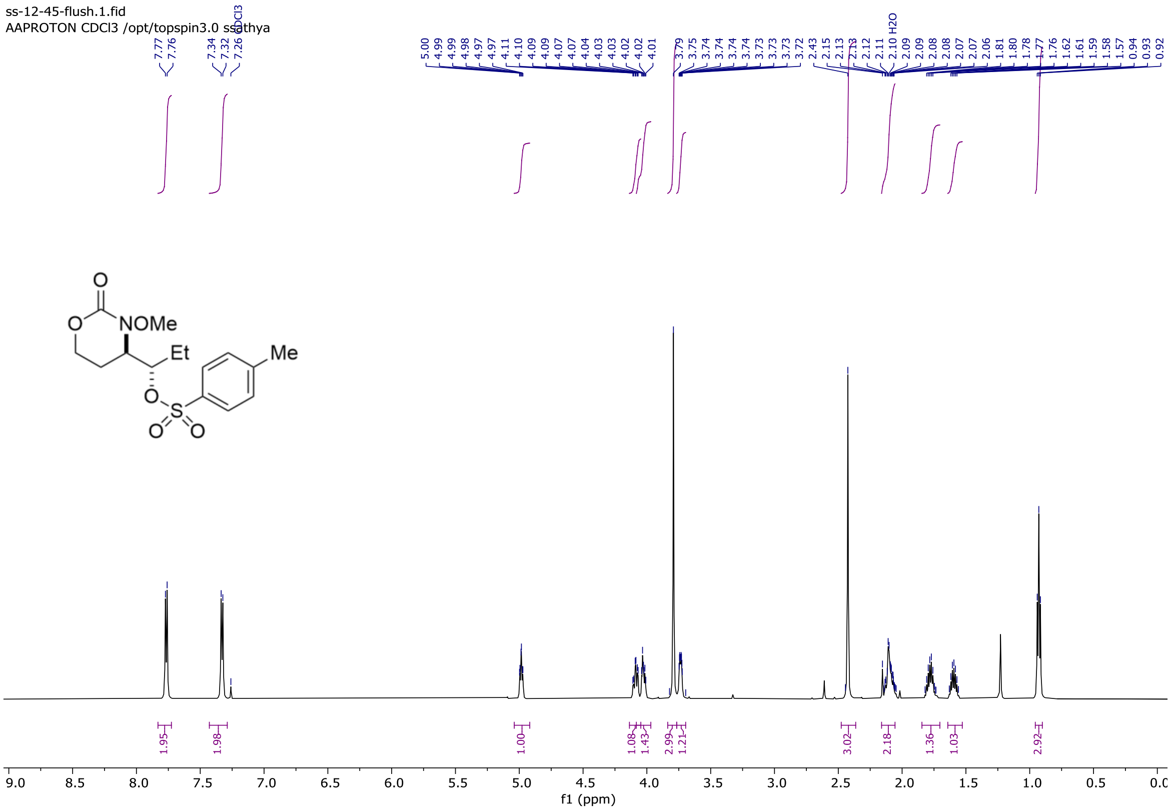

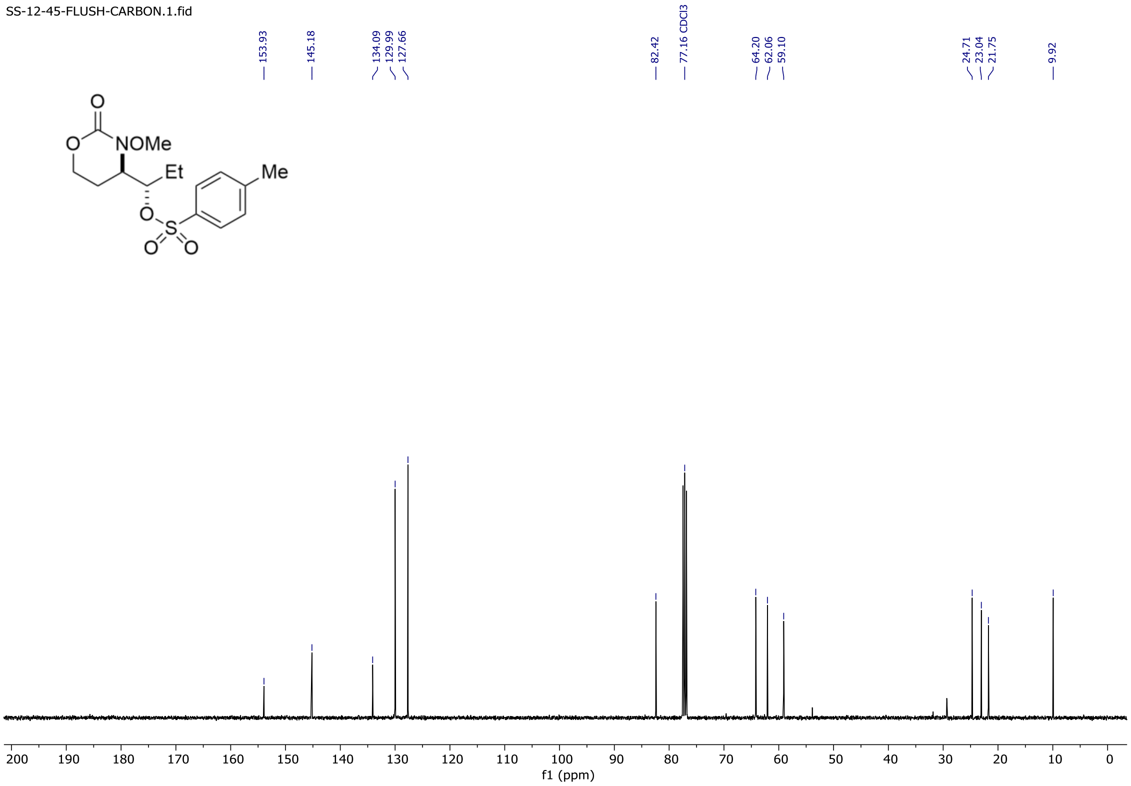

Compound 4 (CDCl₃, ^{1}H NMR: 400 MHz, ^{13}C{^{1}H} NMR: 101 MHz)

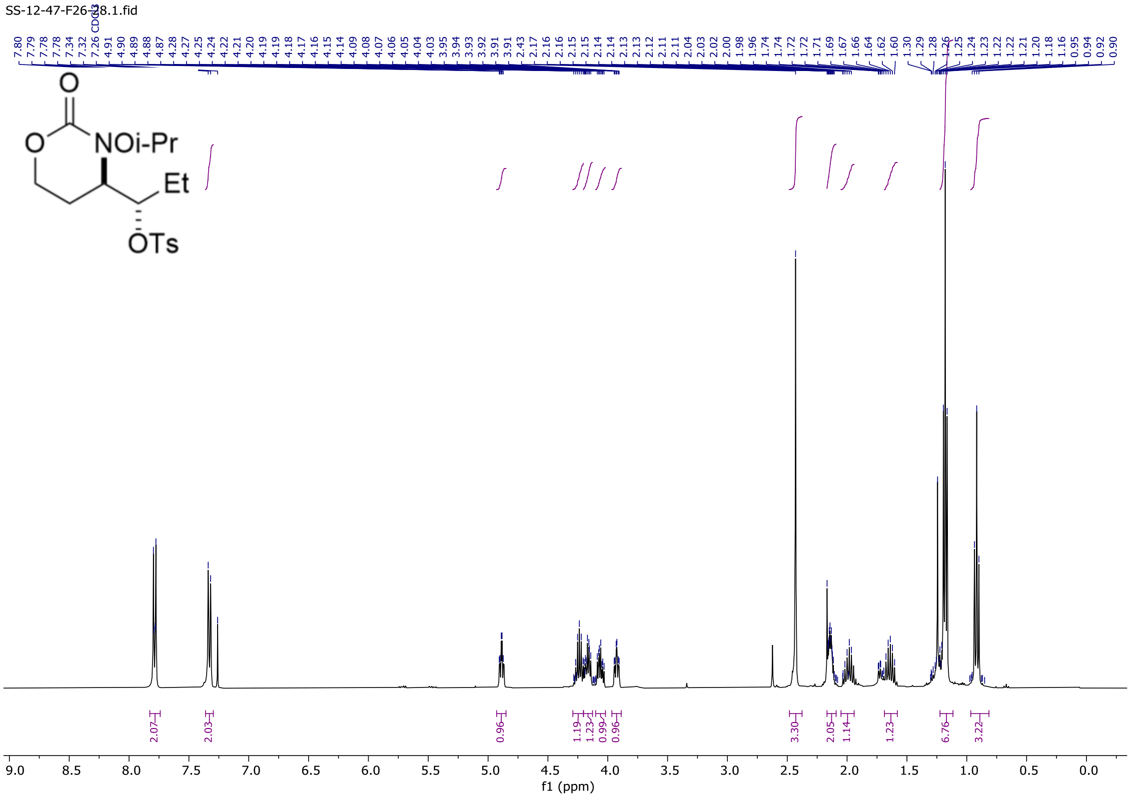

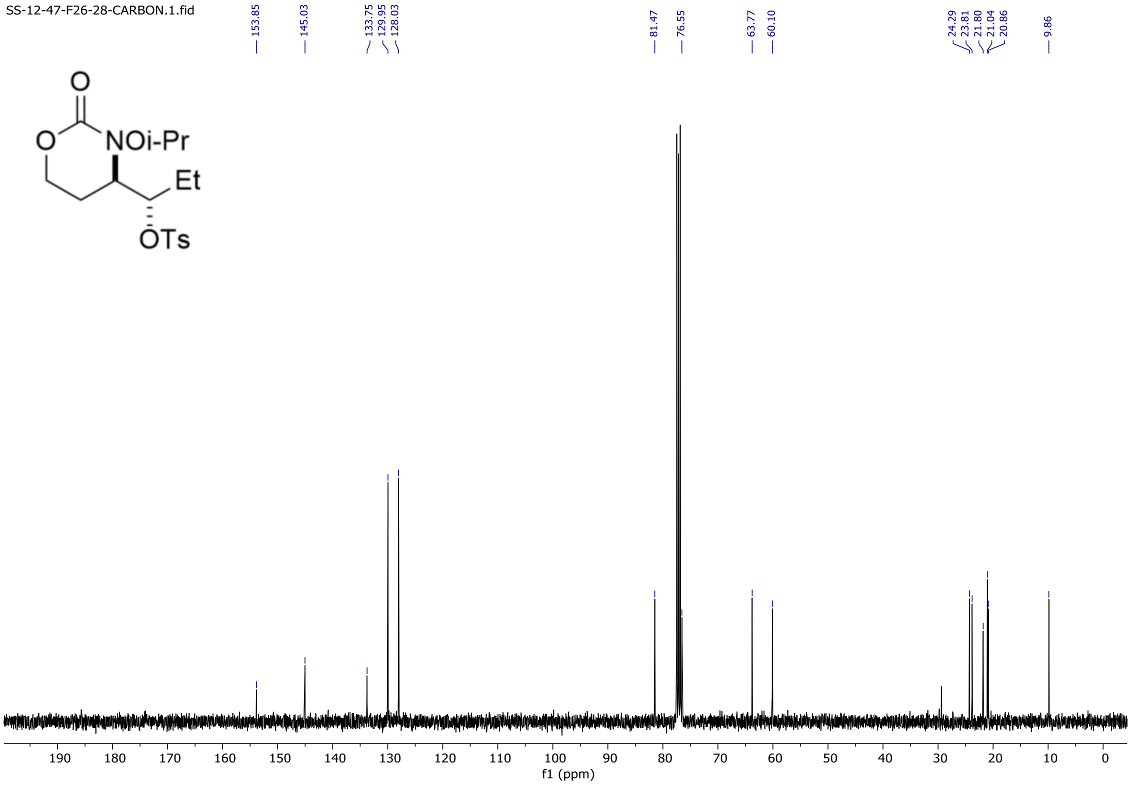

Compound 6 ($CDCl_3$, ^{1}H NMR: 400 MHz, $^{13}C\{^{1}H\}$ NMR: 101 MHz)

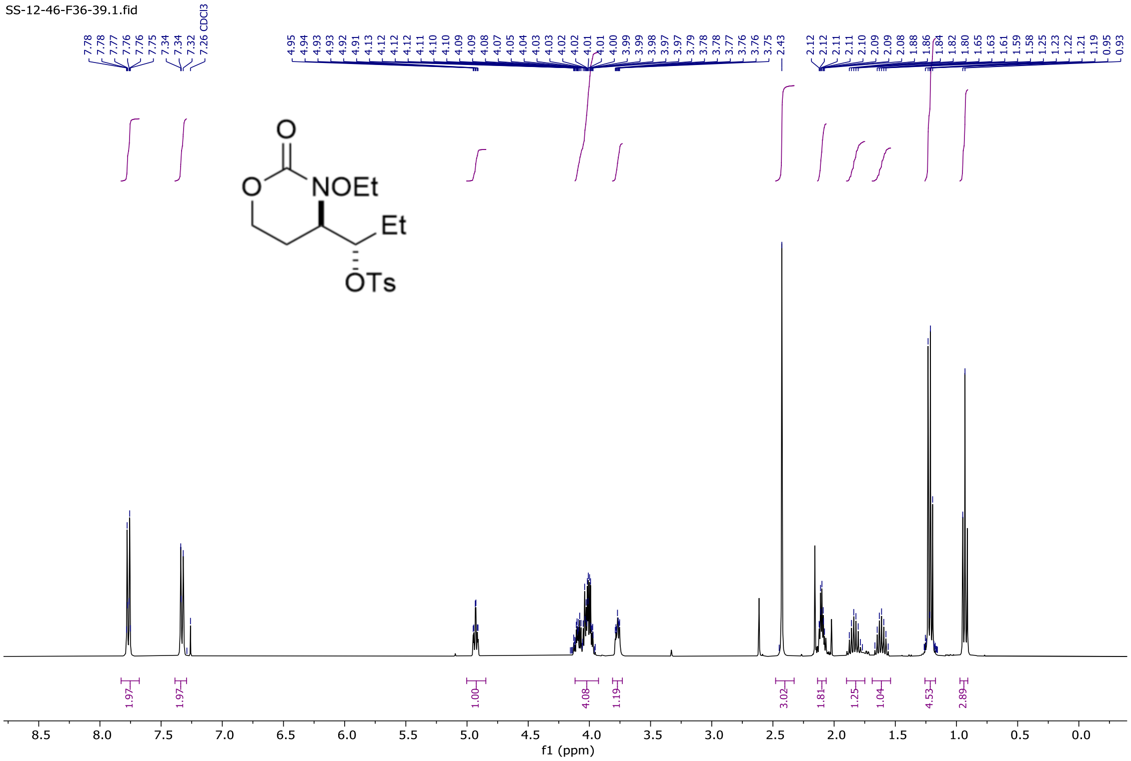

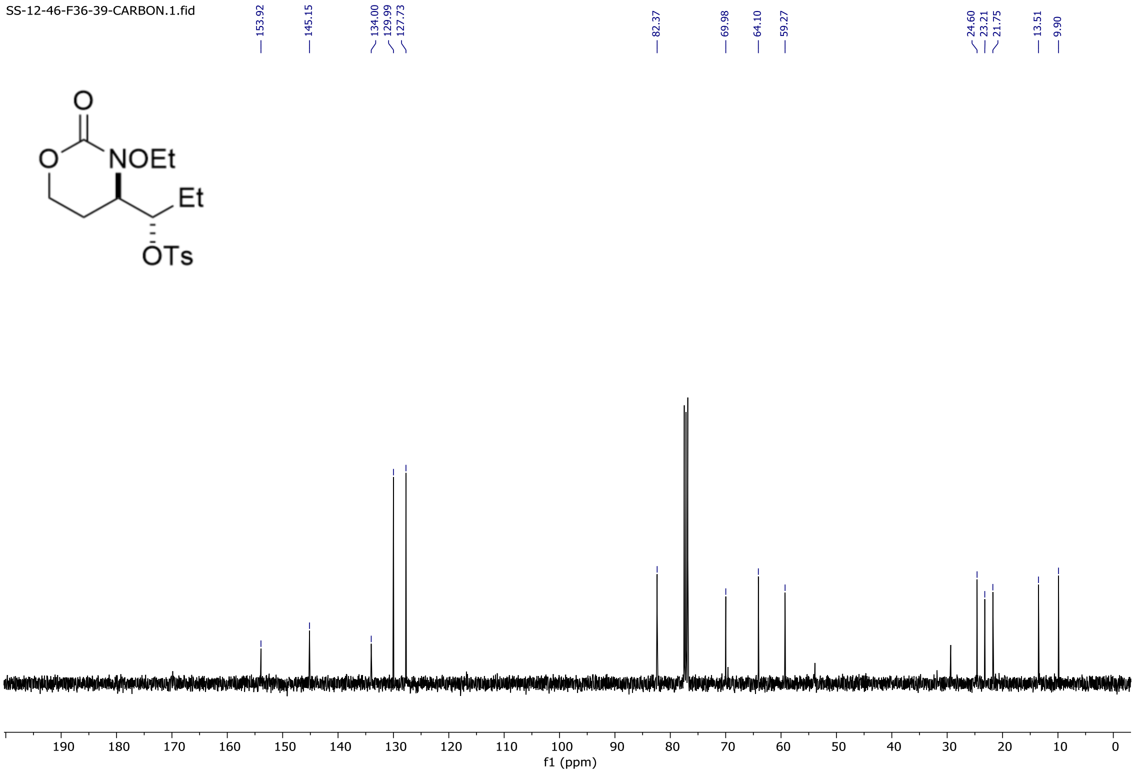

Compound 8 ($CDCl_3$, ^1H NMR: 400 MHz, $^{13}C\{^1H\}$ NMR: 101 MHz)

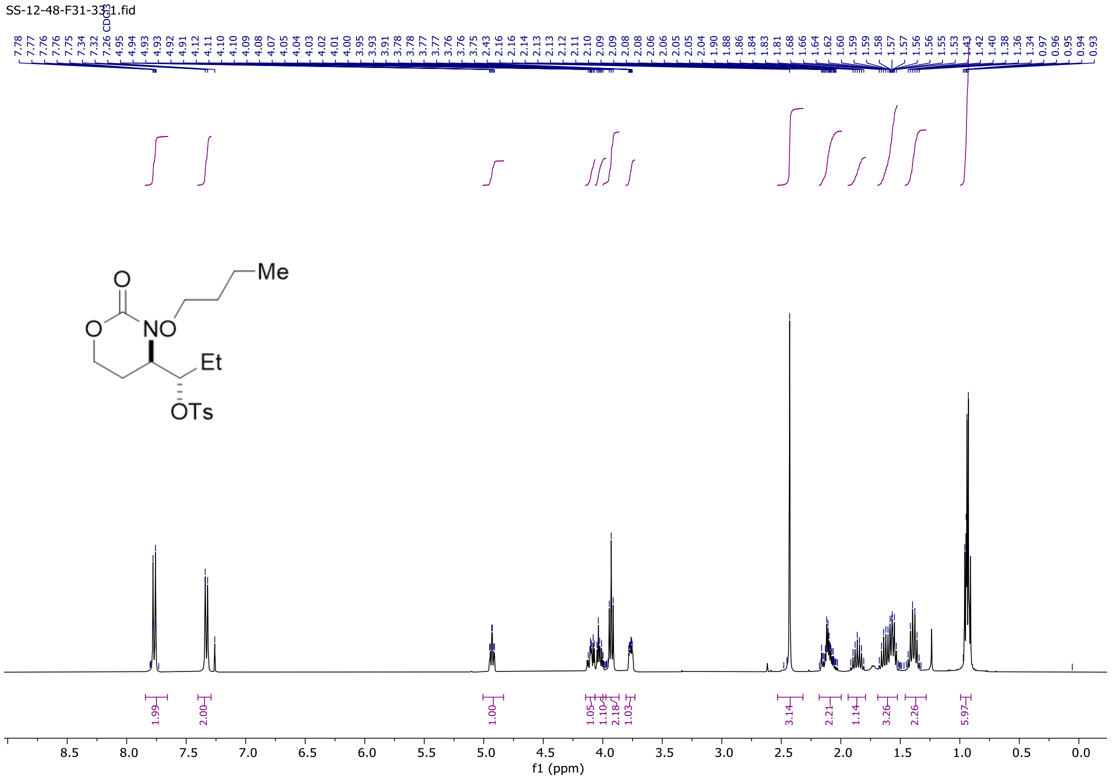

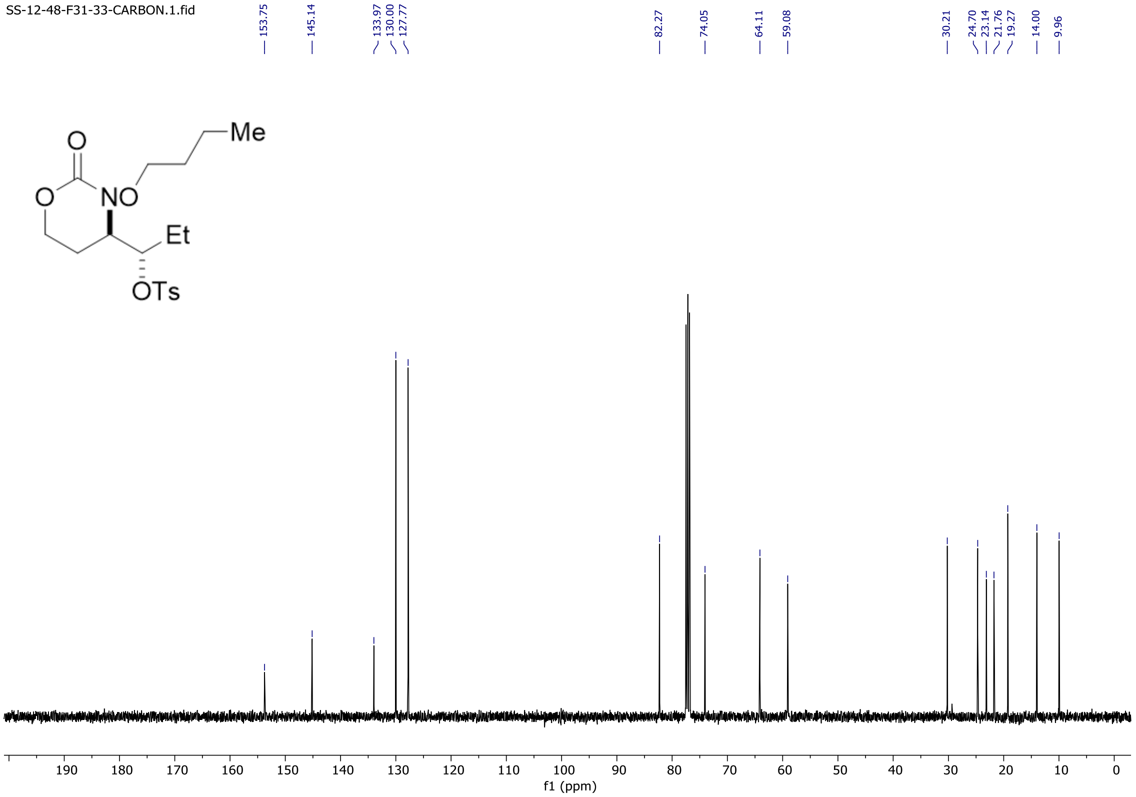

Compound 10 ($CDCl_3$, ^{1}H NMR: 400 MHz, $^{13}C\{^1H\}$ NMR: 101 MHz)

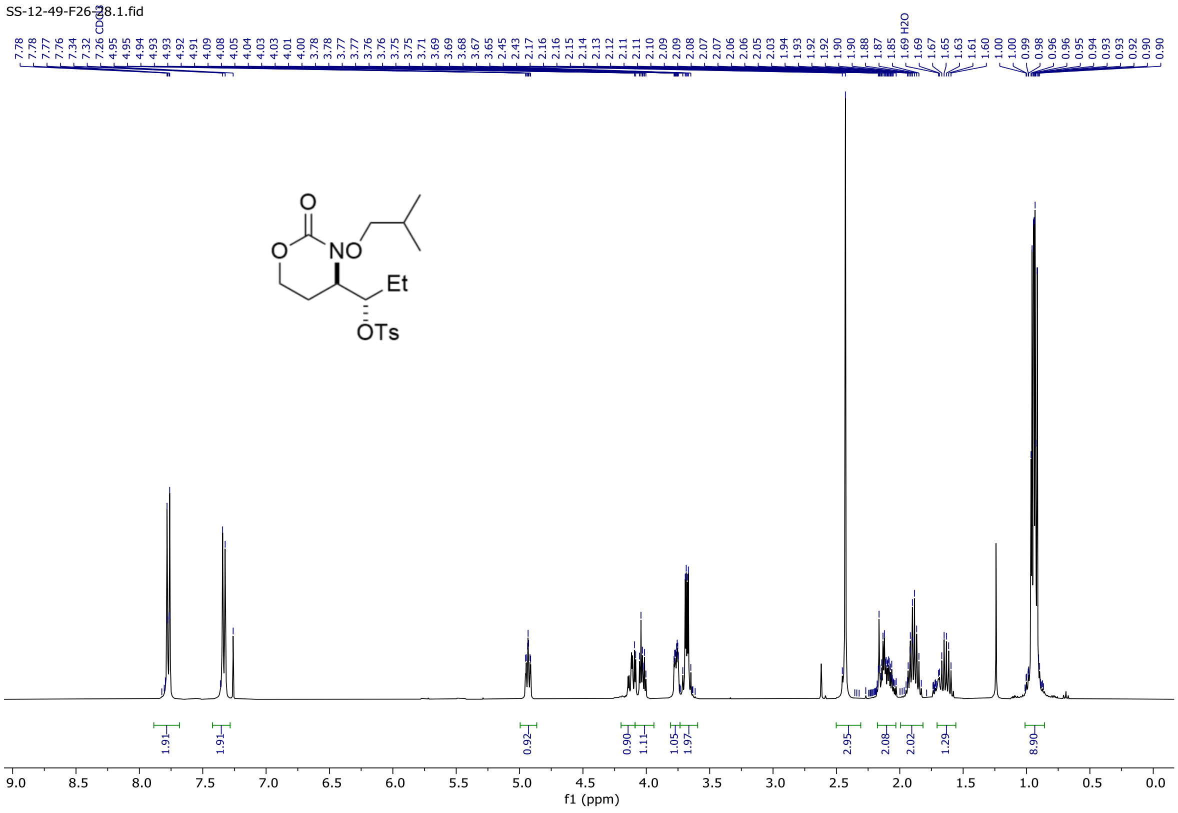

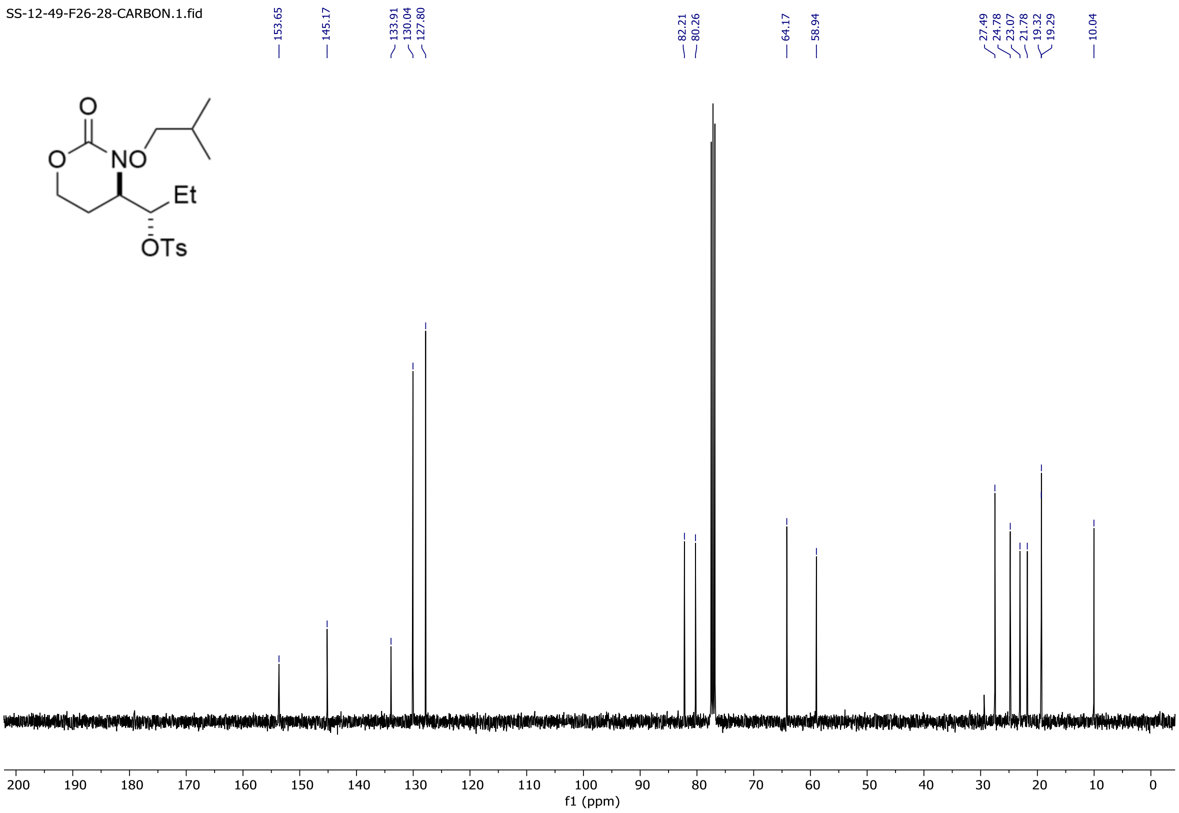

Compound 17 ($CDCl_3$, ^1H NMR: 400 MHz, $^{13}C\{^1H\}$ NMR: 101 MHz)

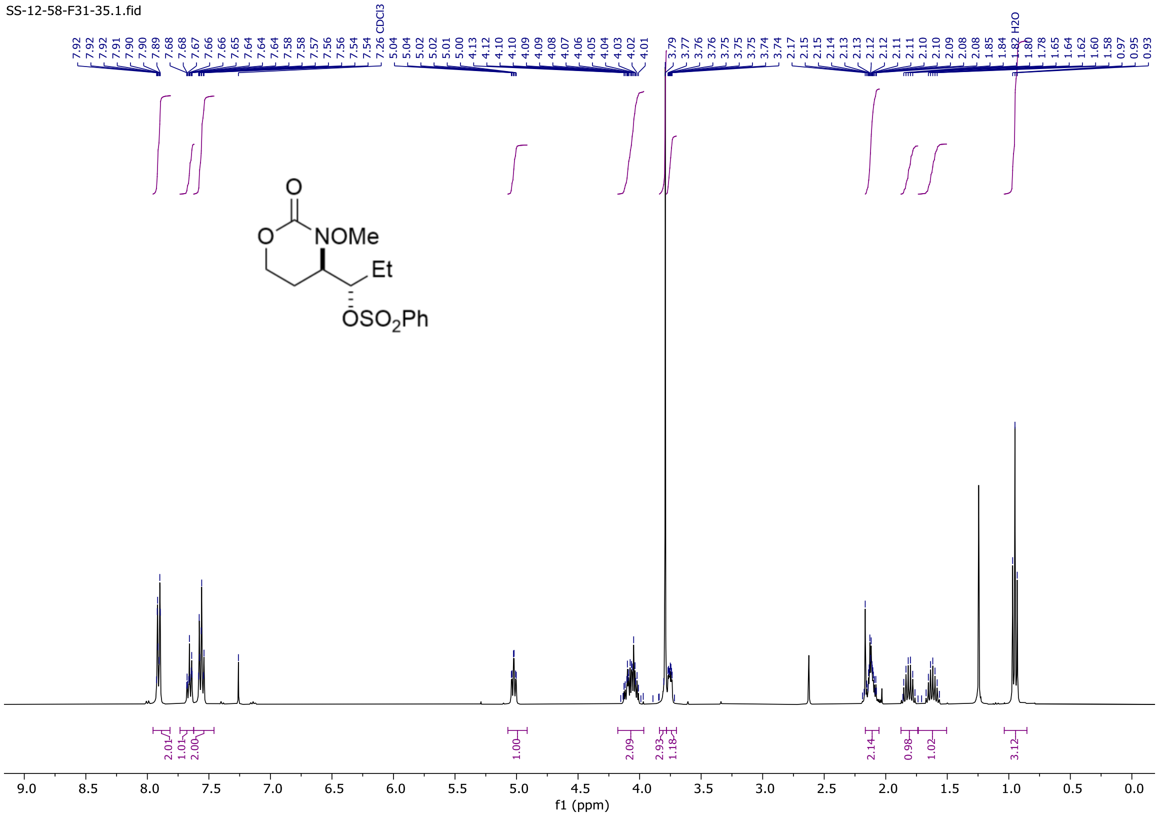

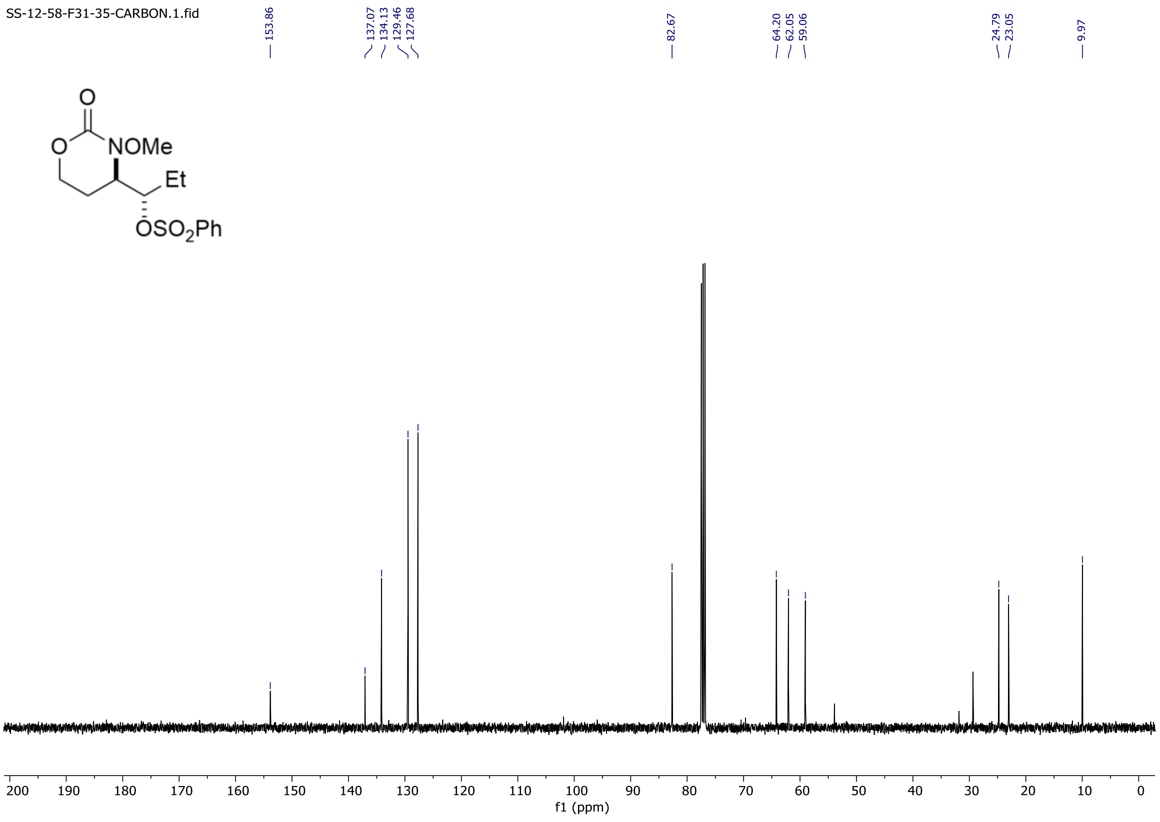

Compound 18 (CDCl$_3$, ^{1}H NMR: 400 MHz, ^{13}C{^{1}H} NMR: 101 MHz)

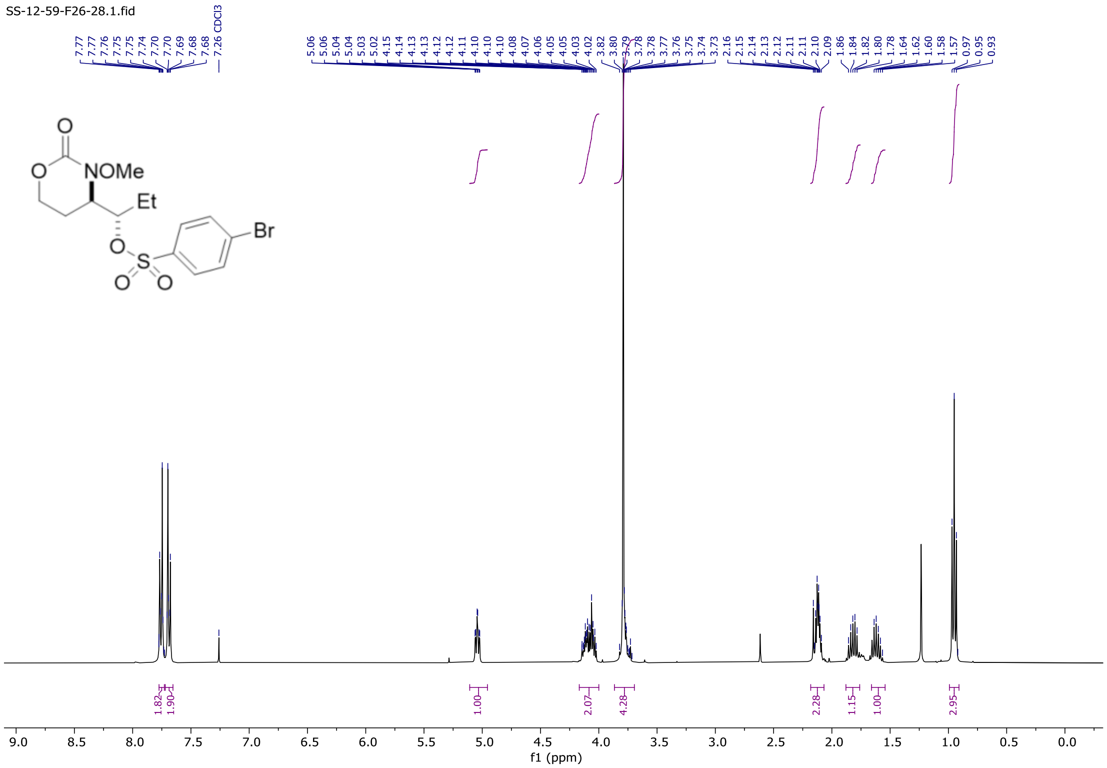

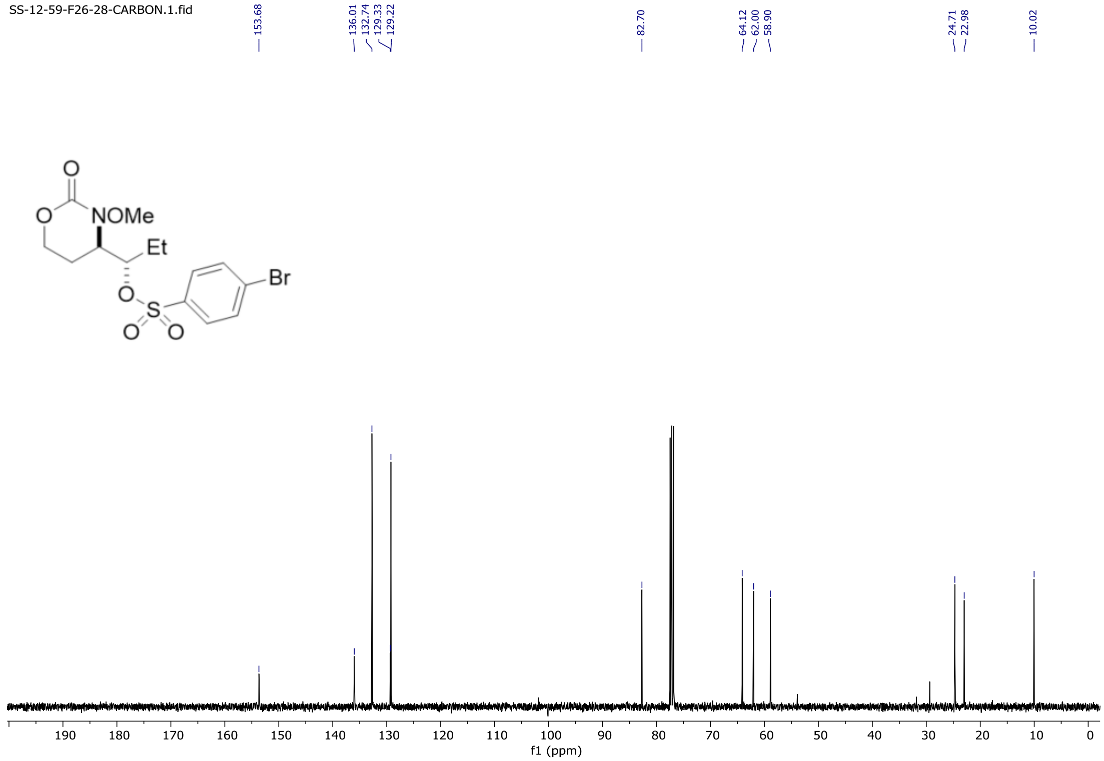

Compound 19 (CDCl$_3$, ^{1}H NMR: 400 MHz, ^{13}C{^{1}H} NMR: 101 MHz)

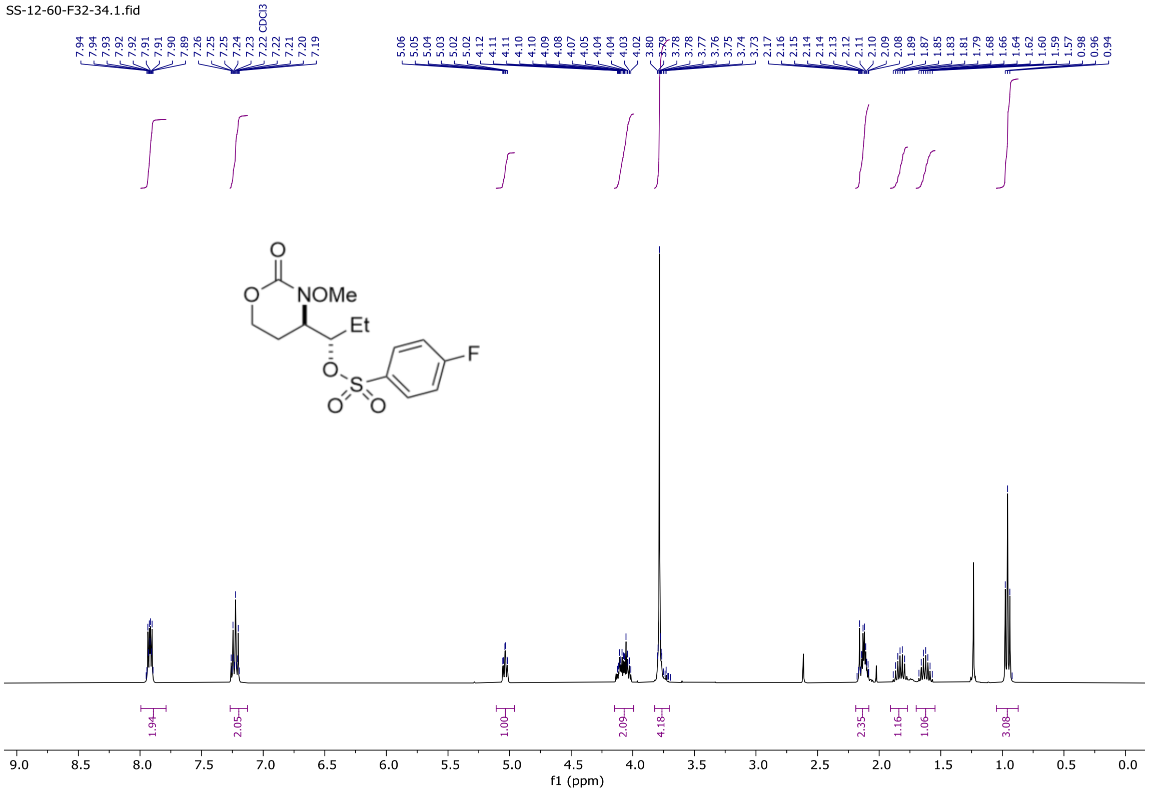

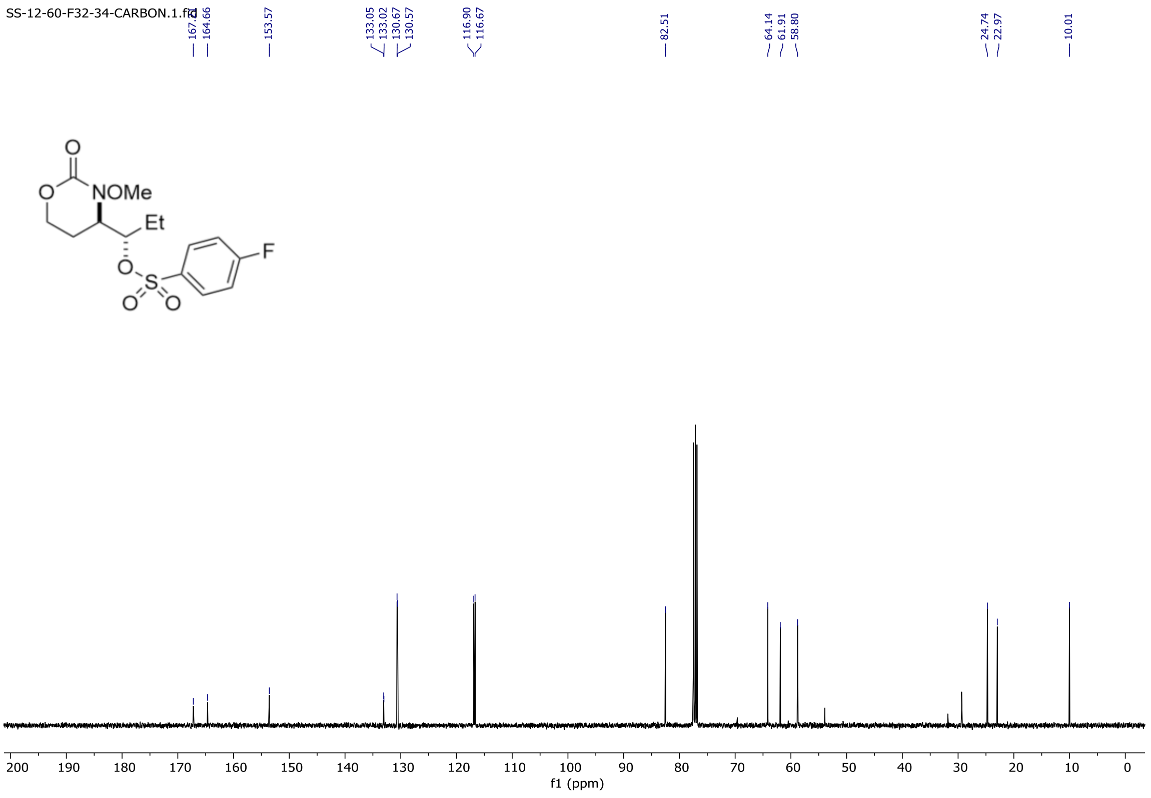

Compound 20 ($CDCl_3$, ^1H NMR: 400 MHz, $^{13}C\{^1H\}$ NMR: 101 MHz)

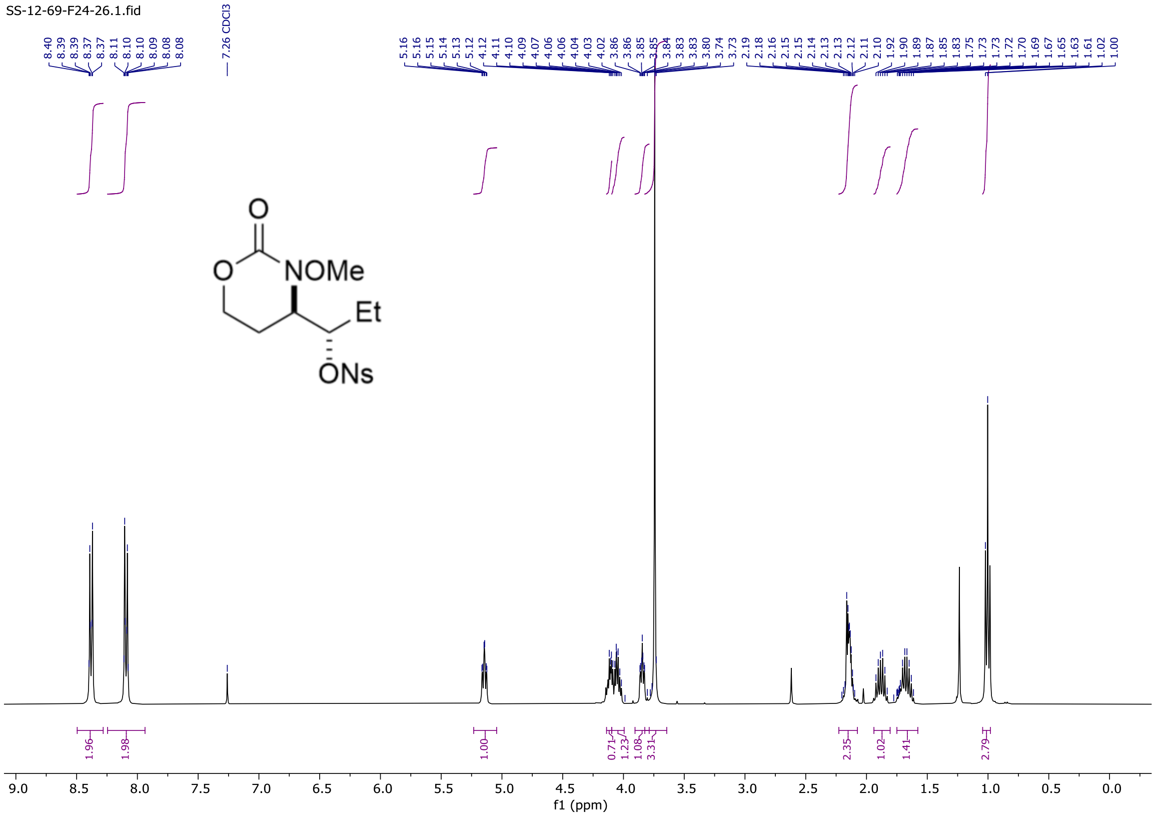

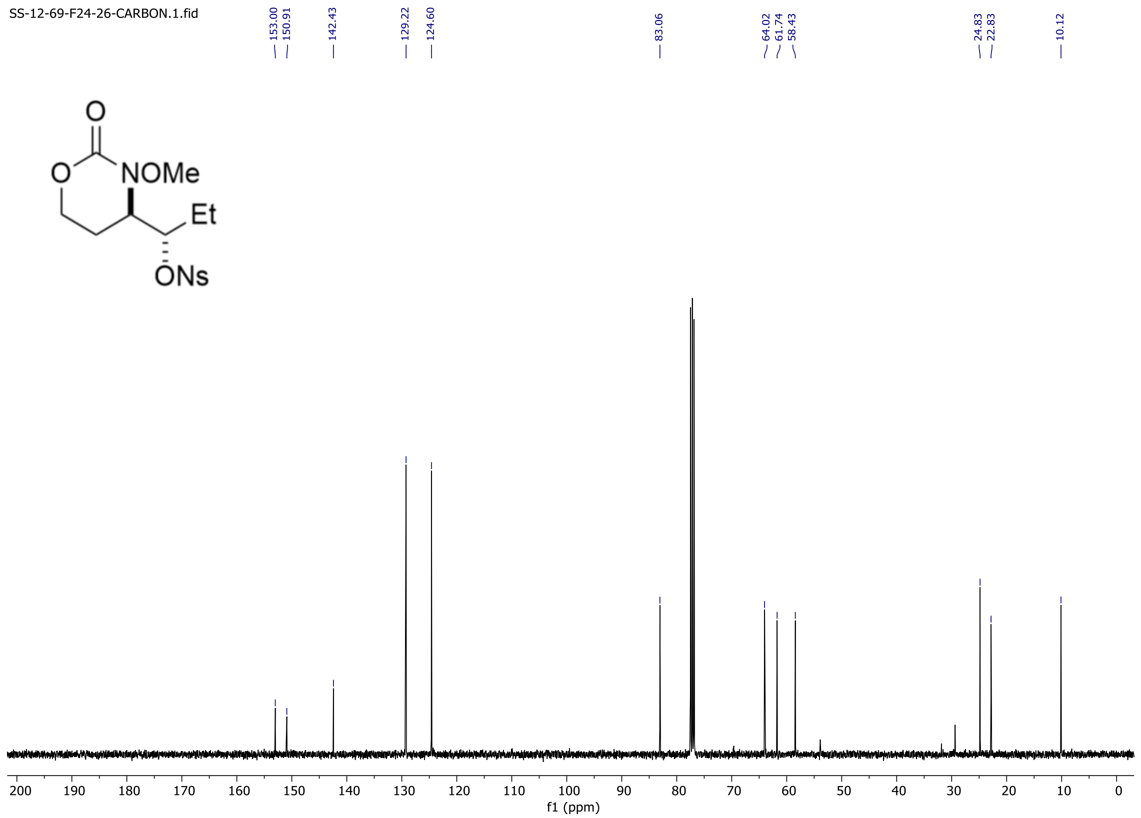

Compound 21 (CDCl₃, ^{1}H NMR: 400 MHz, ^{13}C{^{1}H} NMR: 101 MHz)

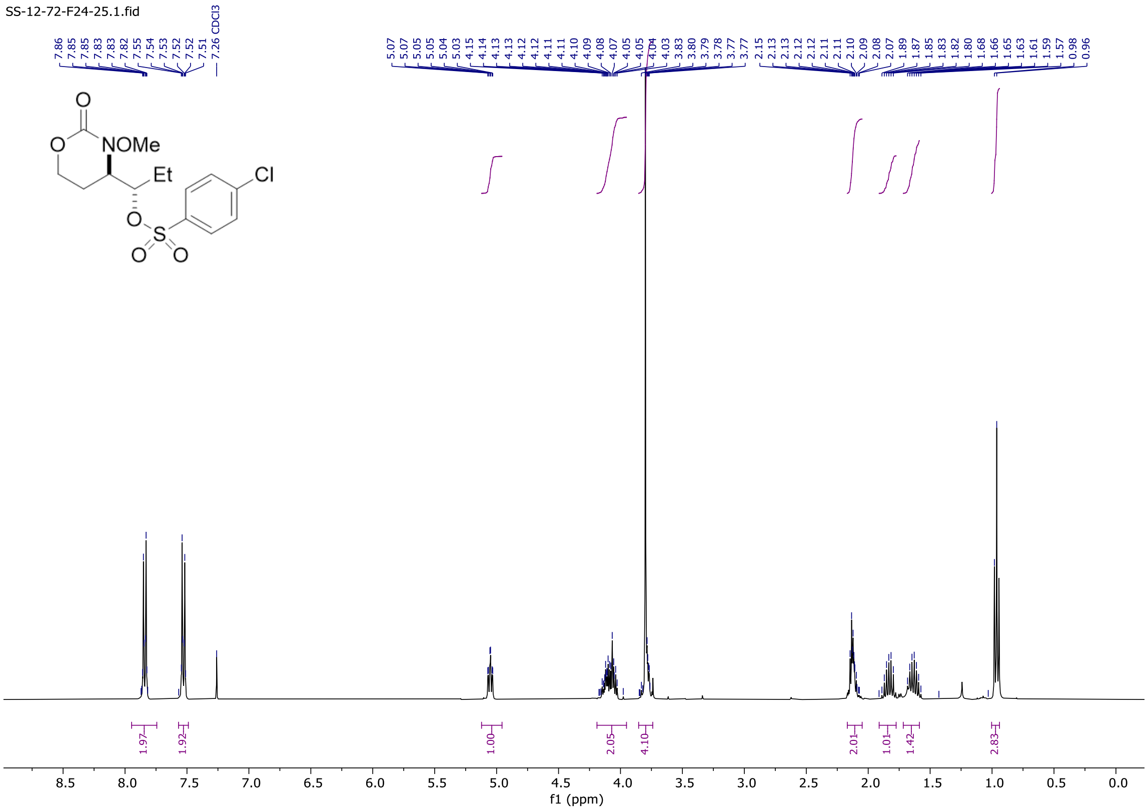

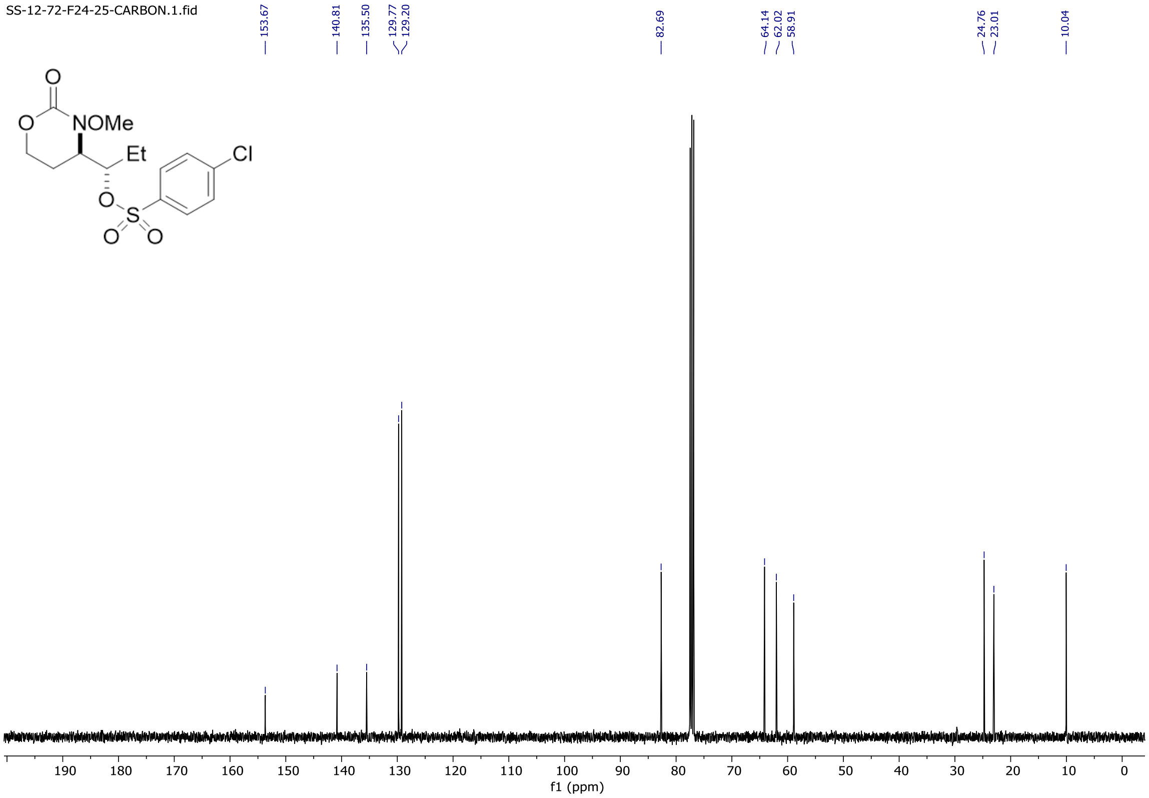

Compound 22 (CDCl₃, ¹H NMR: 400 MHz, ¹³C{¹H} NMR: 101 MHz)

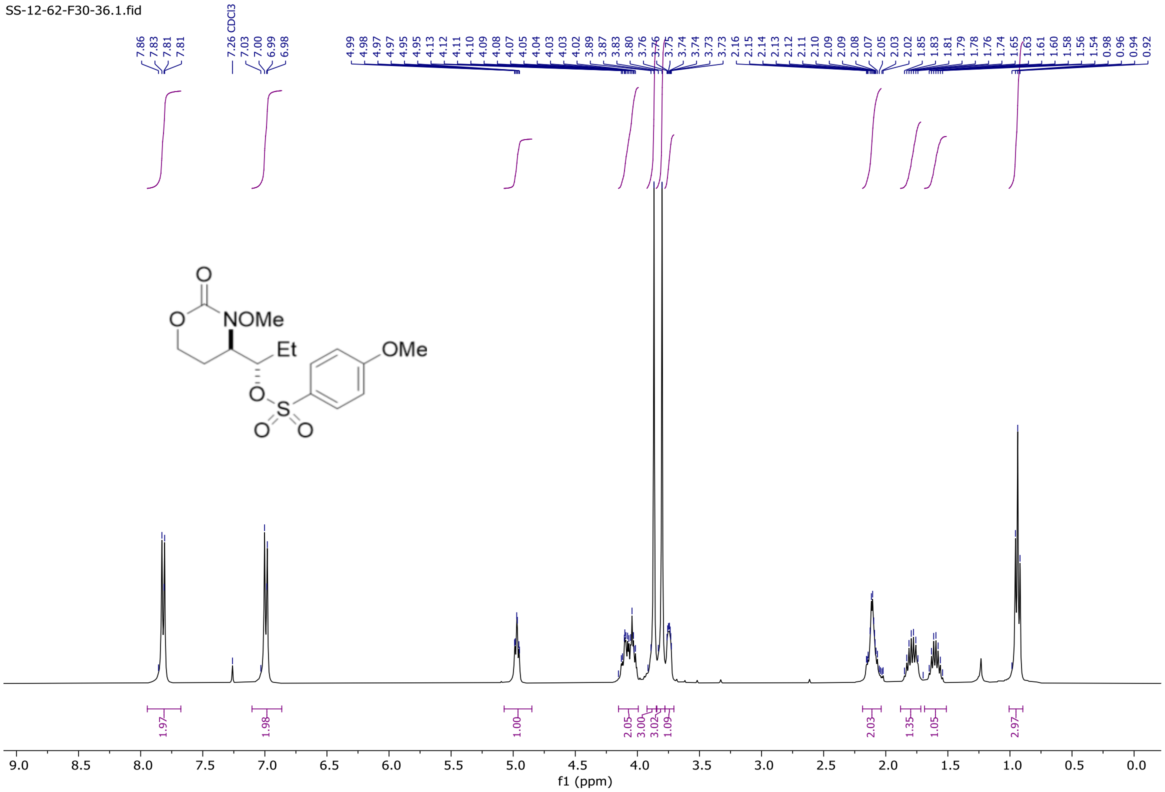

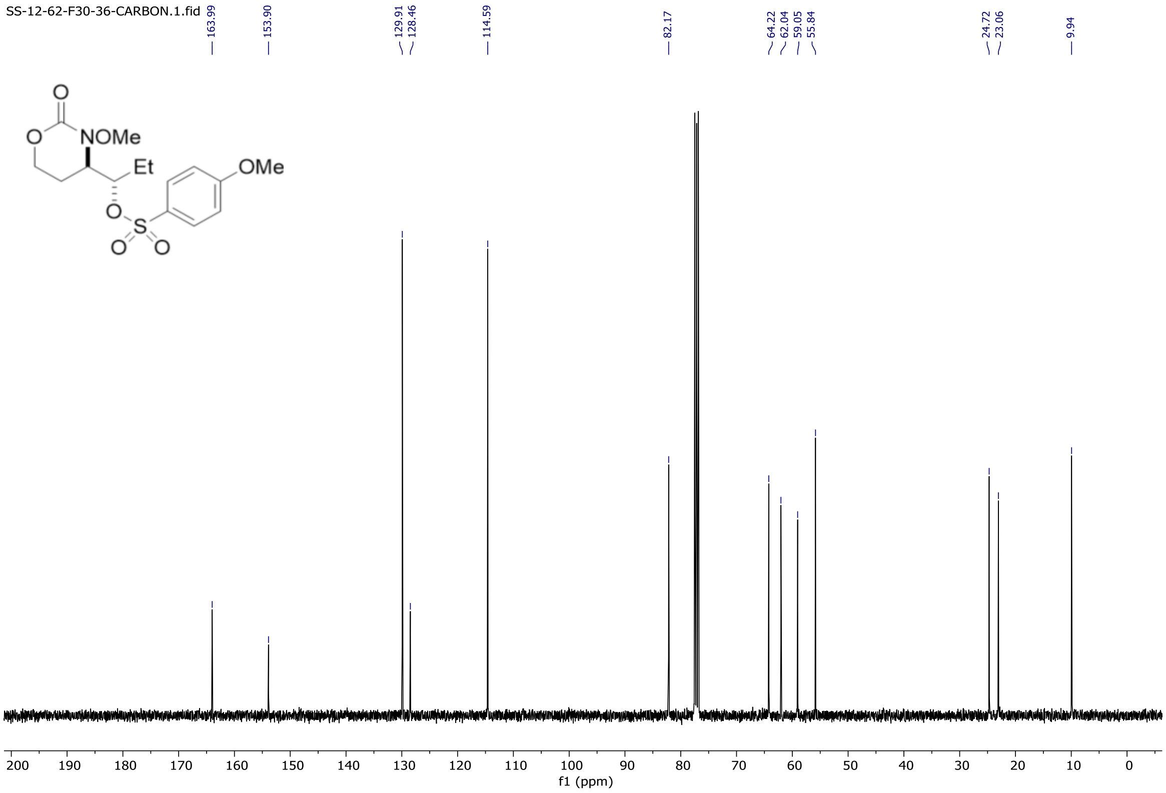

Compound 23 ($CDCl_3$, ^{1}H NMR: 400 MHz, $^{13}C\{^{1}H\}$ NMR: 101 MHz)

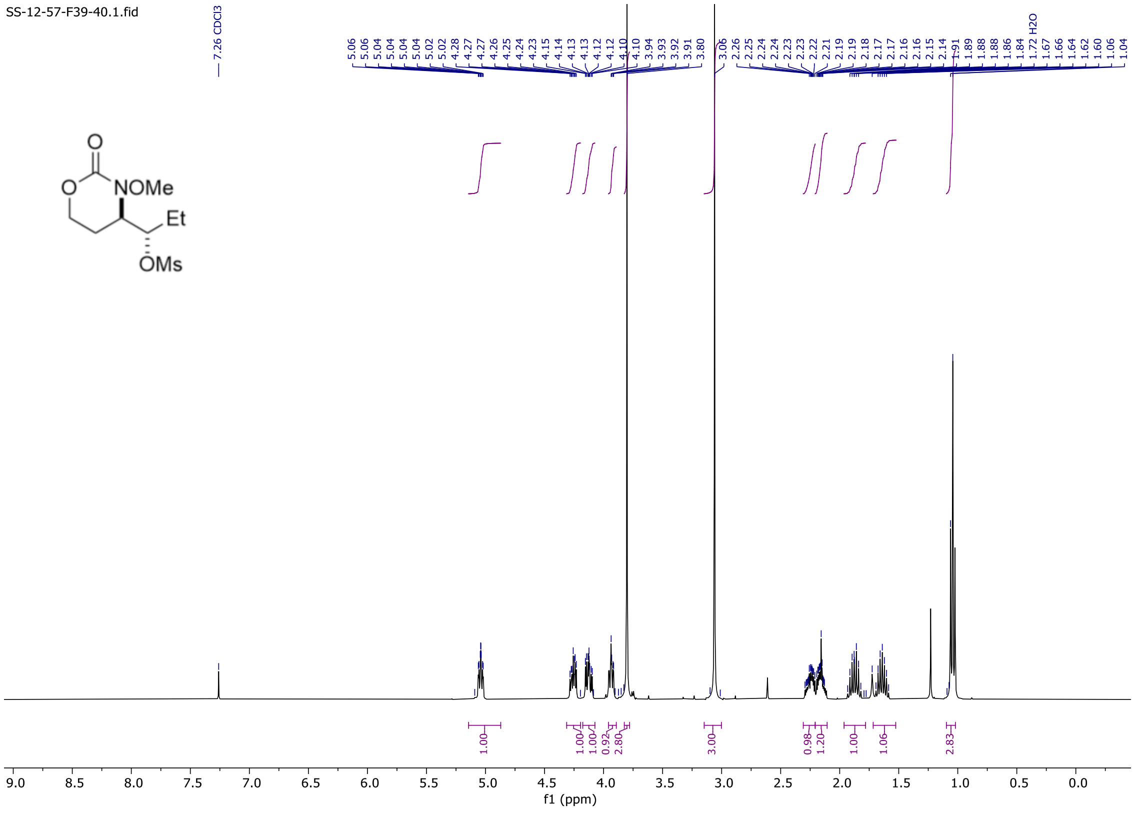

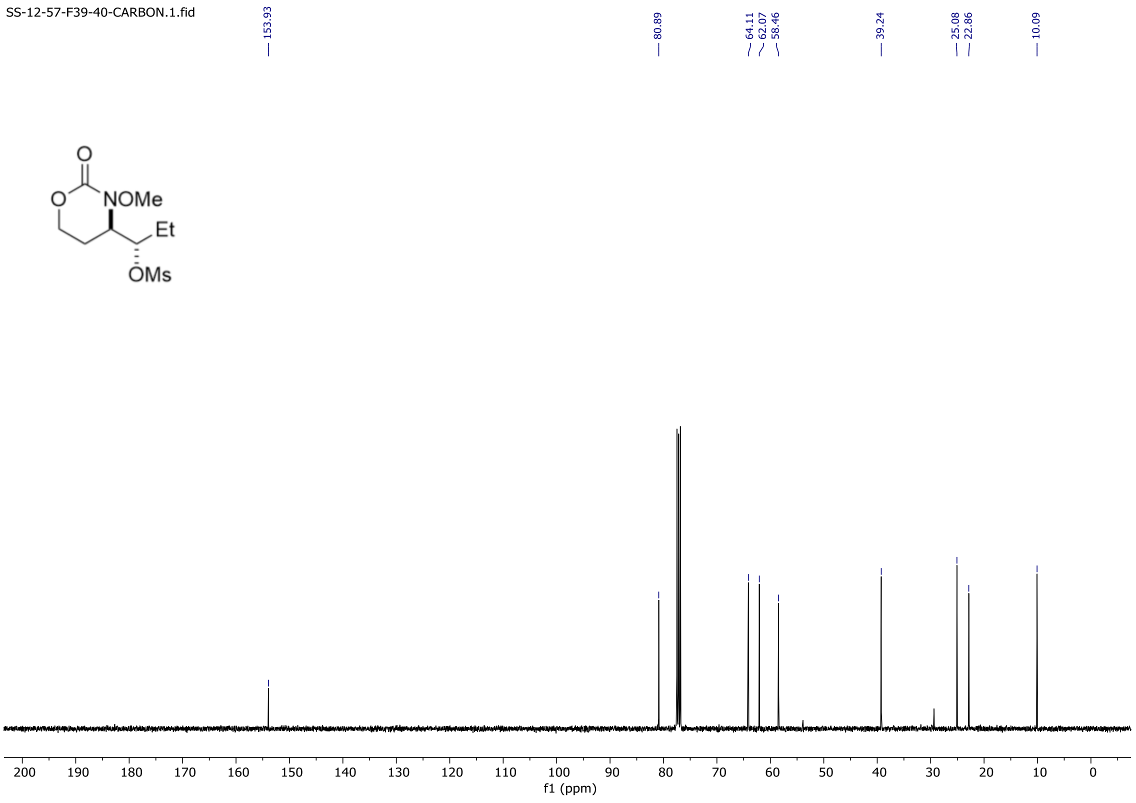

Compound 24 (CDCl₃, ¹H NMR: 400 MHz, ¹³C{¹H} NMR: 101 MHz)

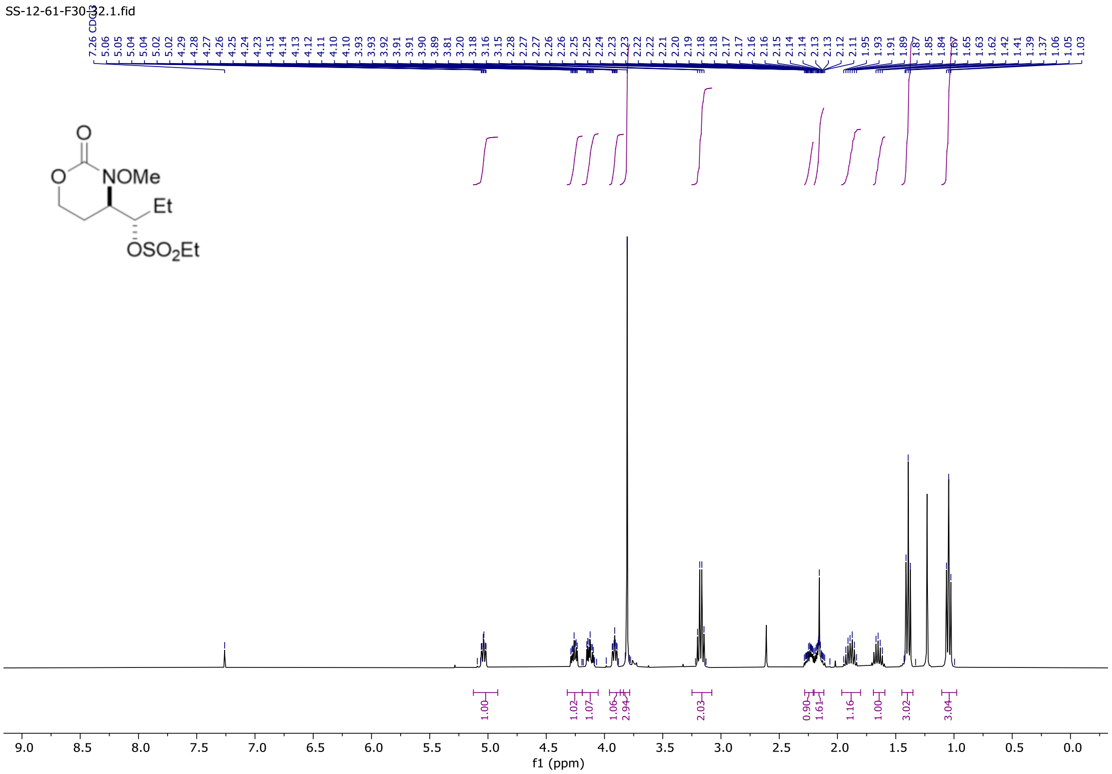

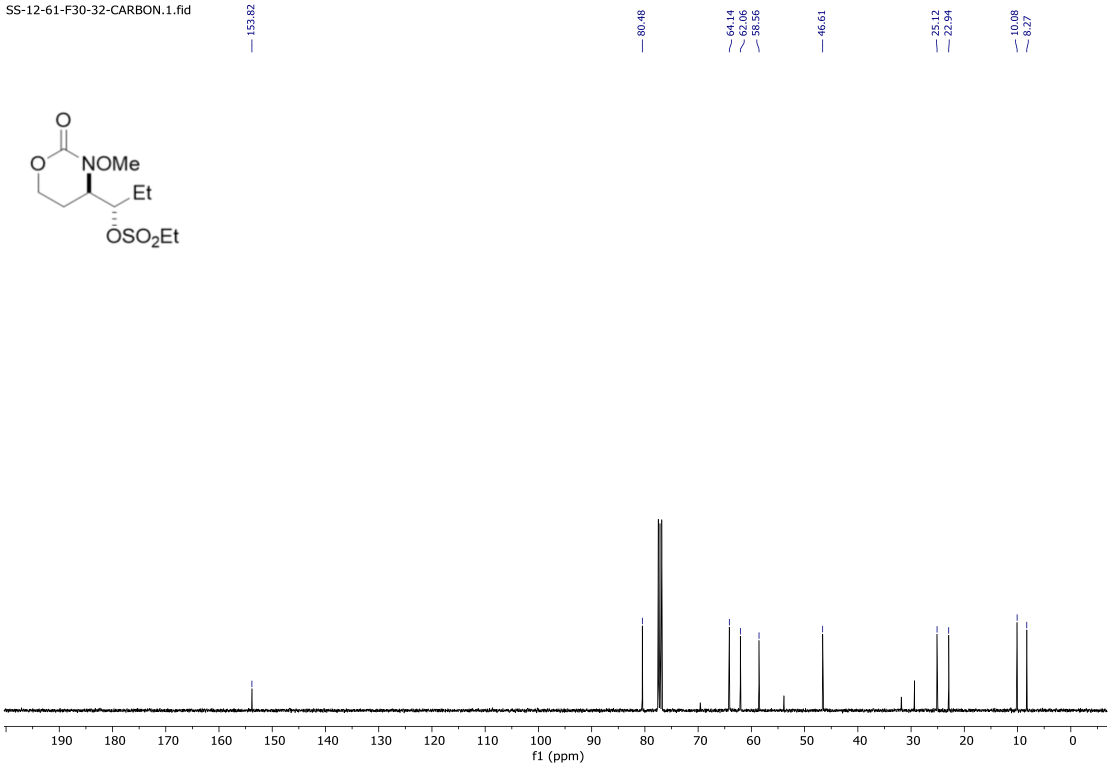

Compound 25 (CDCl₃, ^{1}H NMR: 600 MHz, ^{13}C{^{1}H} NMR: 101 MHz)

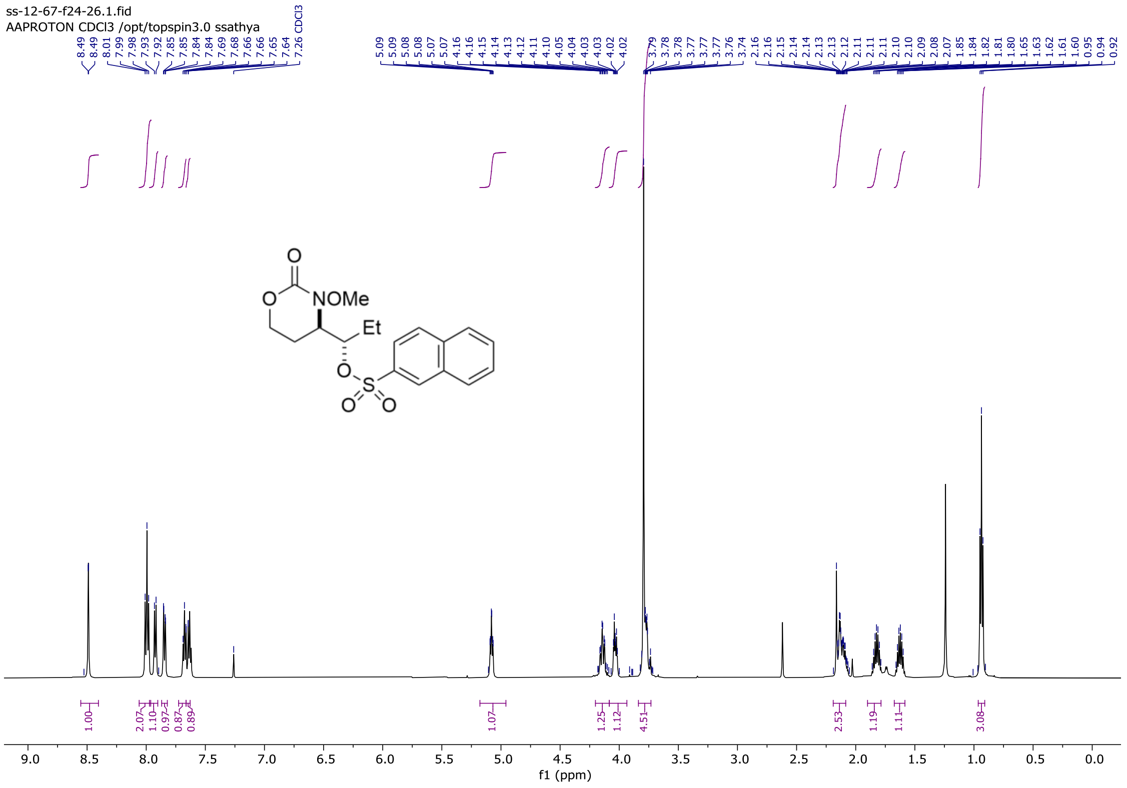

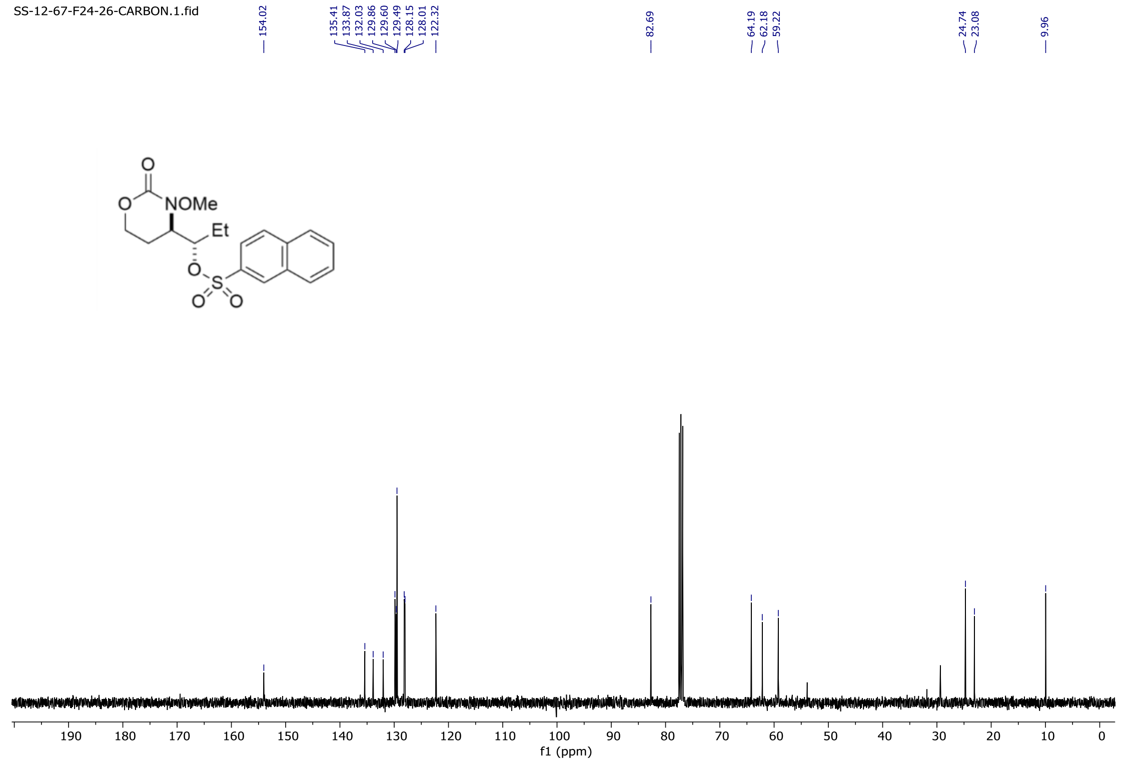

Compound 26 ($CDCl_3$, ^1H NMR: 400 MHz, $^{13}C\{^1H\}$ NMR: 101 MHz)

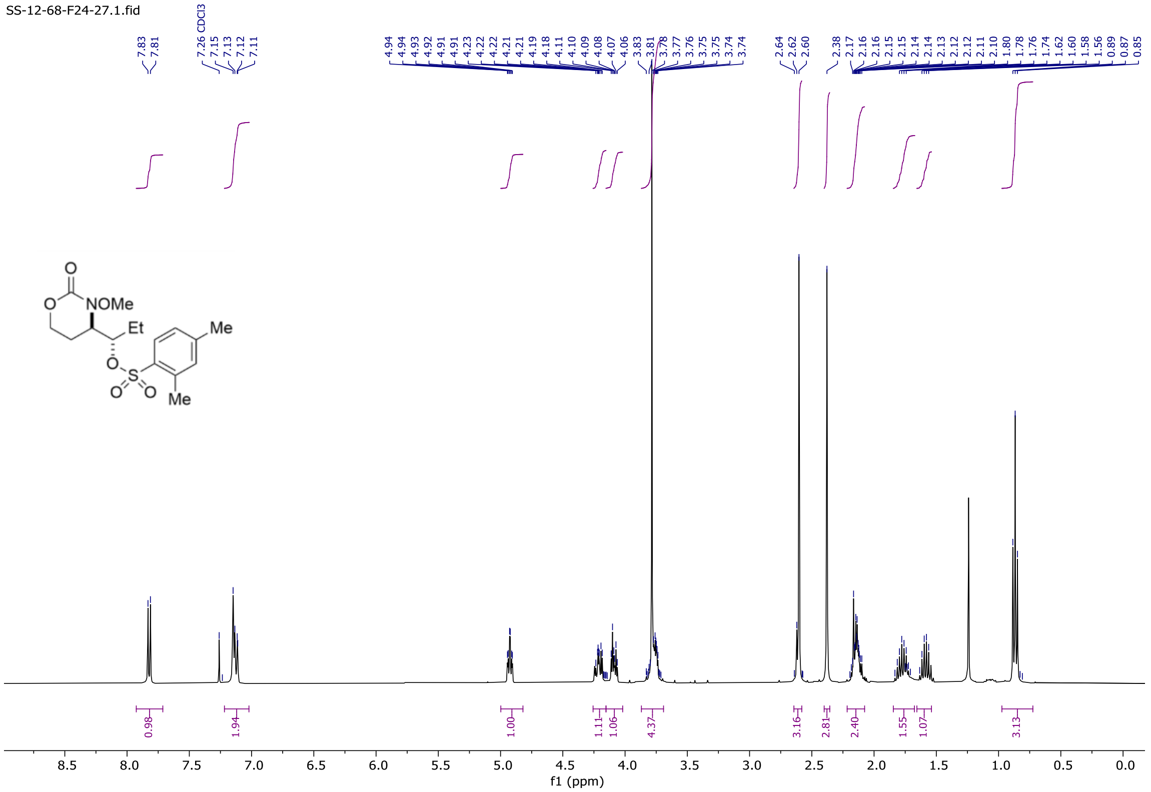

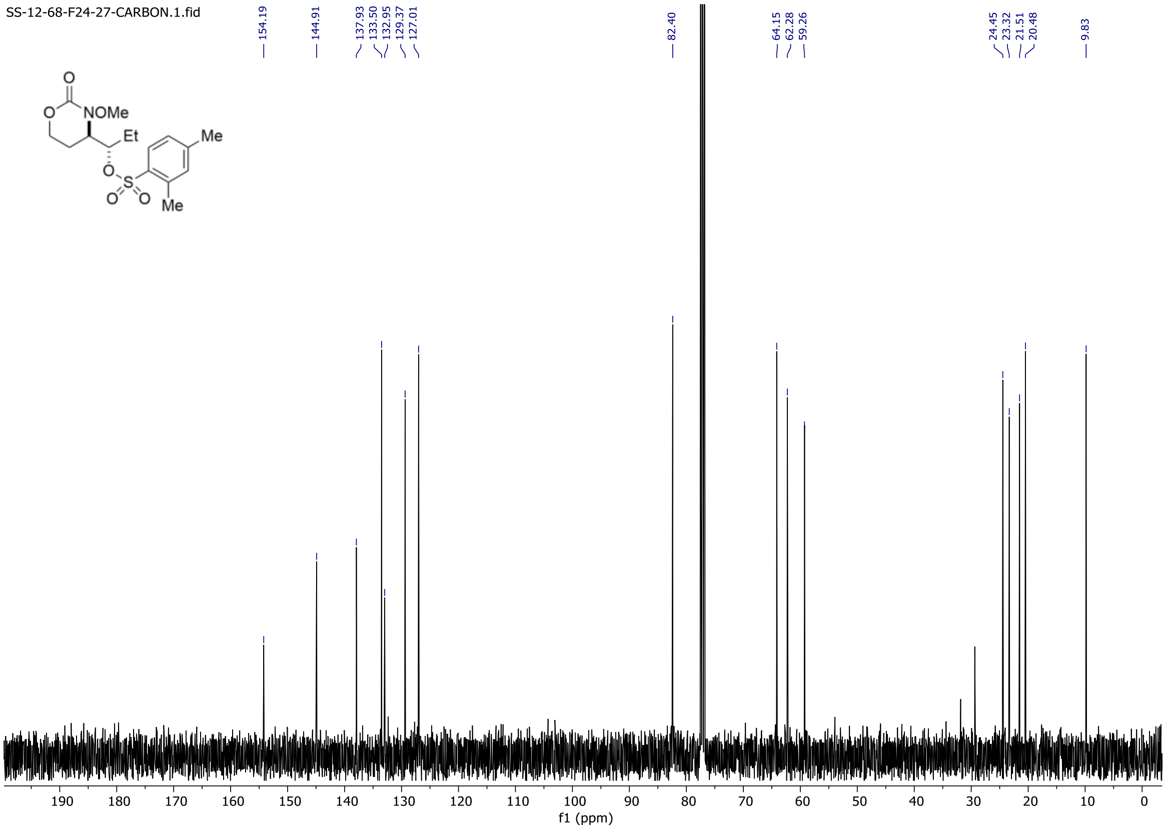

Compound 27 ($CDCl_3$, ^{1}H NMR: 400 MHz, $^{13}C\{^{1}H\}$ NMR: 101 MHz)

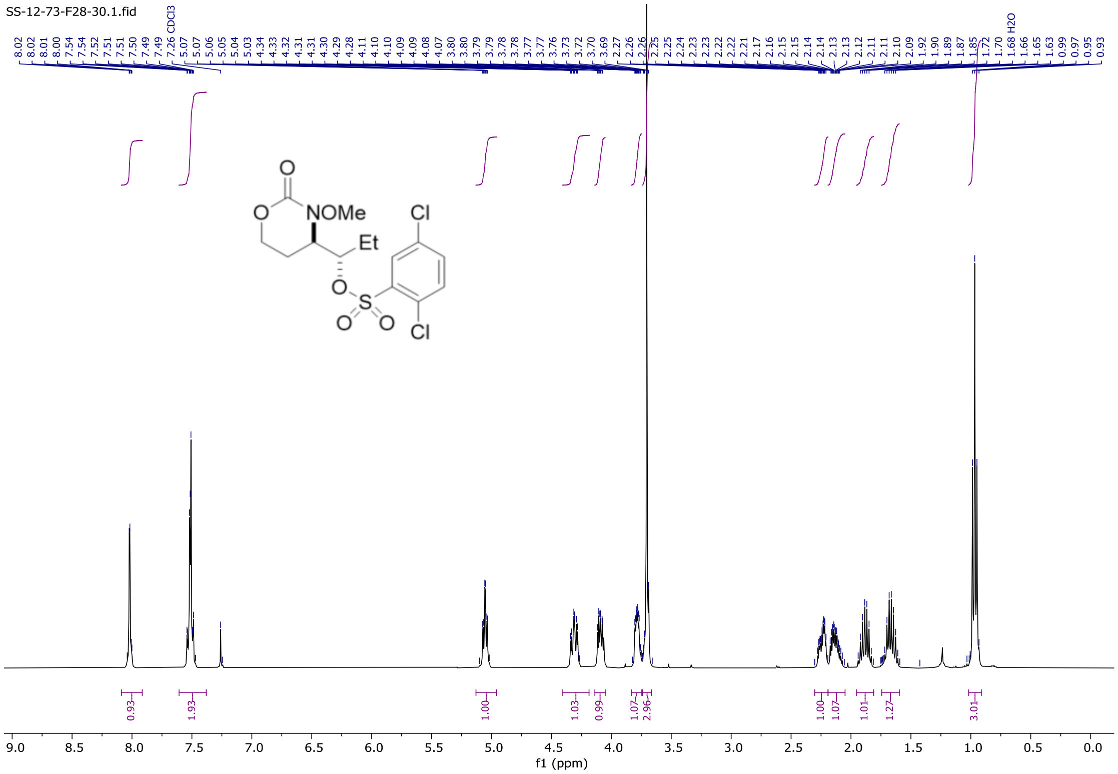

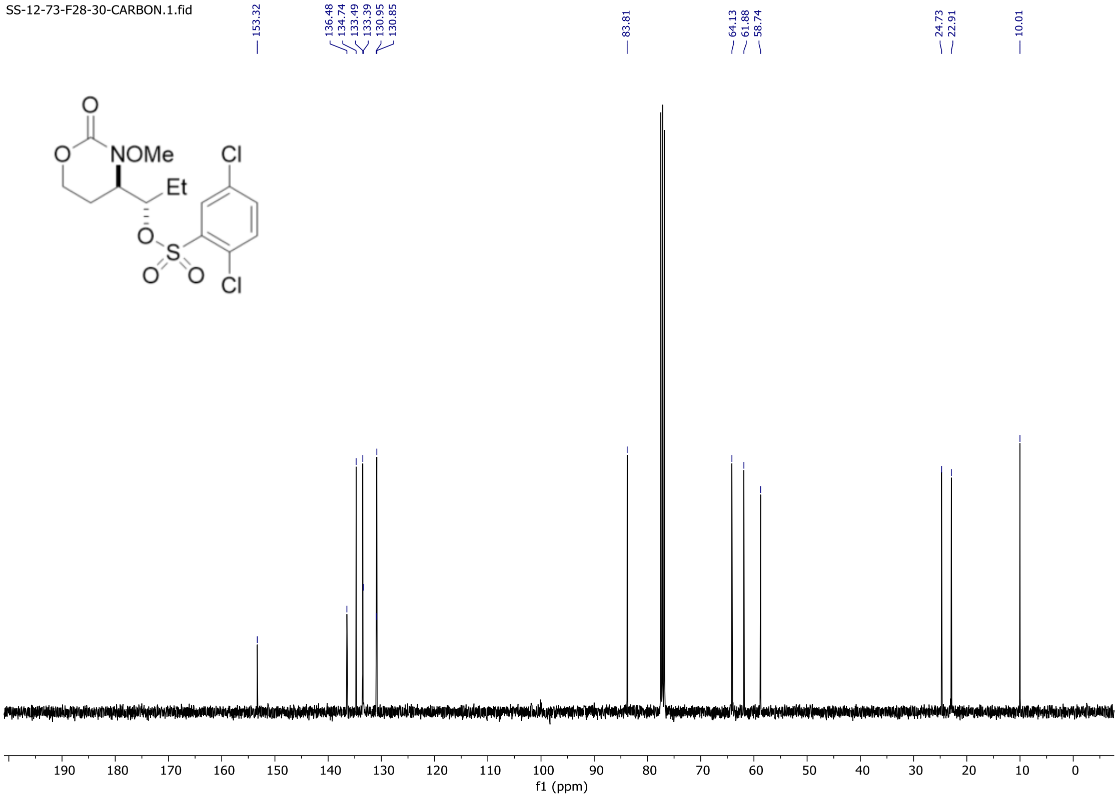

Compound 28 ($CDCl_3$, ^{1}H NMR: 600 MHz, $^{13}C\{^{1}H\}$ NMR: 101 MHz)

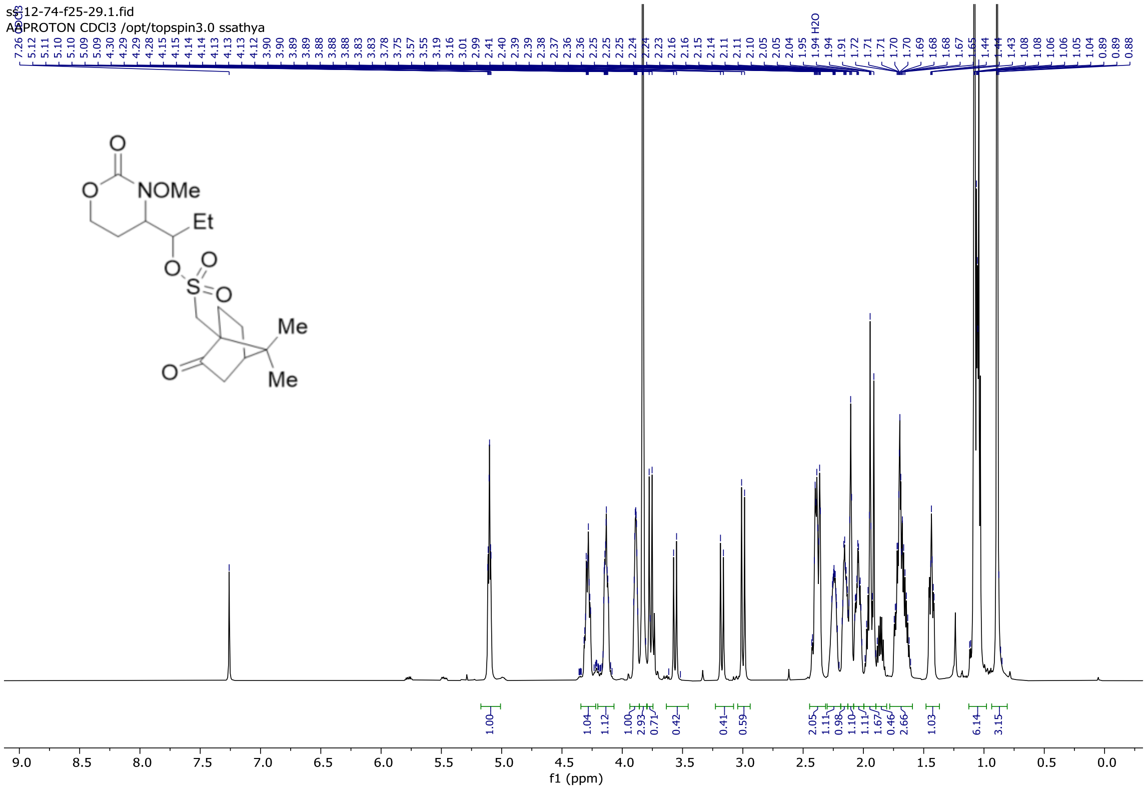

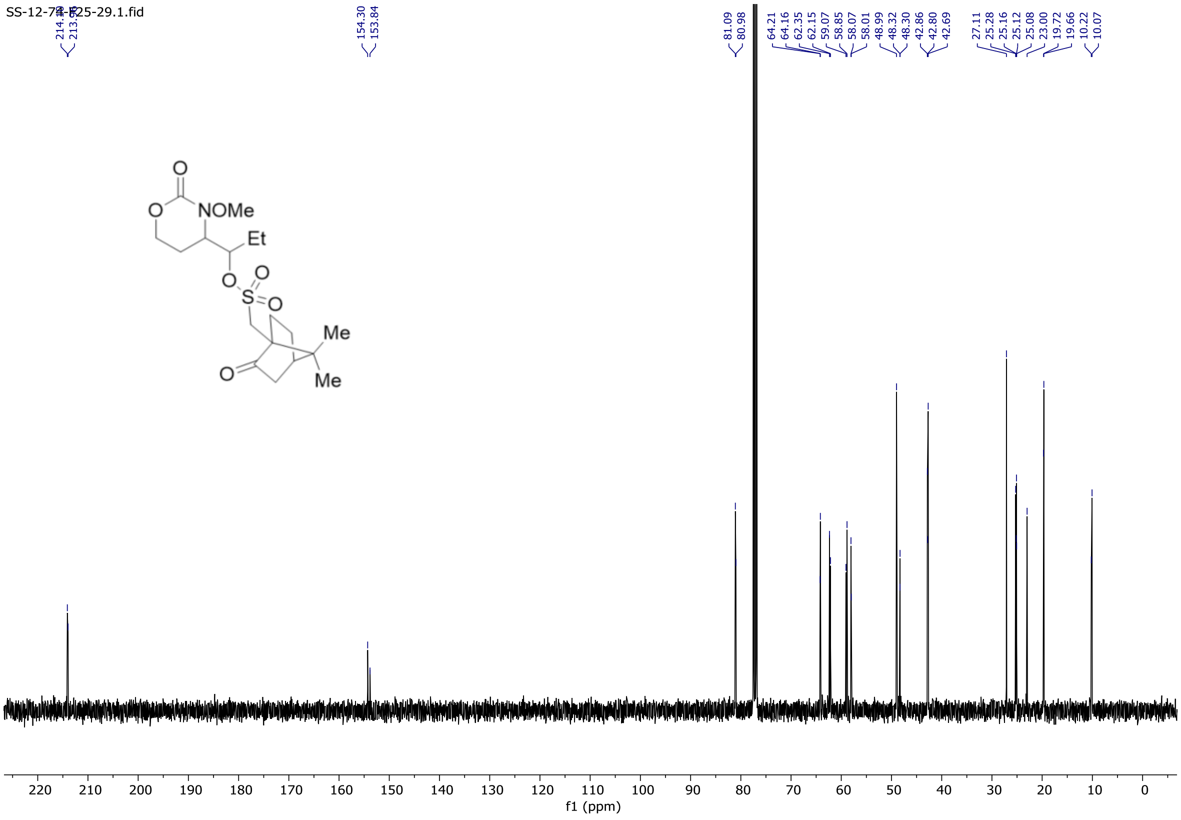

Compound 30 (CDCl₃, ^{1}H NMR: 400 MHz, ^{13}C{^{1}H} NMR: 101 MHz)

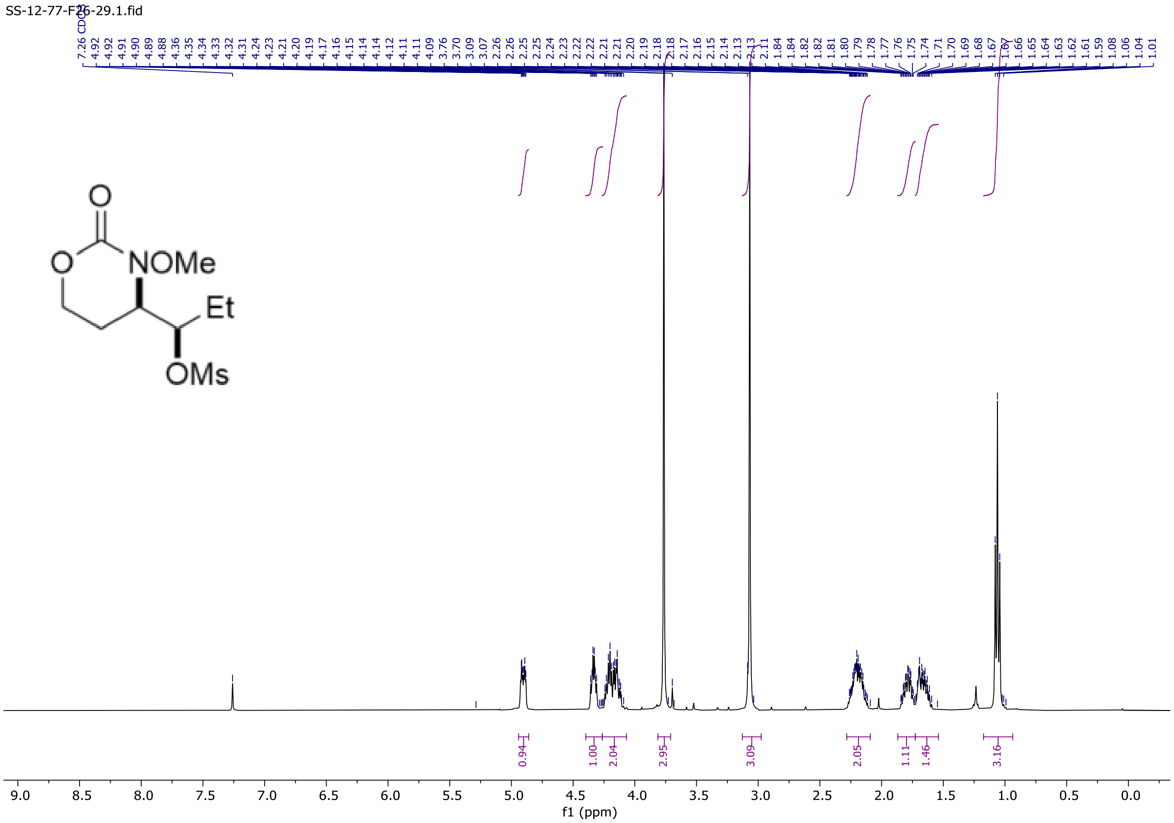

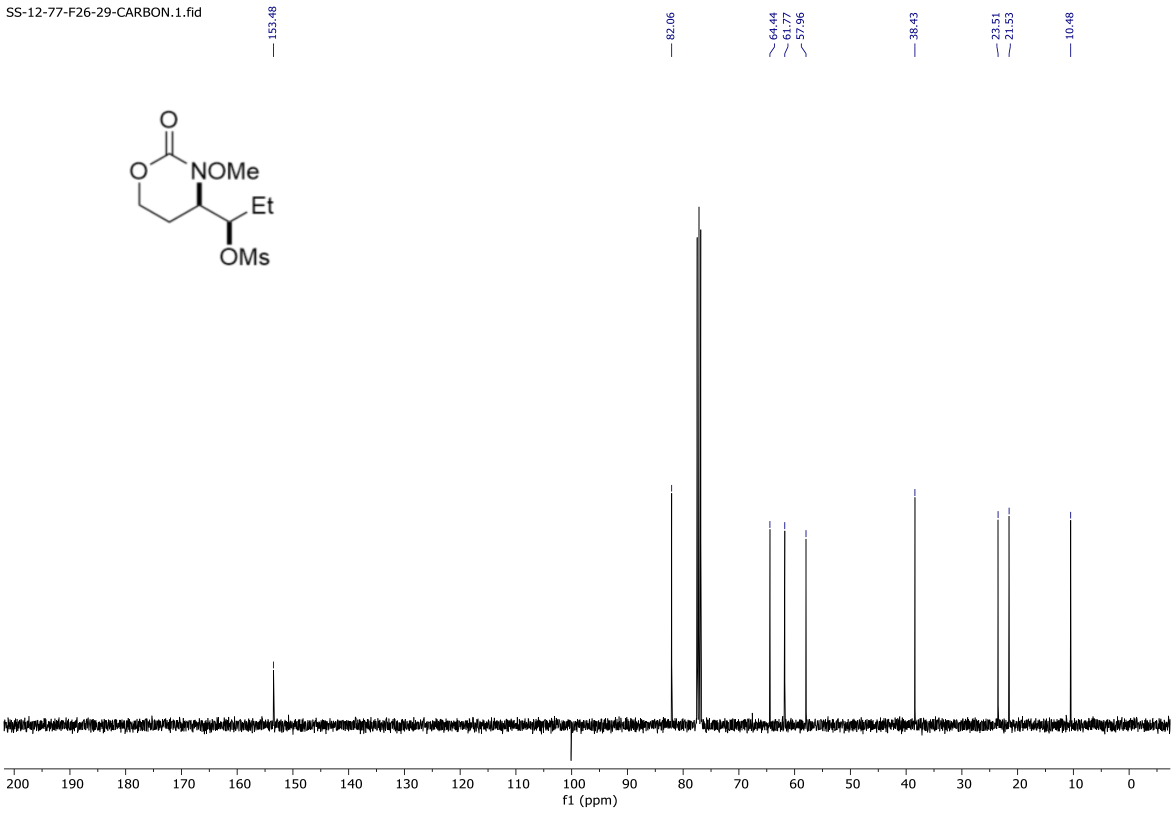

Compound 32 ($CDCl_3$, ^{1}H NMR: 600 MHz, $^{13}C\{^{1}H\}$ NMR: 101 MHz)

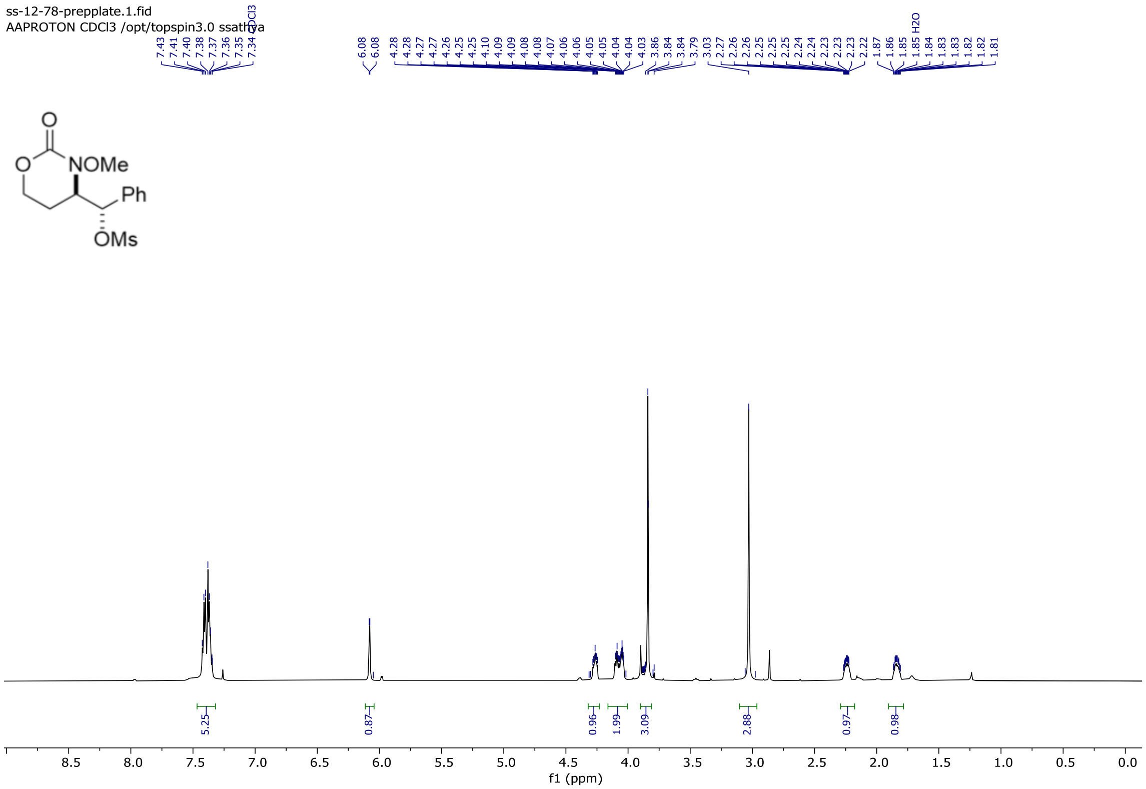

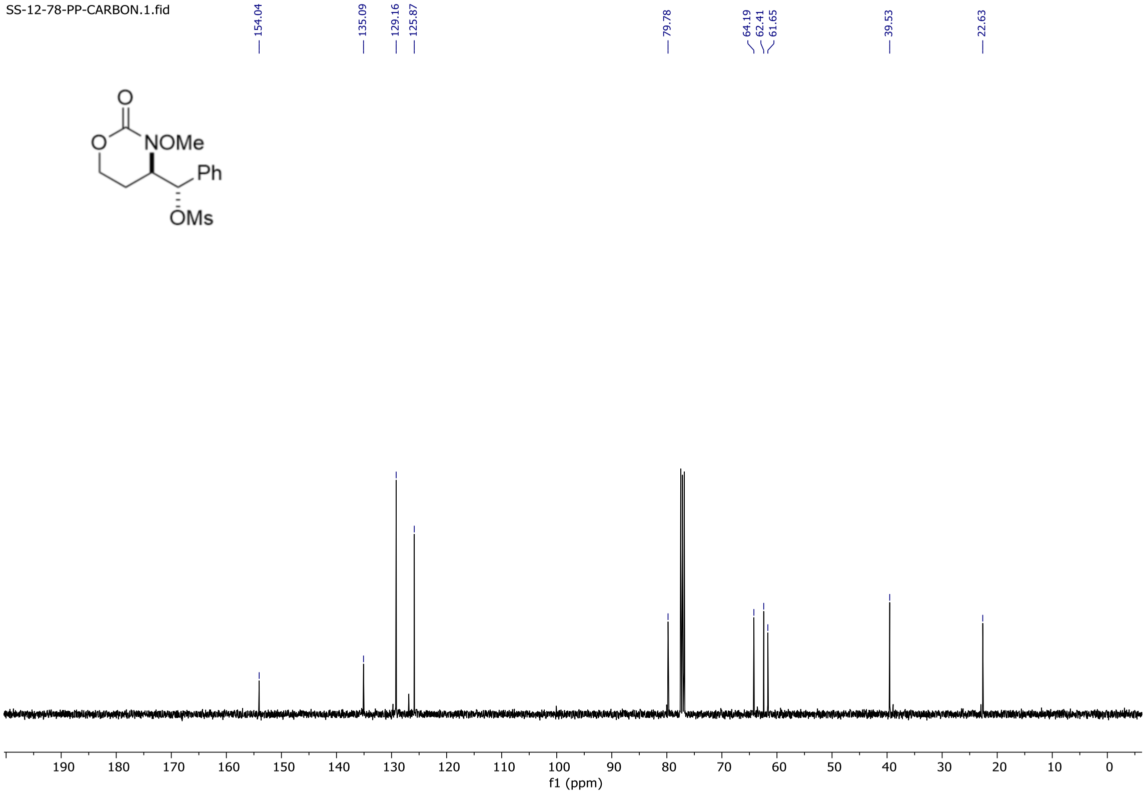

Compound 34 (CDCl₃, ^{1}H NMR: 600 MHz, ^{13}C{^{1}H} NMR: 101 MHz)

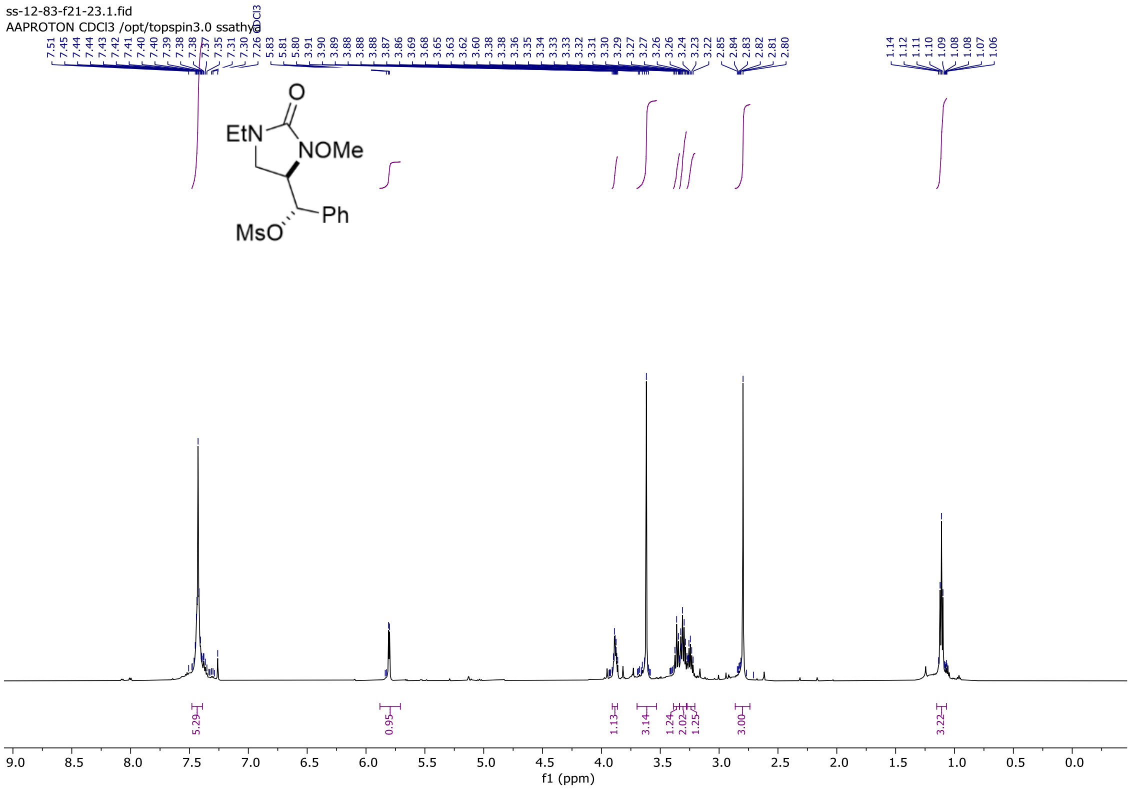

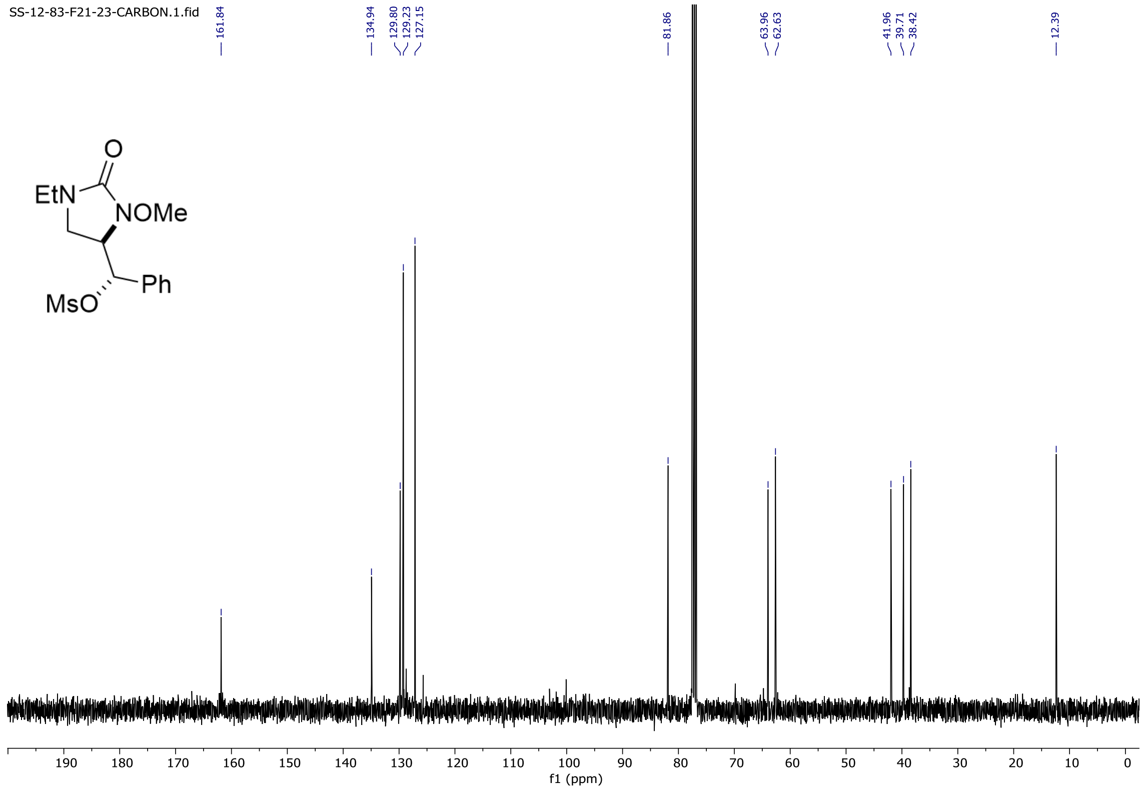

Compound 36 ($CDCl_3$, ^1H NMR: 400 MHz, $^{13}C\{^1H\}$ NMR: 101 MHz)

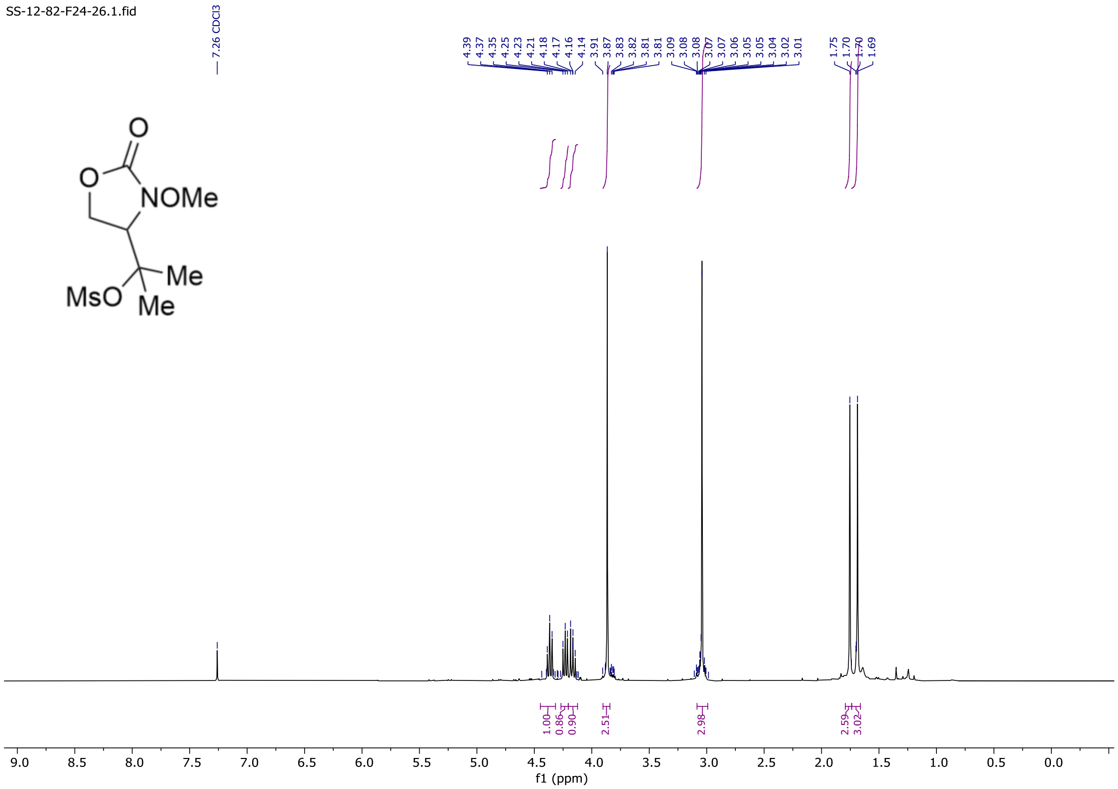

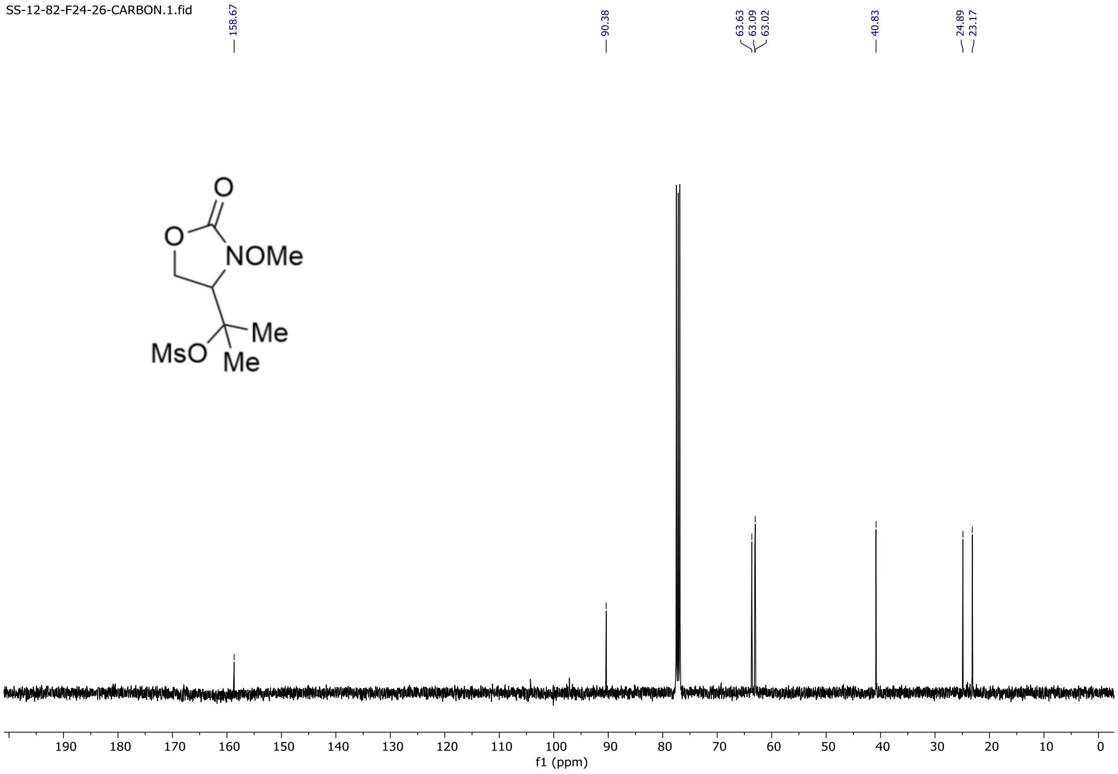

Compound S1 ($CDCl_3$, ^{1}H NMR: 400 MHz, $^{13}C\{^{1}H\}$ NMR: 101 MHz)

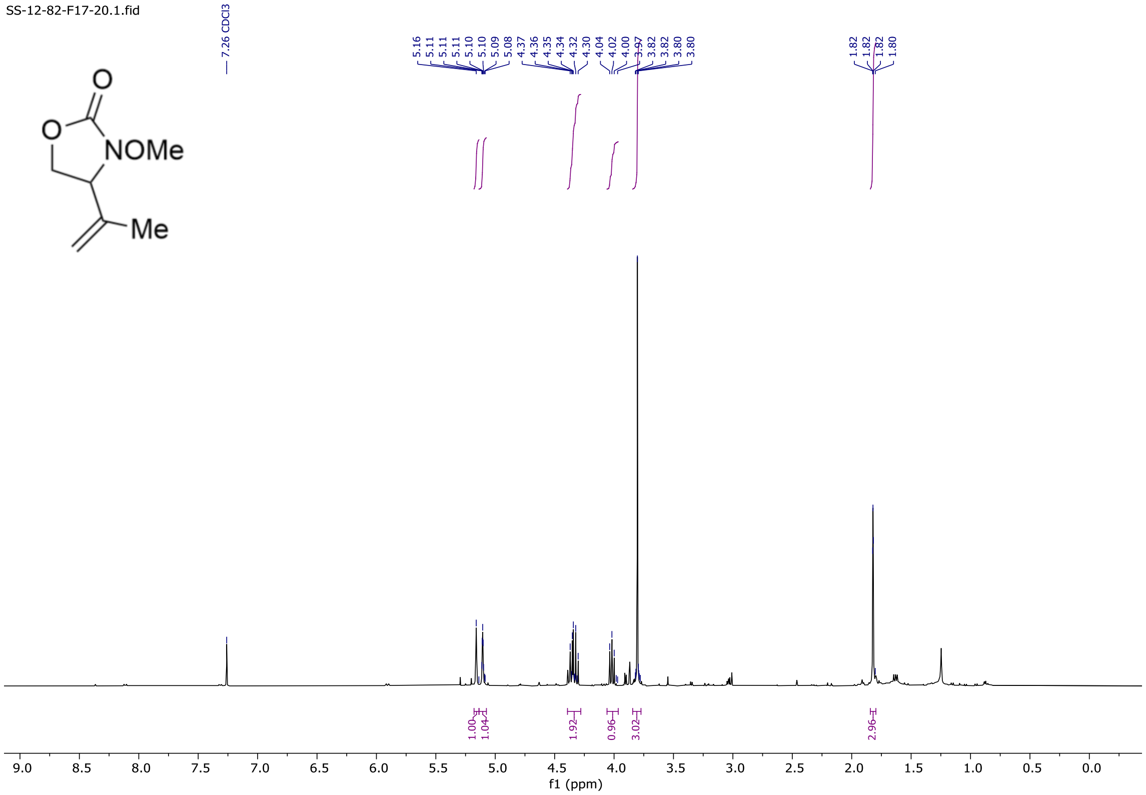

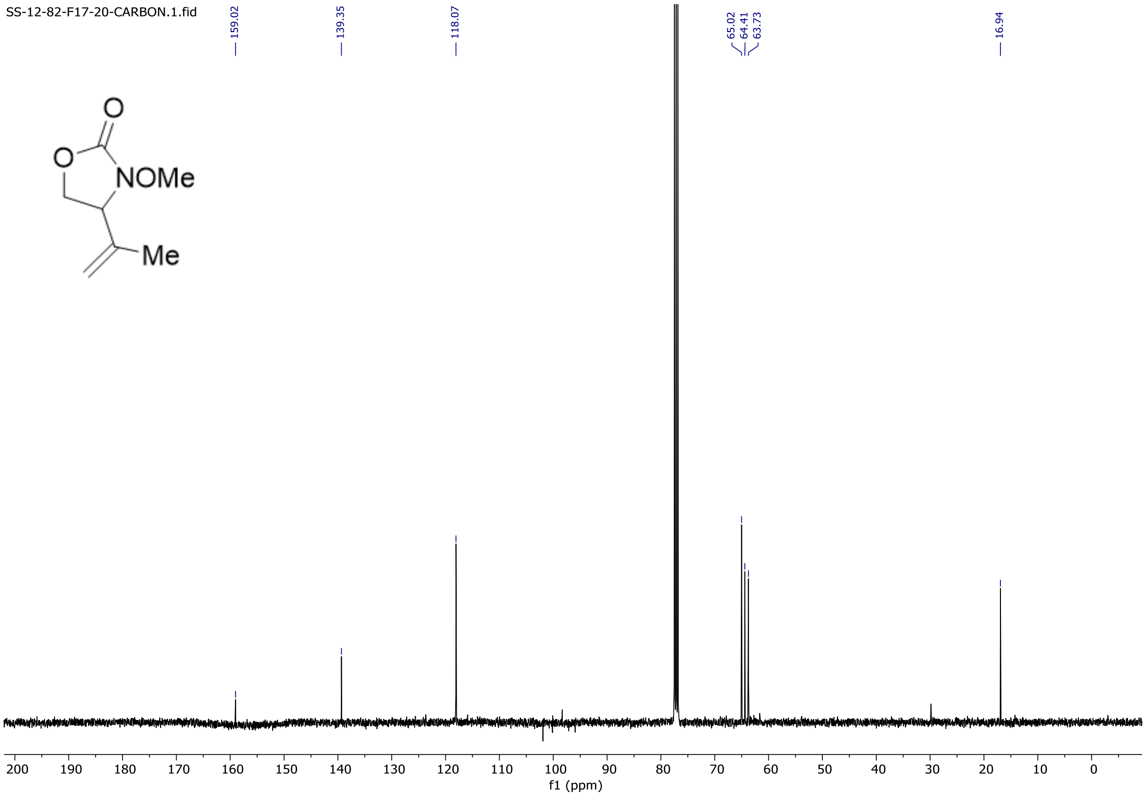

Compound 38 ($CDCl_3$, ^1H NMR: 400 MHz, $^{13}C\{^1H\}$ NMR: 101 MHz)

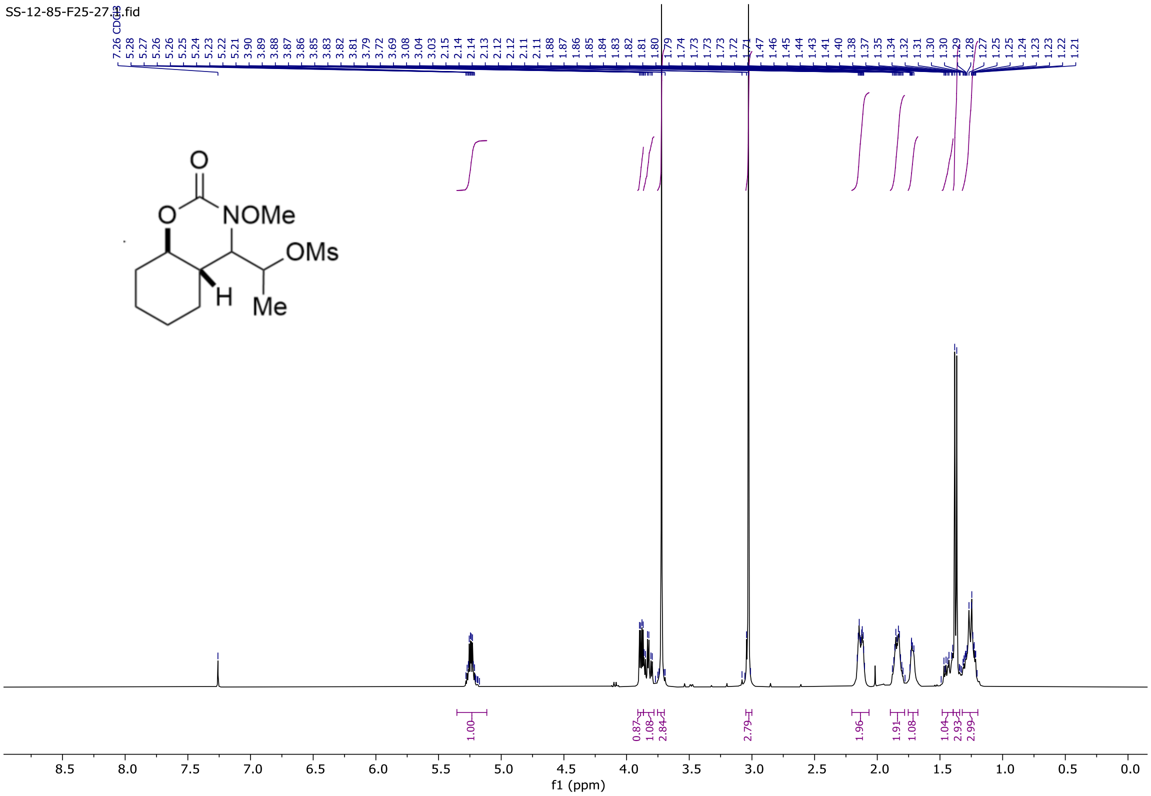

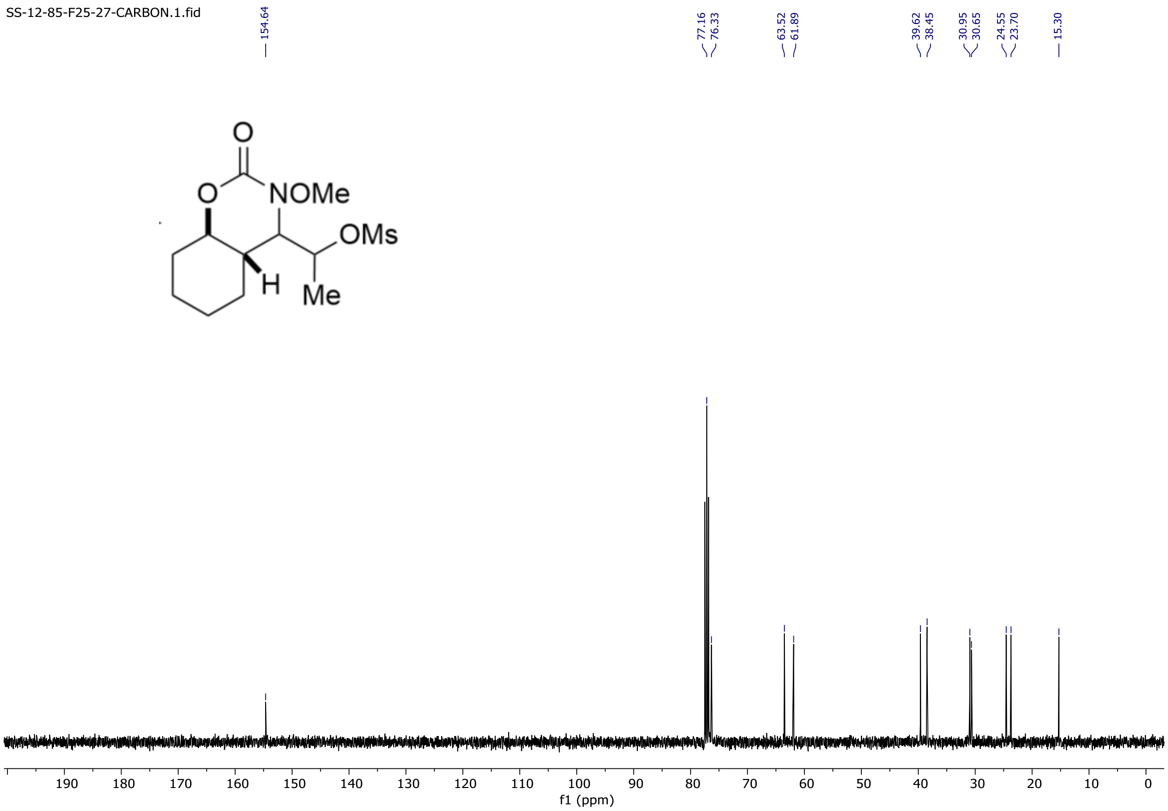

Compound 39 ($CDCl_3$, ^{1}H NMR: 400 MHz, $^{13}C\{^{1}H\}$ NMR: 101 MHz)

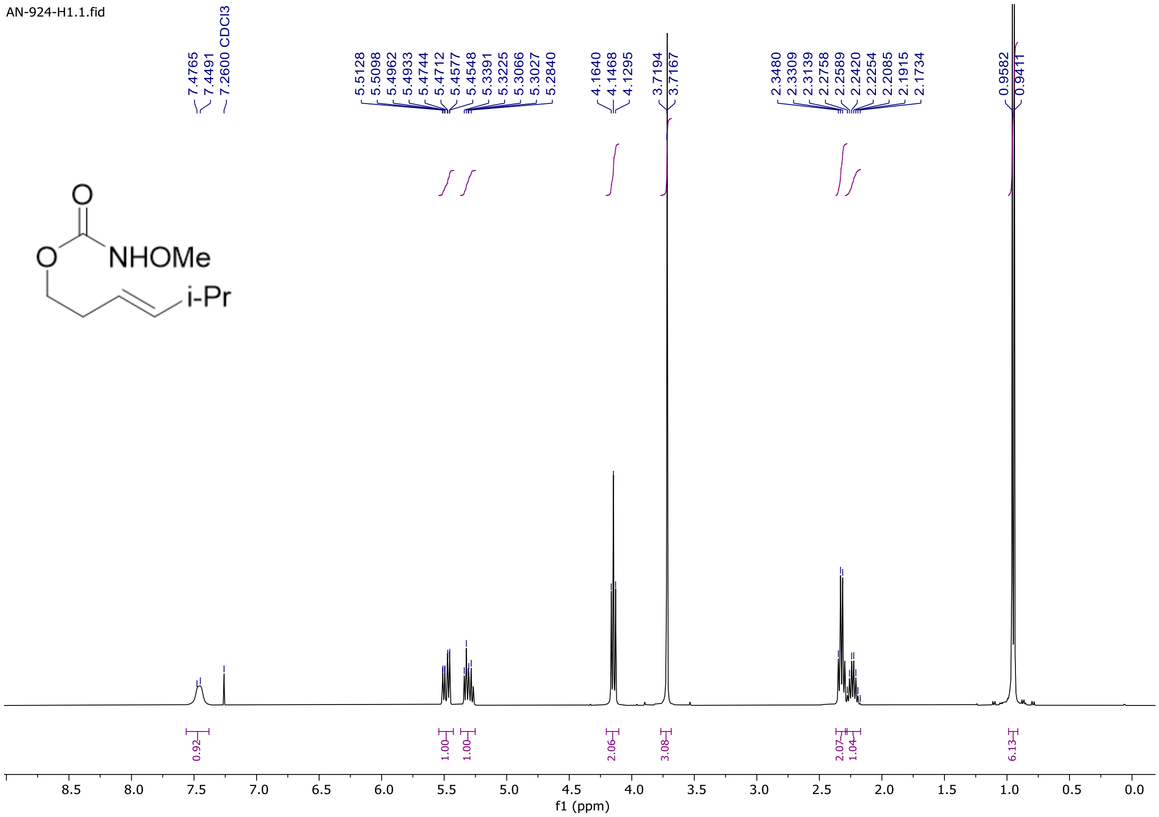

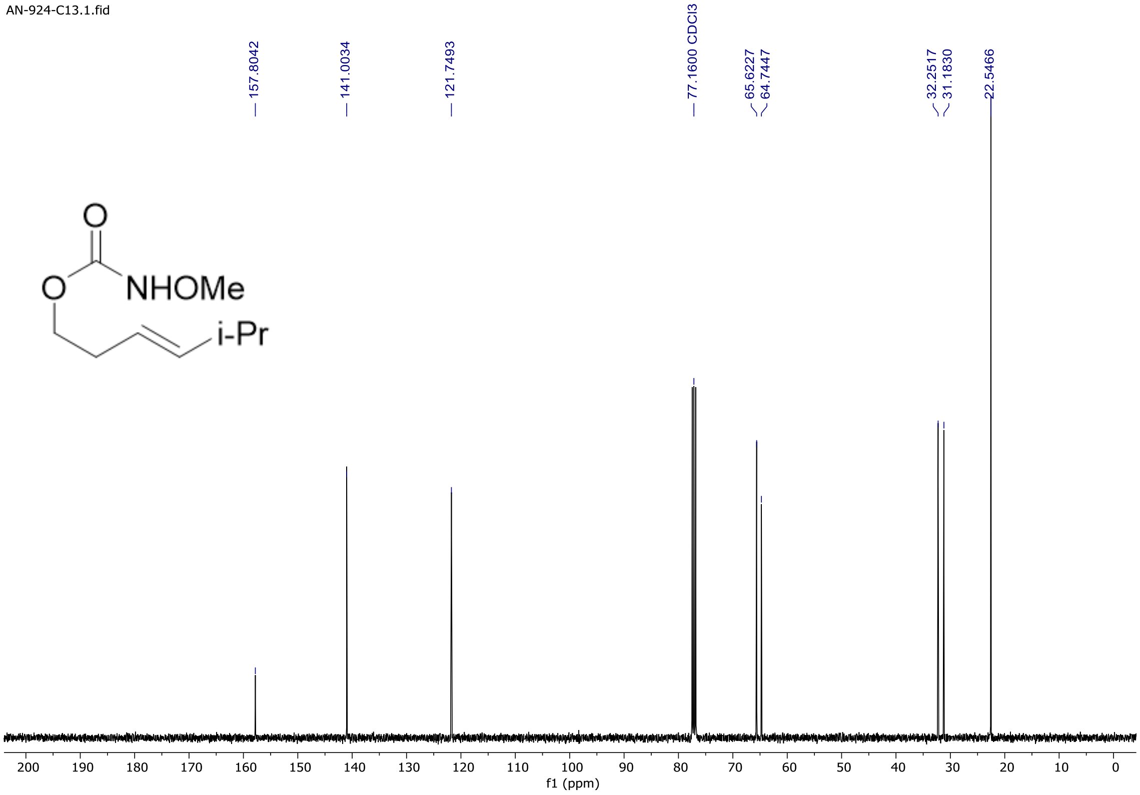

Compound 40 ($CDCl_3$, ^1H NMR: 600 MHz, $^{13}C\{^1H\}$ NMR: 101 MHz)

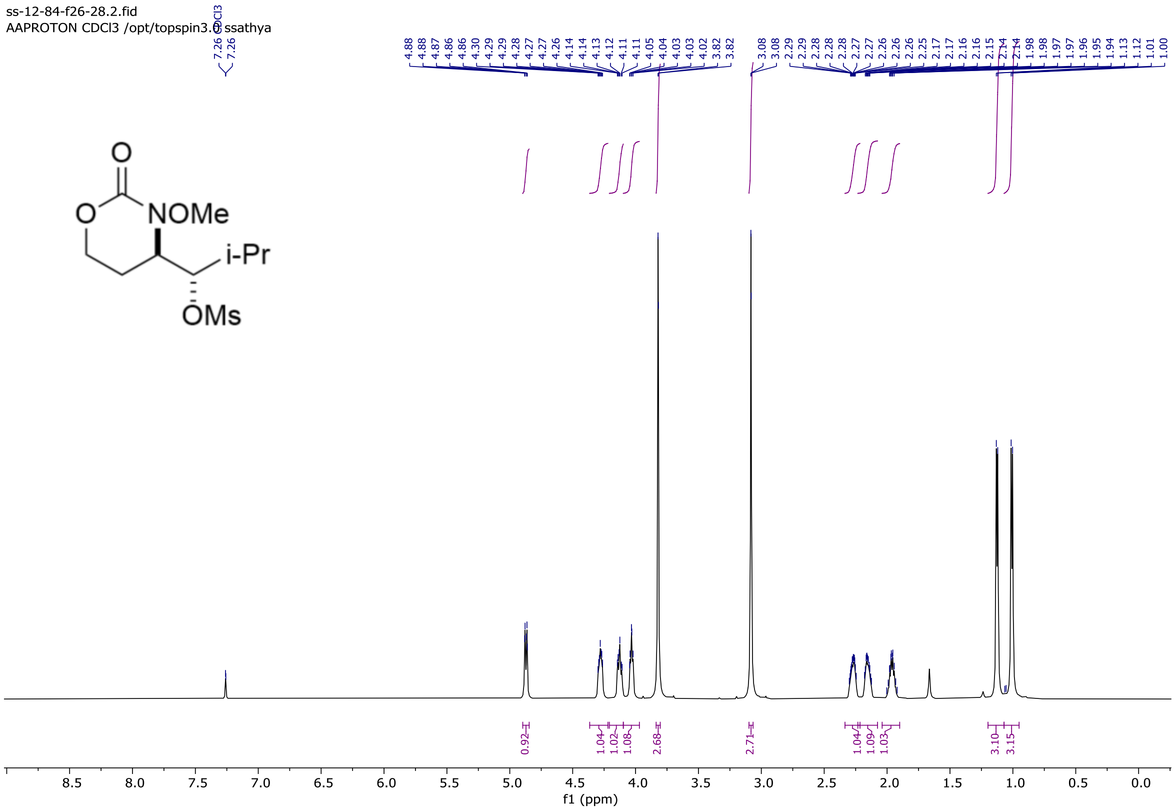

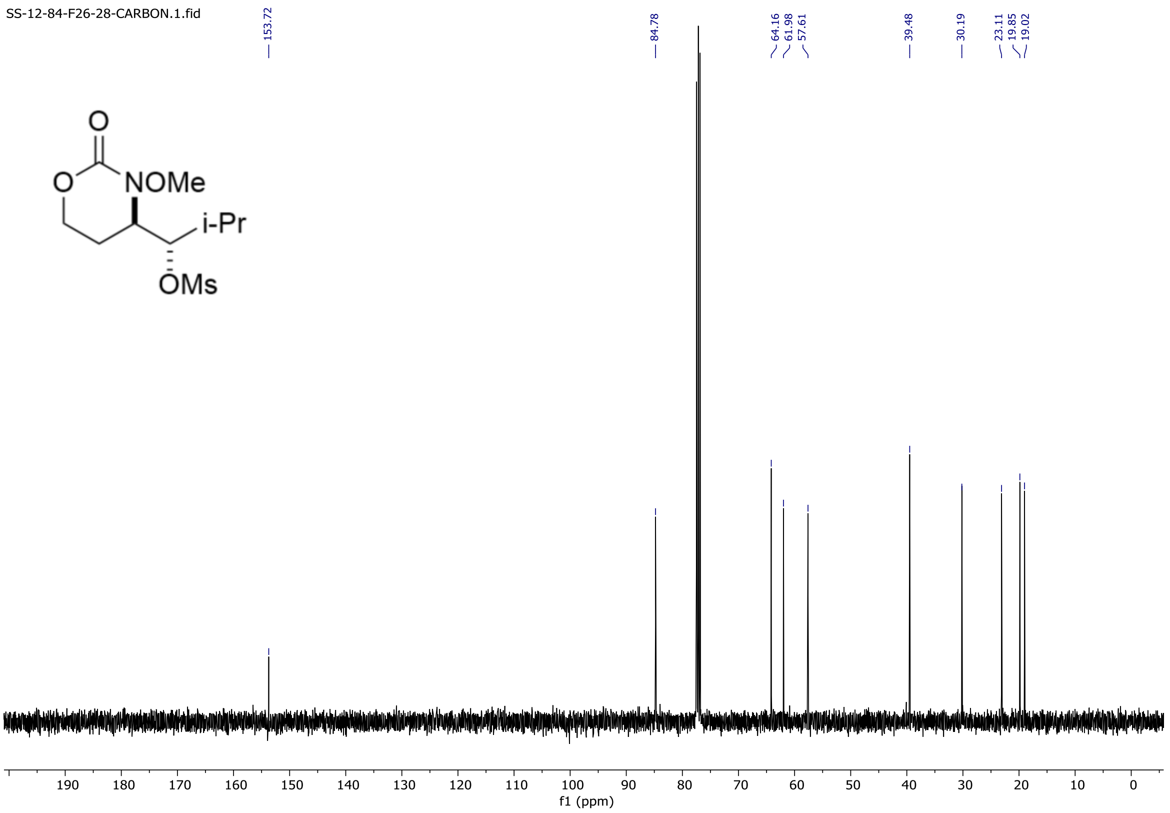

Compound 41 ($CDCl_3$, ^{1}H NMR: 400 MHz, $^{13}C\{^{1}H\}$ NMR: 101 MHz)

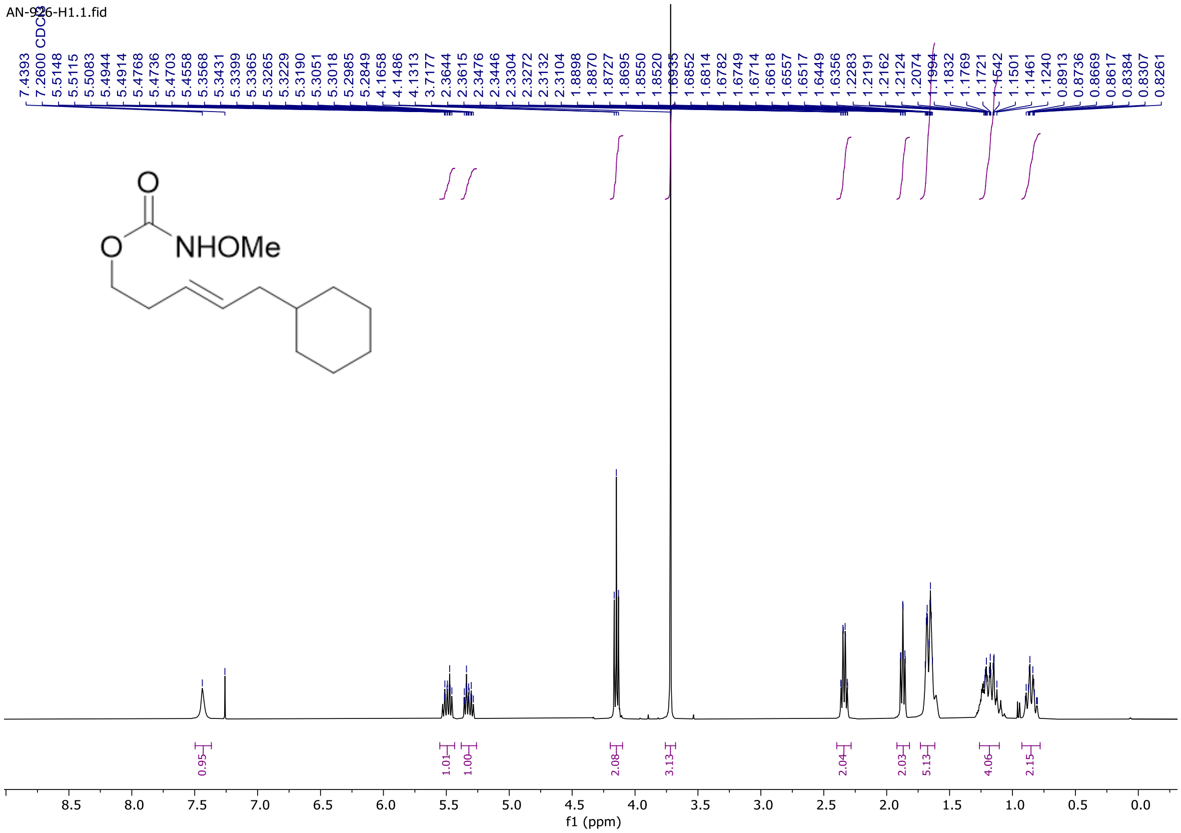

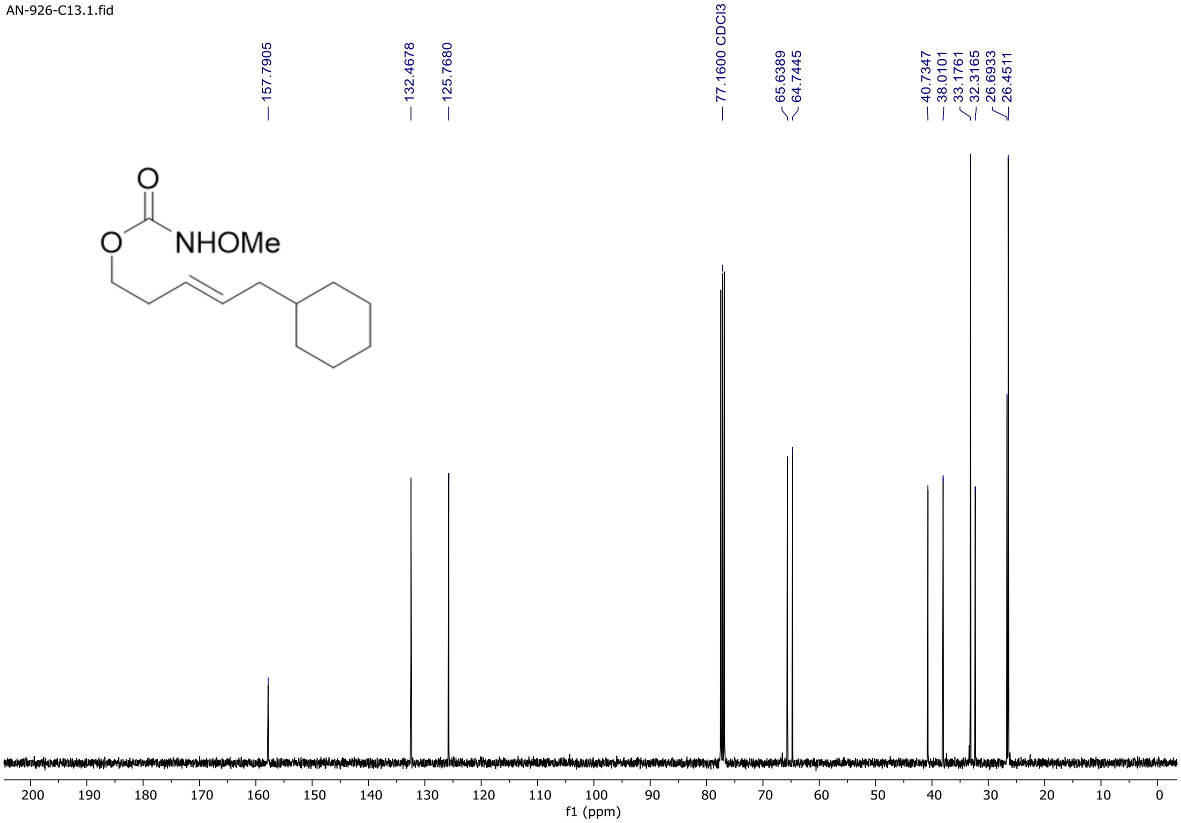

Compound 42 ($CDCl_3$, ^{1}H NMR: 400 MHz, $^{13}C\{^{1}H\}$ NMR: 101 MHz)

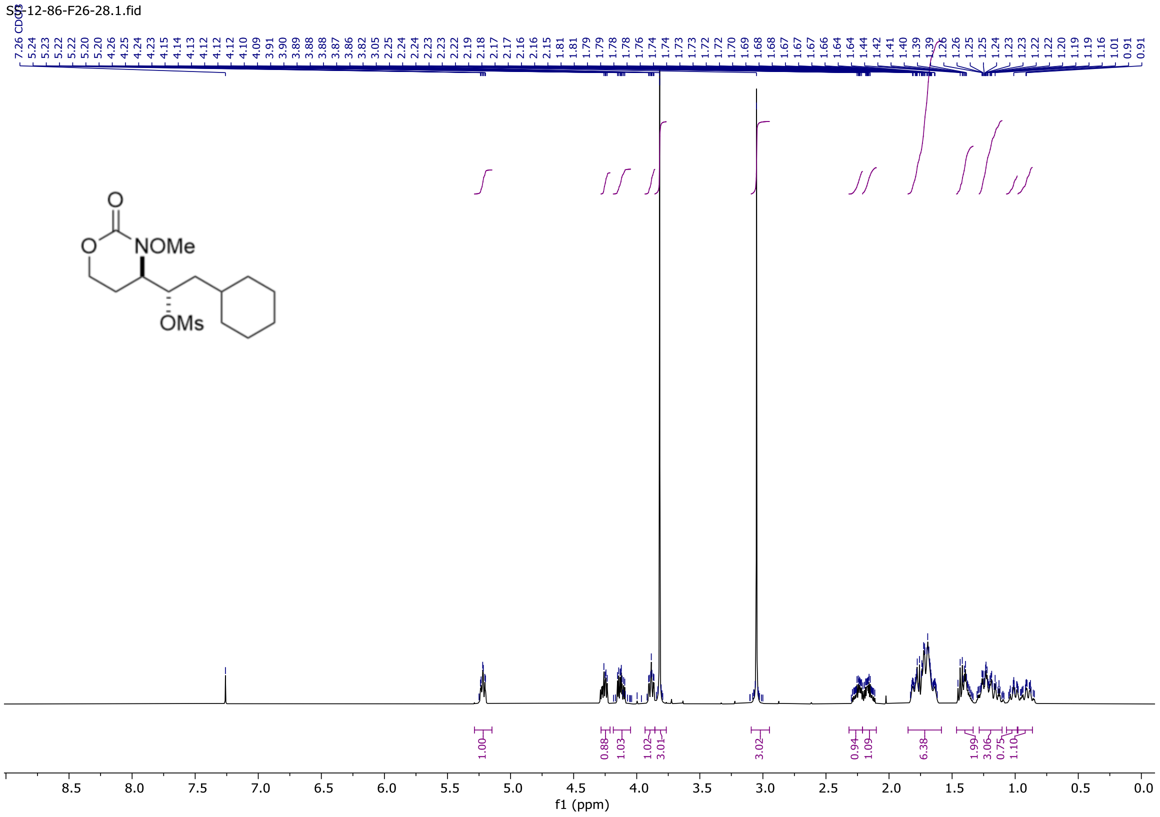

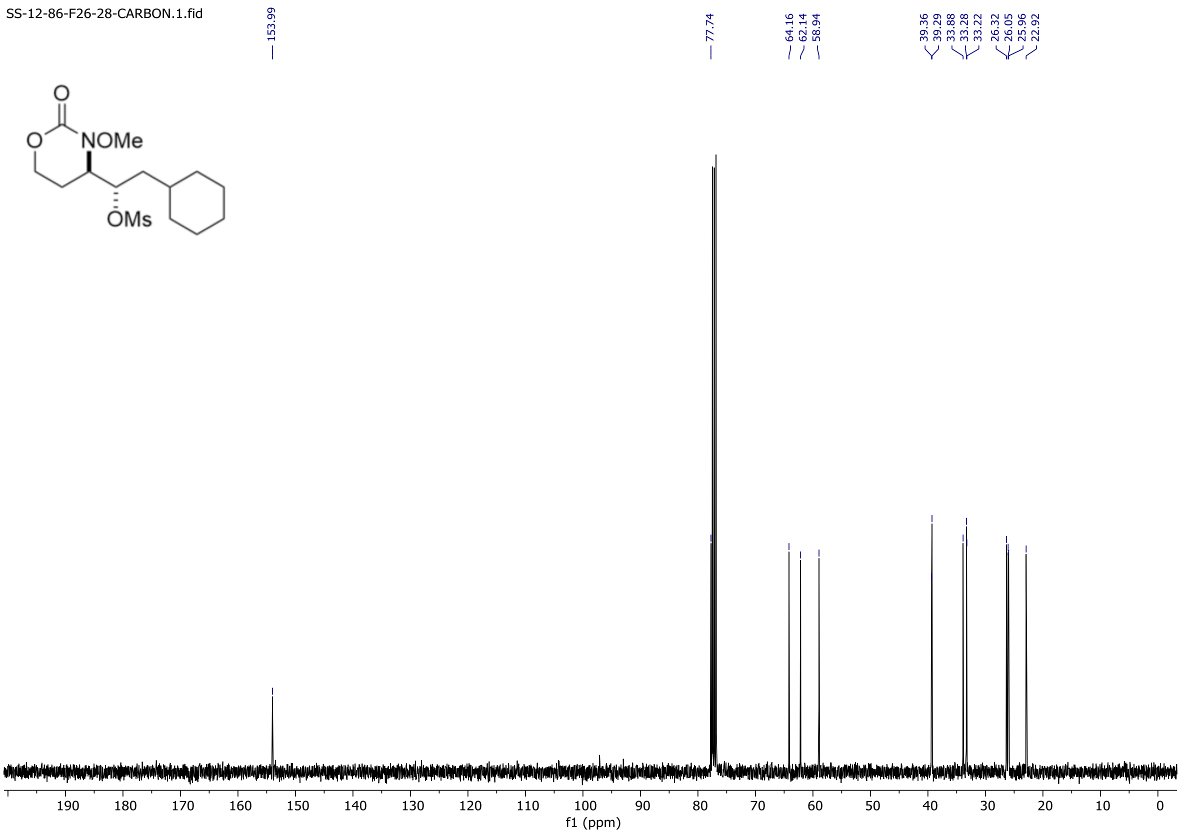

Compound 43 ($CDCl_3$, ^1H NMR: 400 MHz, $^{13}C\{^1H\}$ NMR: 101 MHz)

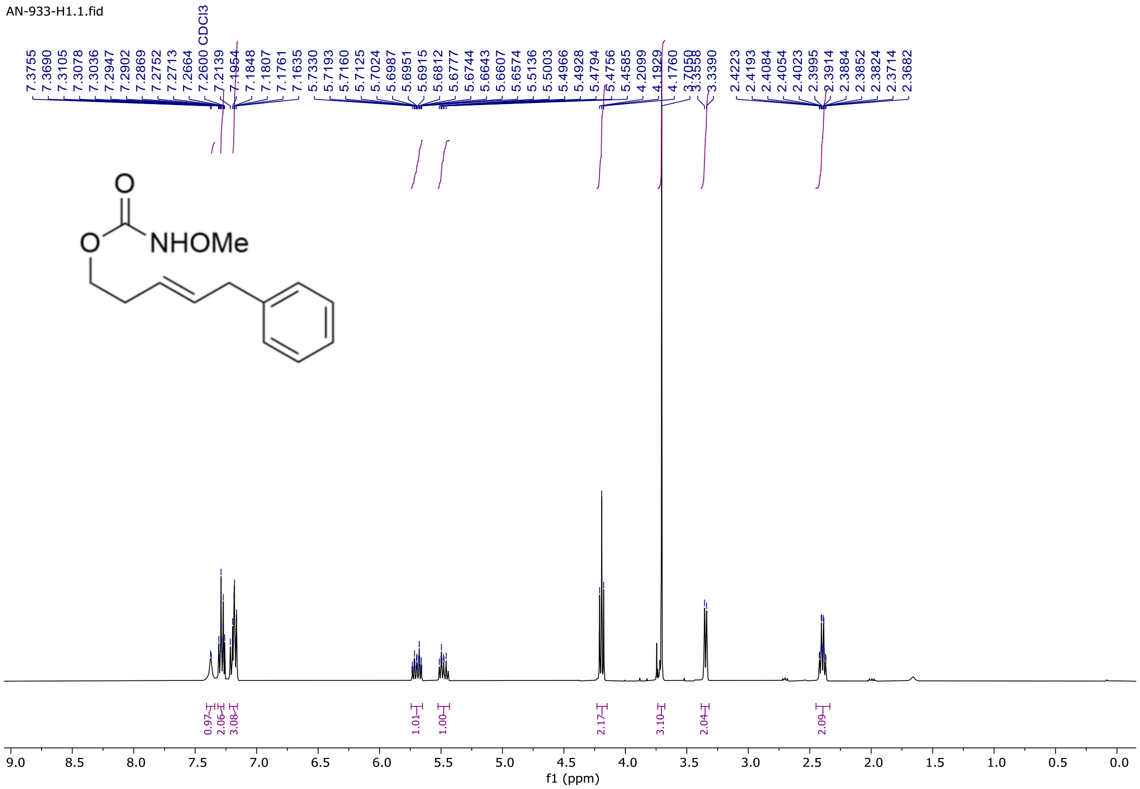

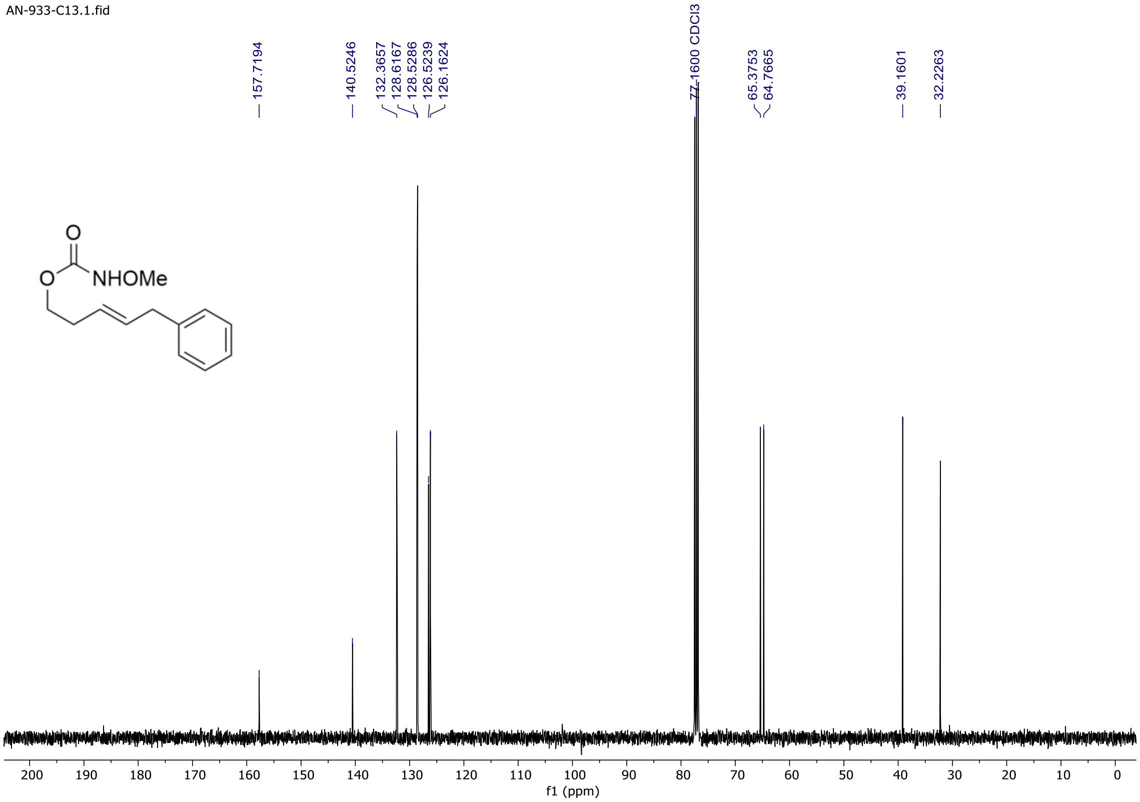

Compound 44 ($CDCl_3$, ^1H NMR: 400 MHz, $^{13}C\{^1H\}$ NMR: 101 MHz)

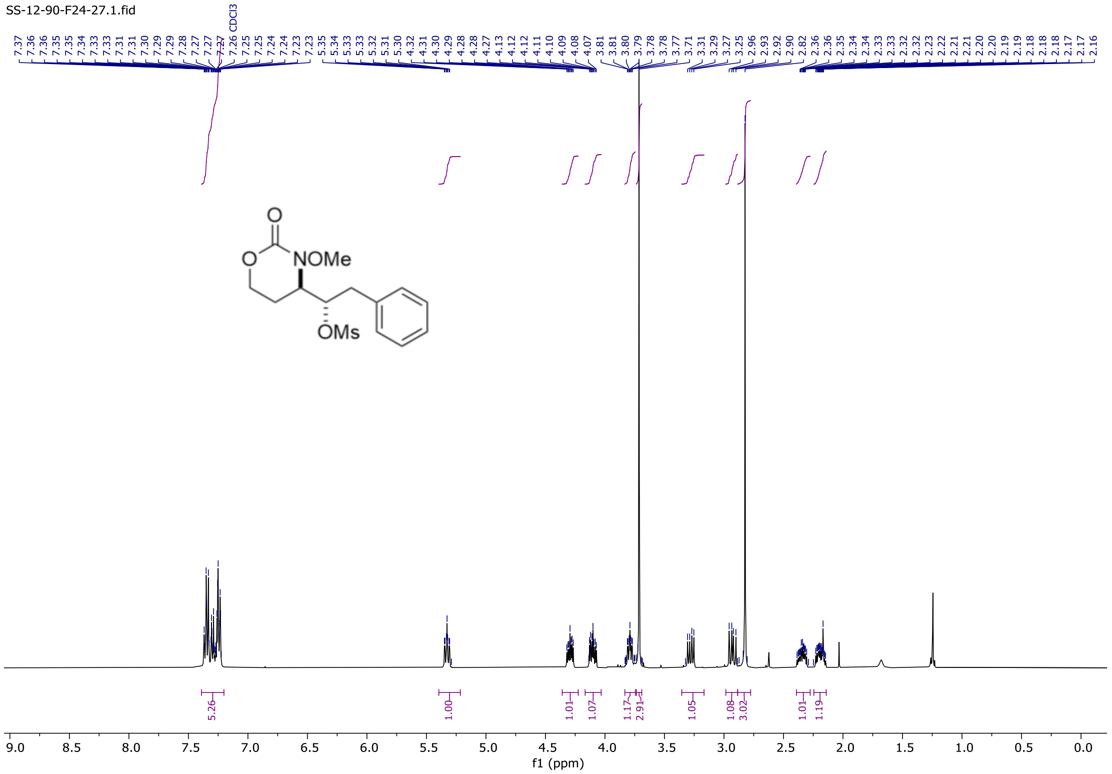

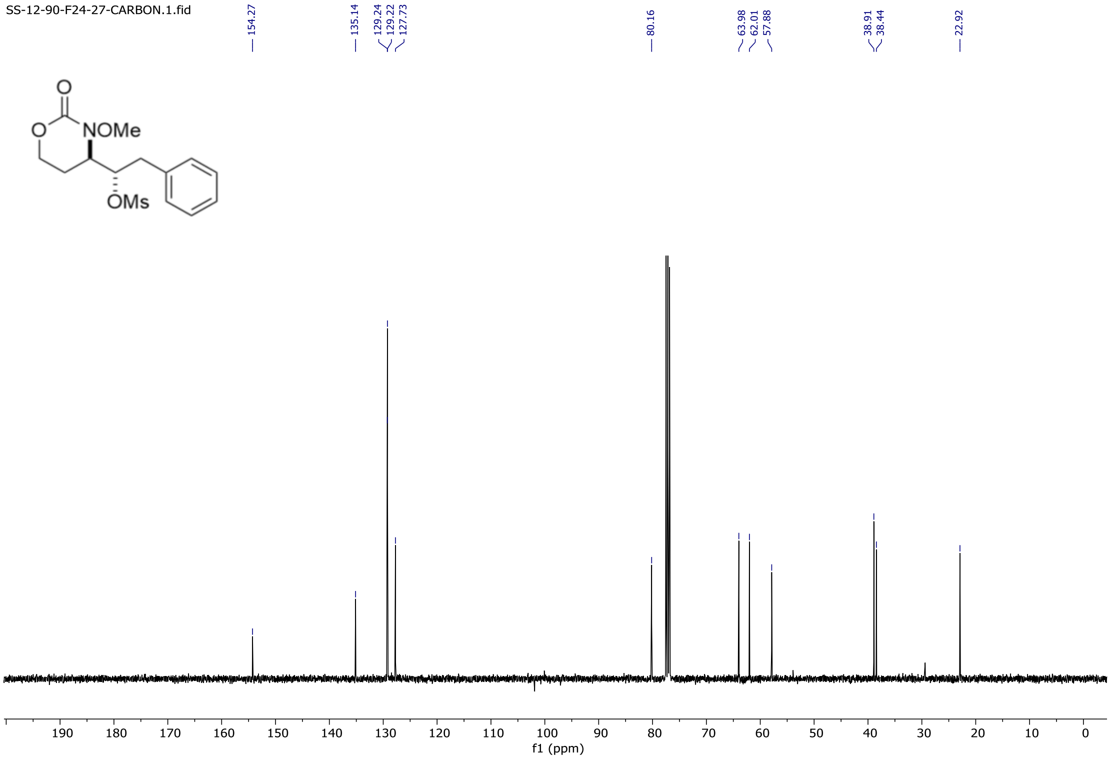

Compound 45 ($CDCl_3$, ^1H NMR: 400 MHz, $^{13}C\{^1H\}$ NMR: 101 MHz)

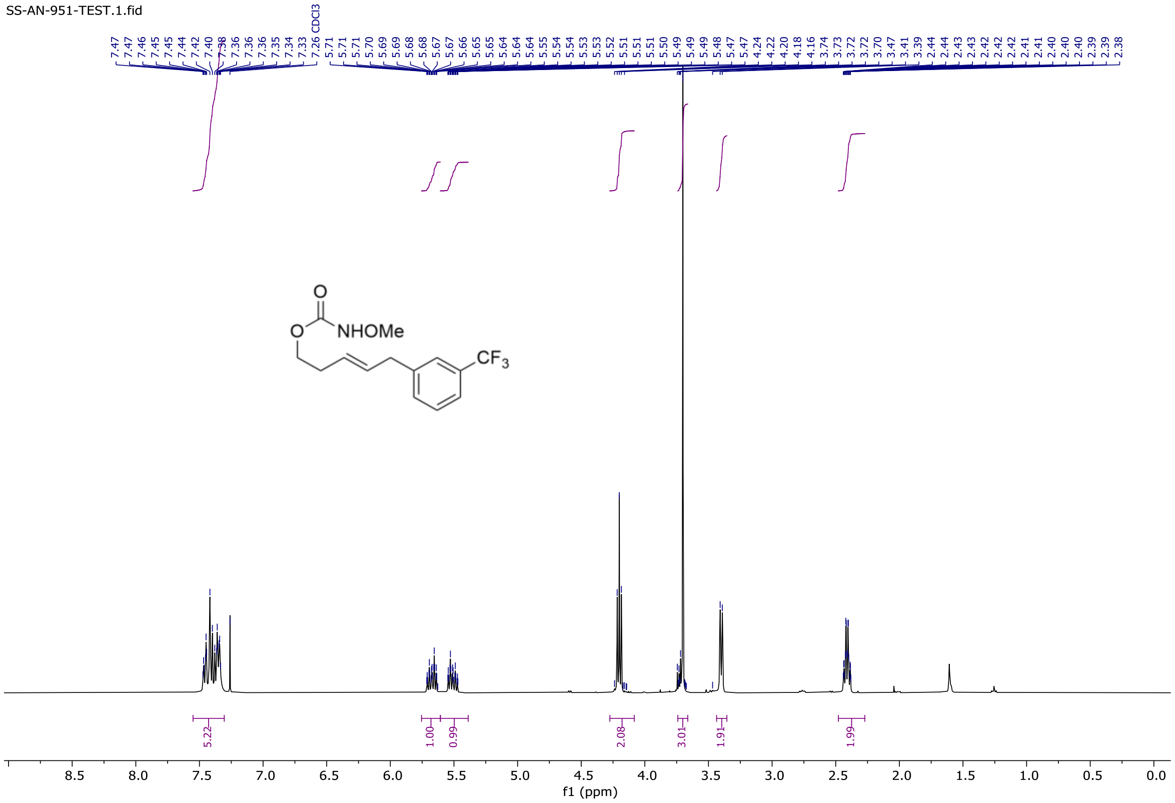

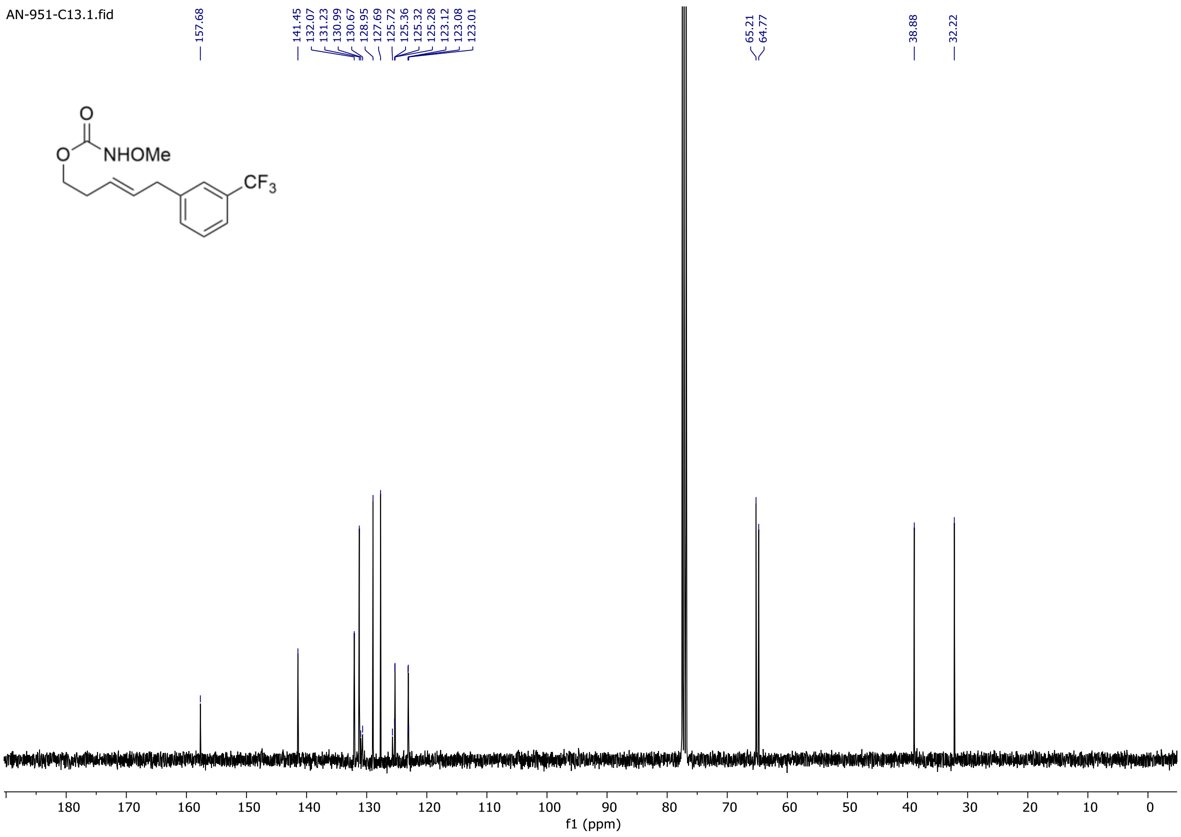

Compound 46 ($CDCl_3$, ^{1}H NMR: 600 MHz, $^{13}C\{^{1}H\}$ NMR: 101 MHz)

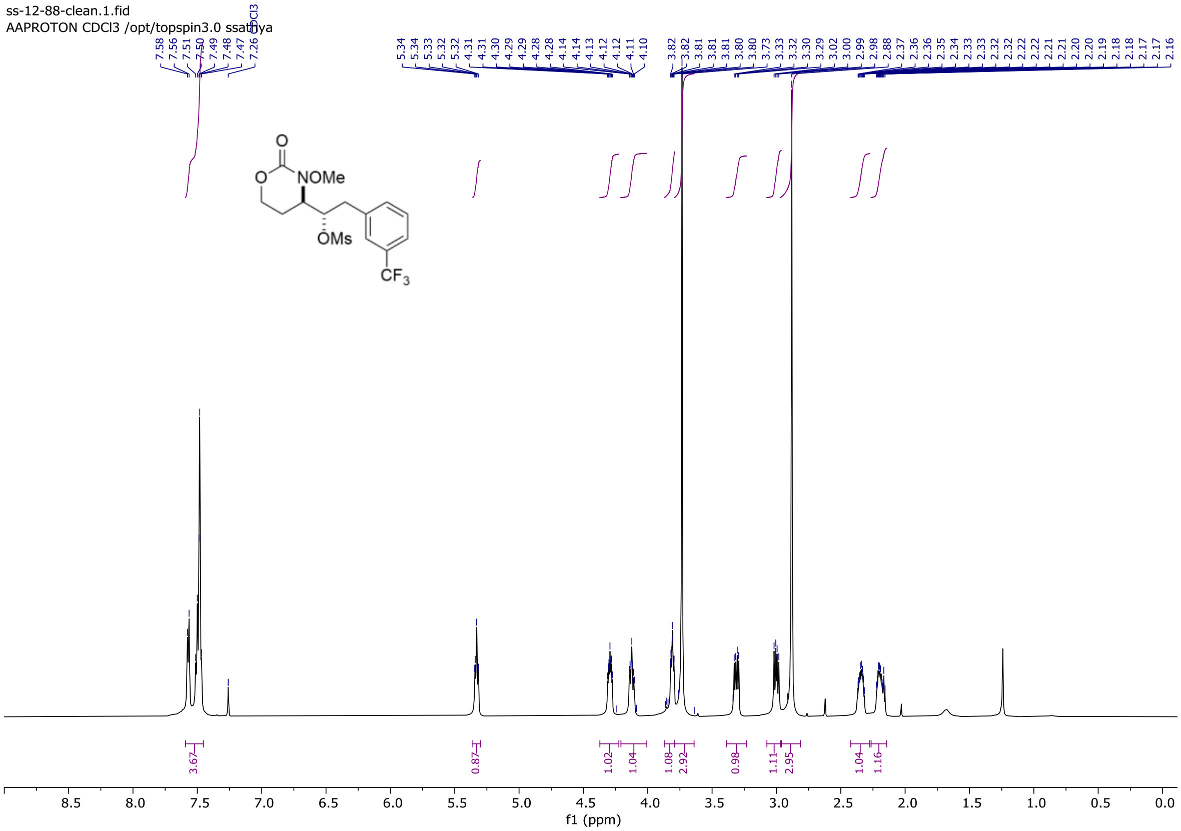

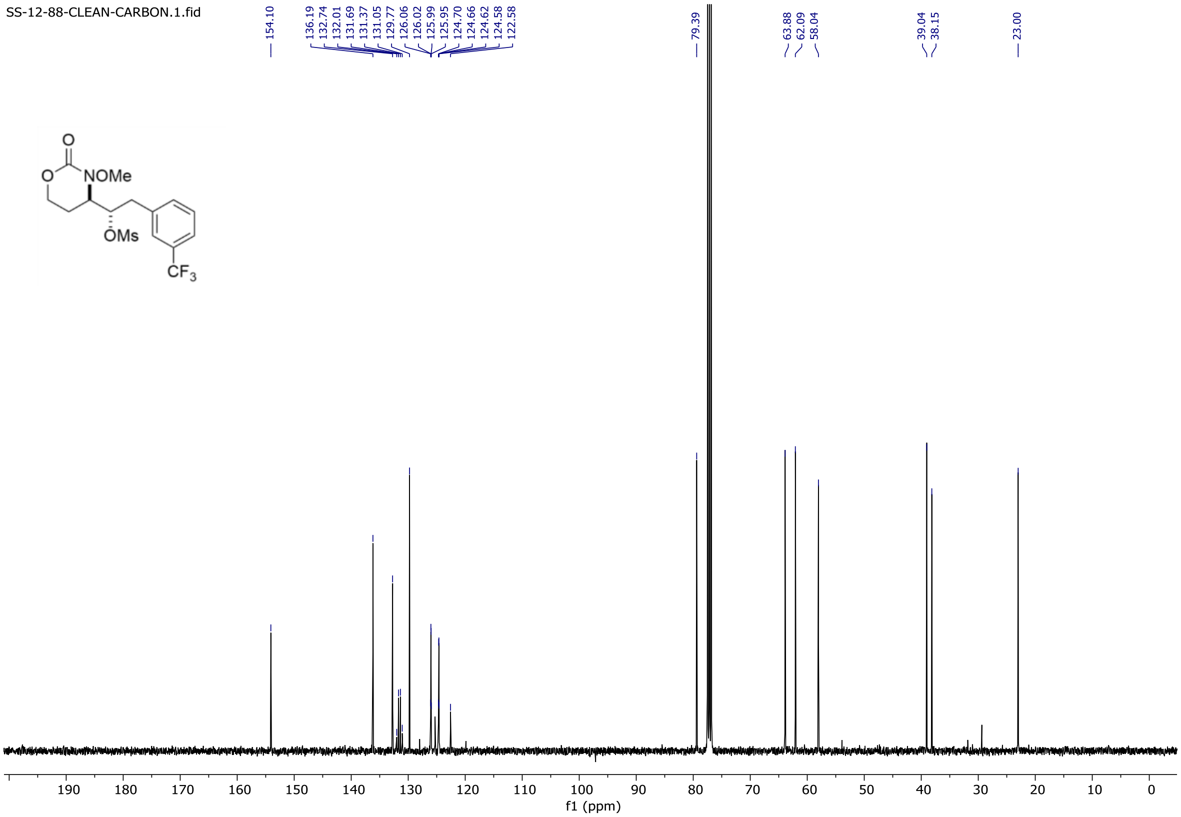

Compound 47 (CDCl₃, ¹H NMR: 400 MHz, ¹³C{¹H} NMR: 101 MHz)

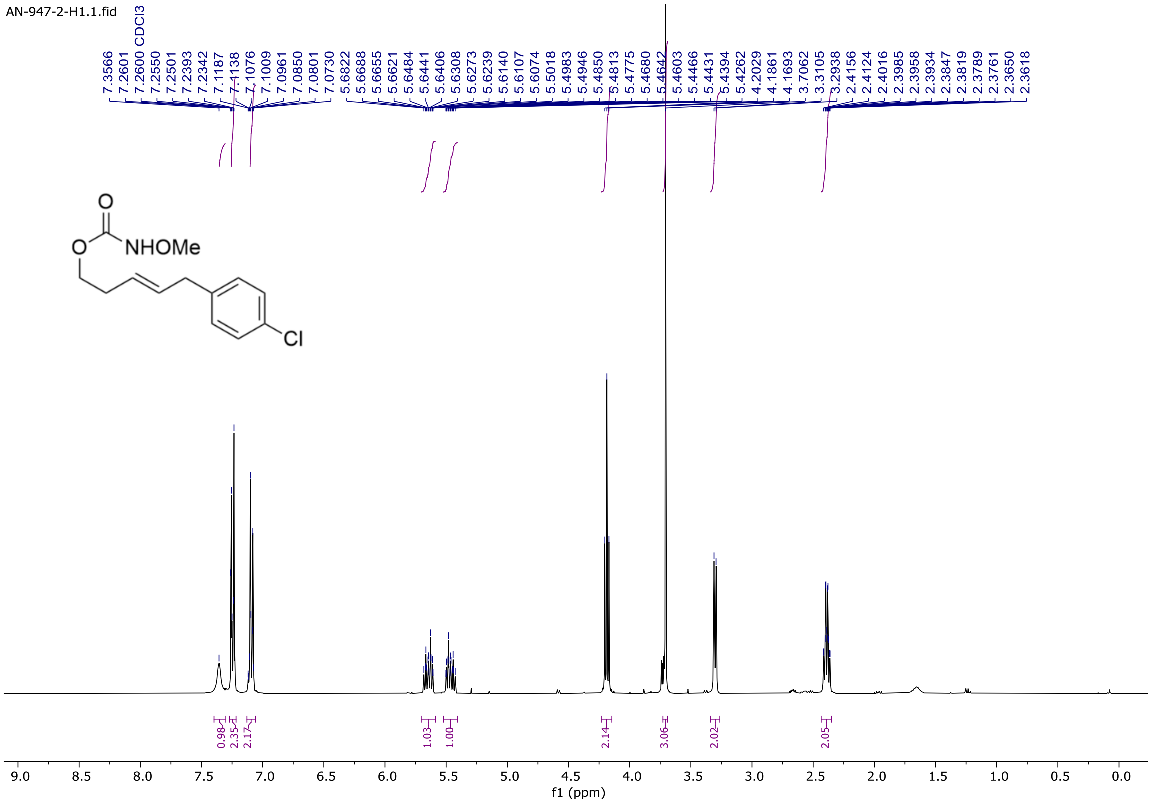

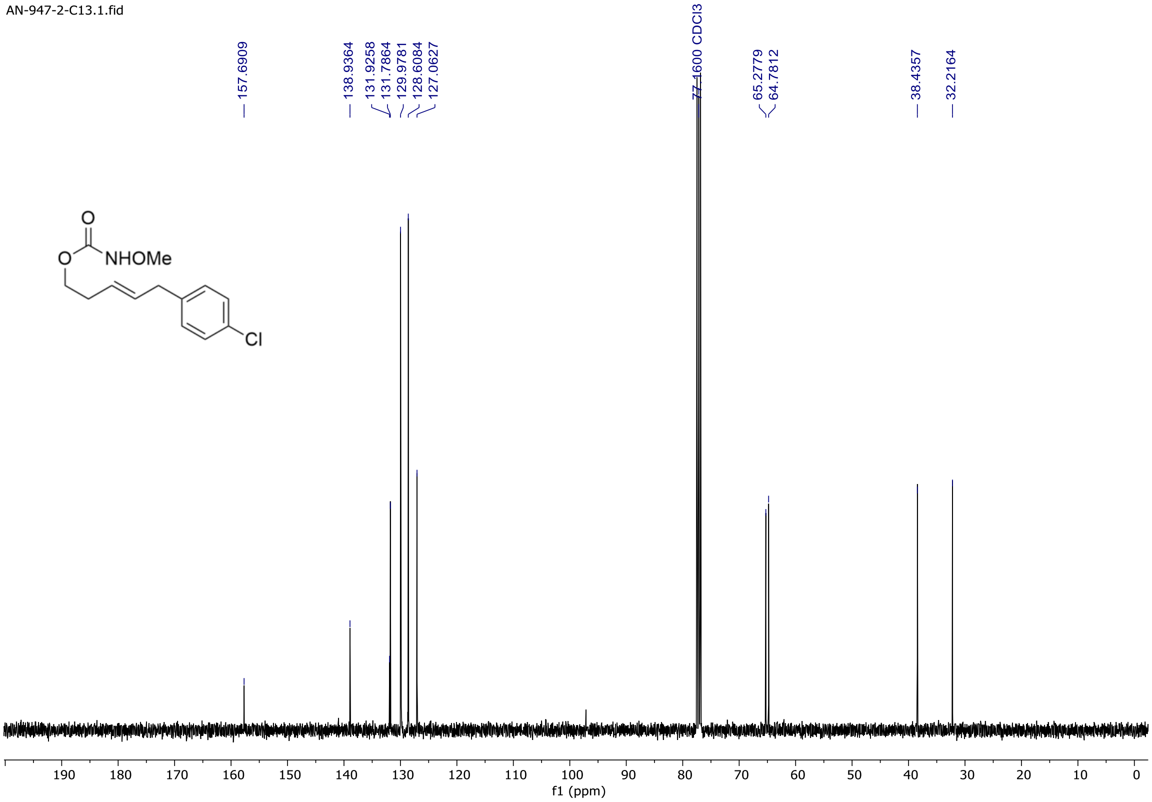

Compound 48 ($CDCl_3$, ^{1}H NMR: 600 MHz, ^{13}C{^{1}H} NMR: 101 MHz)

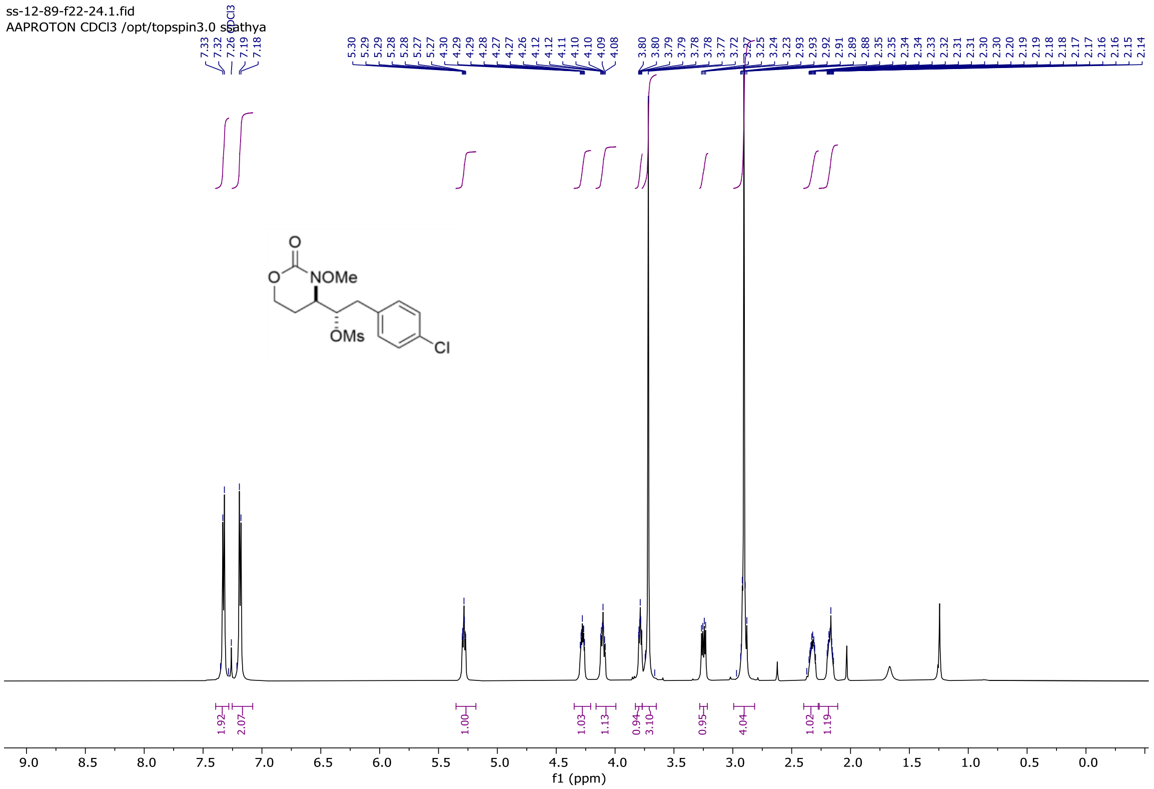

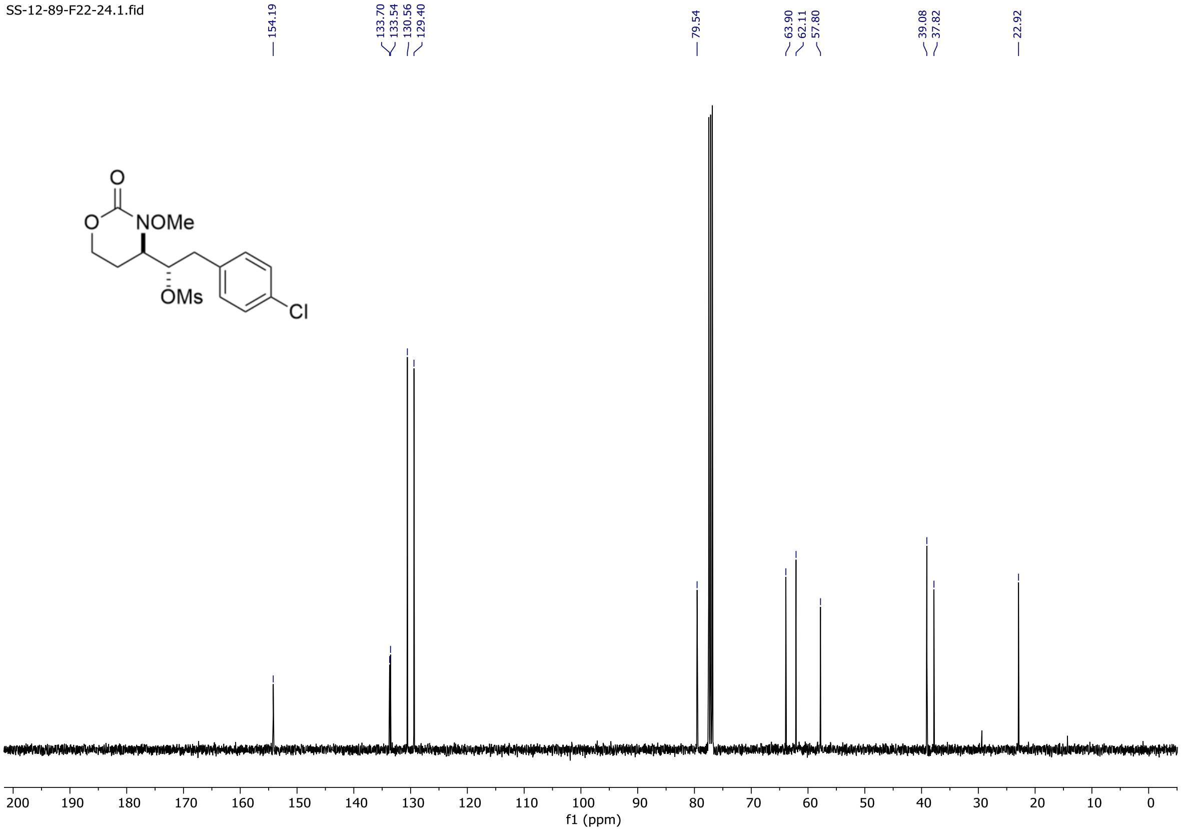

Compound 49 ($CDCl_3$, ^1H NMR: 400 MHz, $^{13}C\{^1H\}$ NMR: 101 MHz)

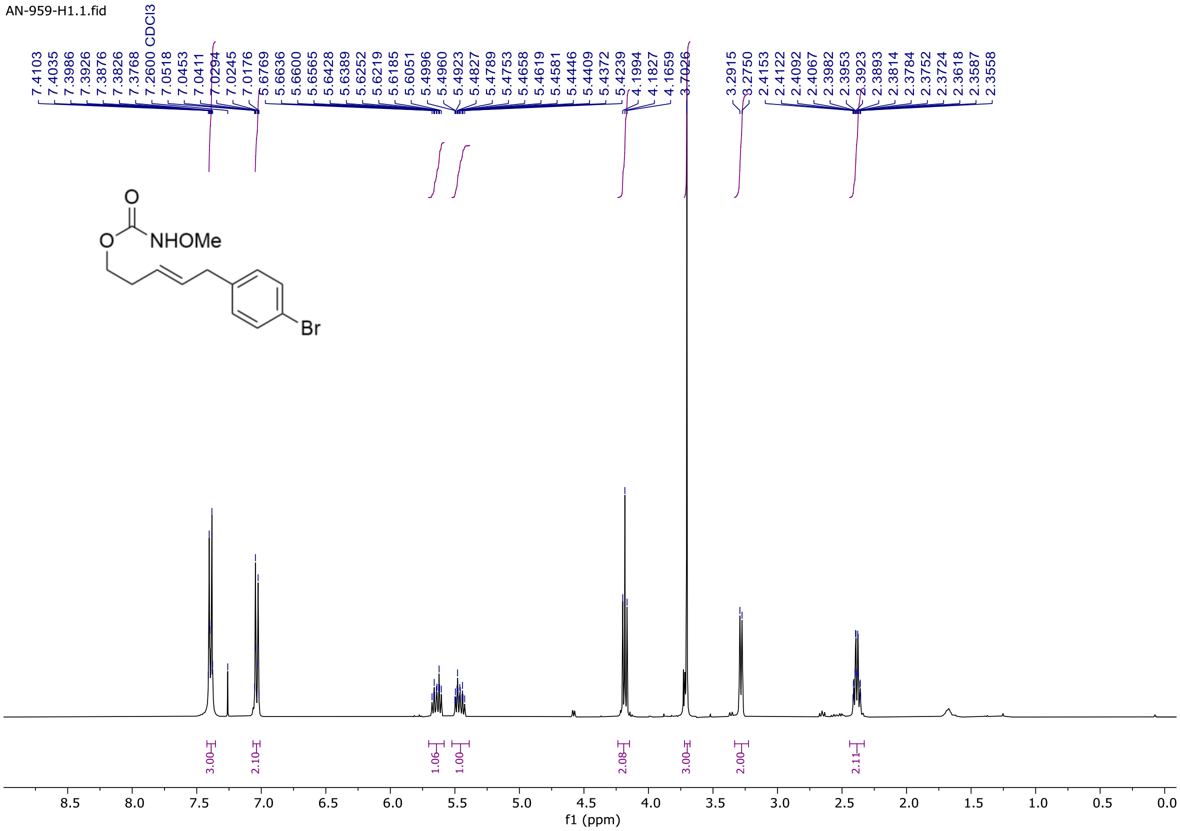

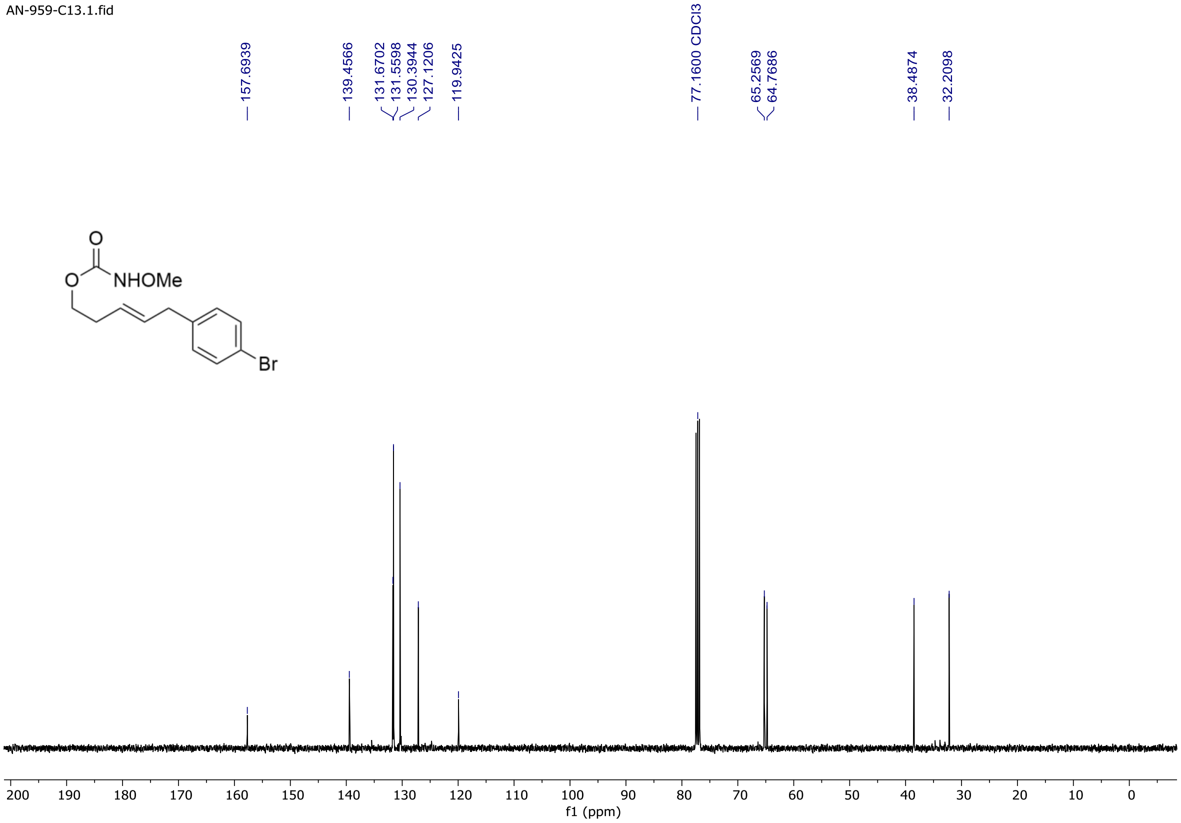

Compound 50 ($CDCl_3$, ^1H NMR: 400 MHz, $^{13}C\{^1H\}$ NMR: 101 MHz)

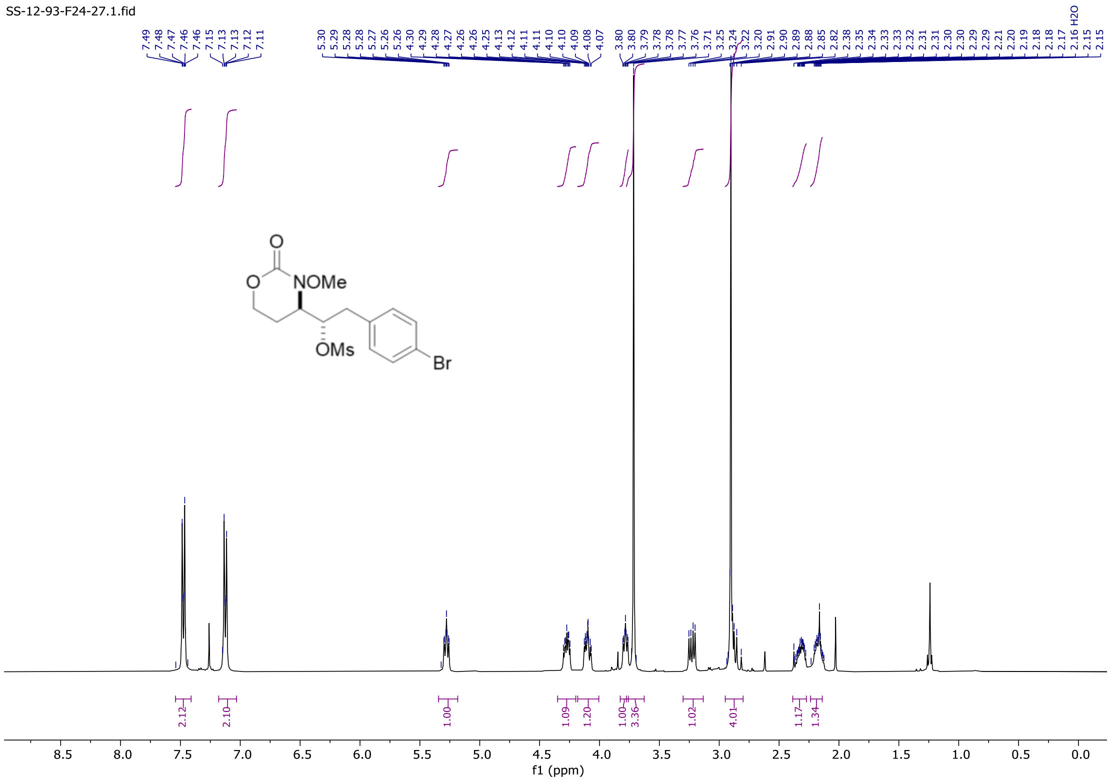

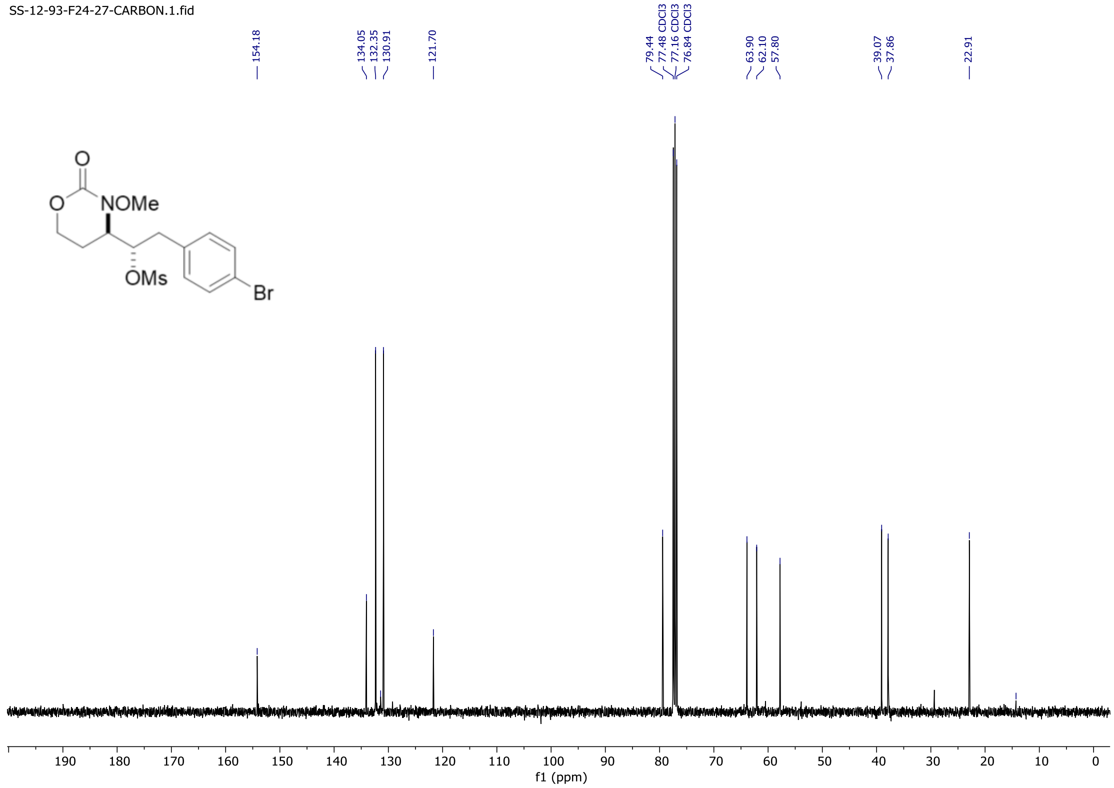

Compound 51 ($CDCl_3$, ^1H NMR: 400 MHz, $^{13}C\{^1H\}$ NMR: 101 MHz)

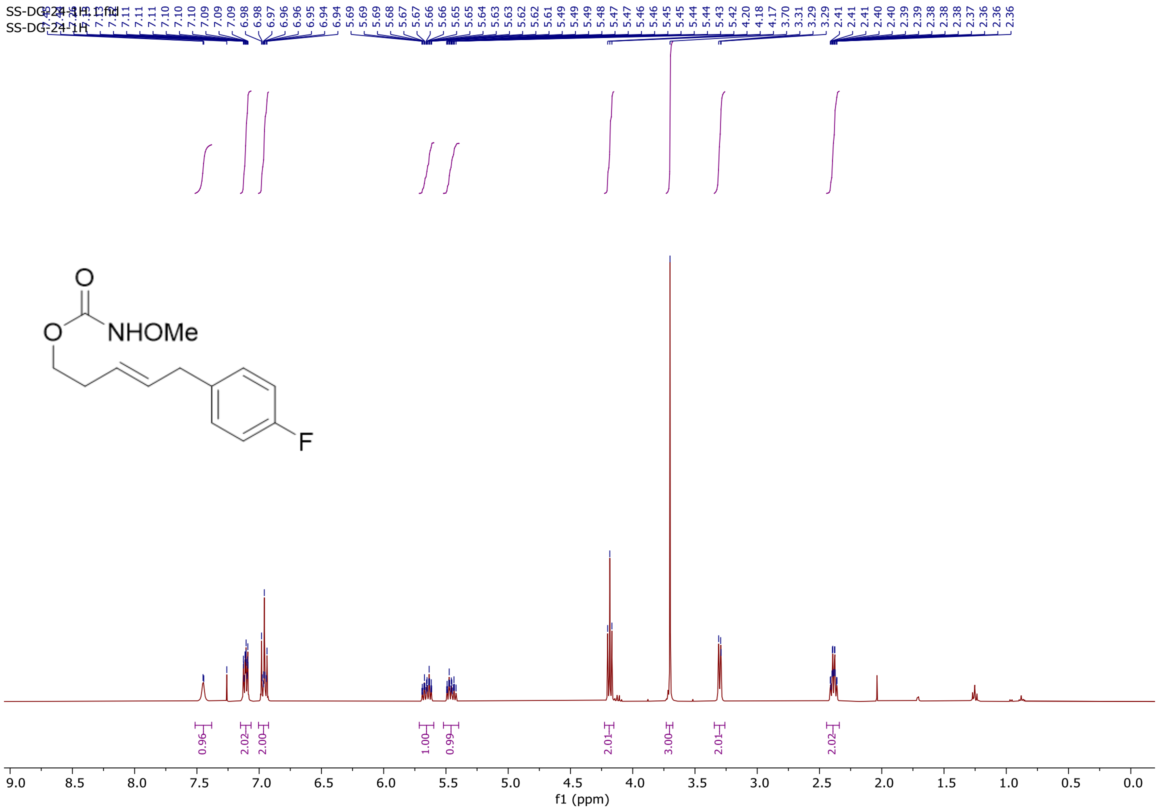

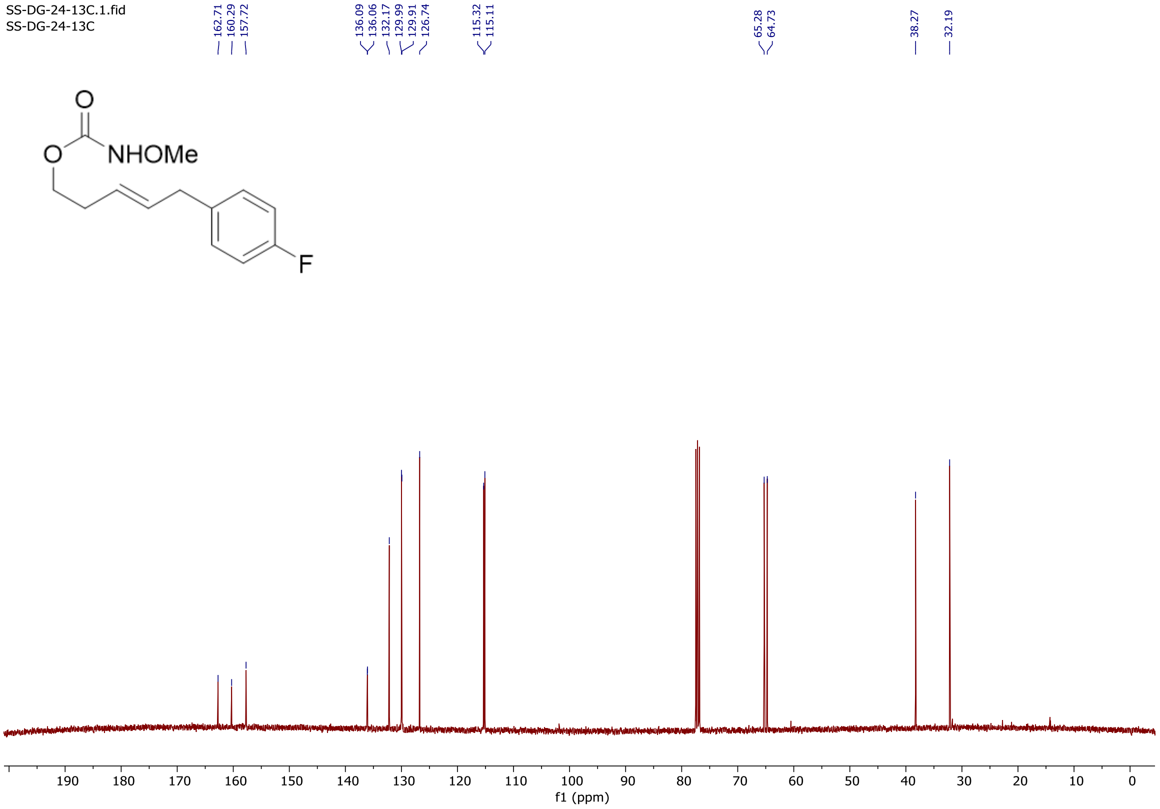

Compound 52 ($CDCl_3$, ^1H NMR: 400 MHz, $^{13}C\{^1H\}$ NMR: 101 MHz)

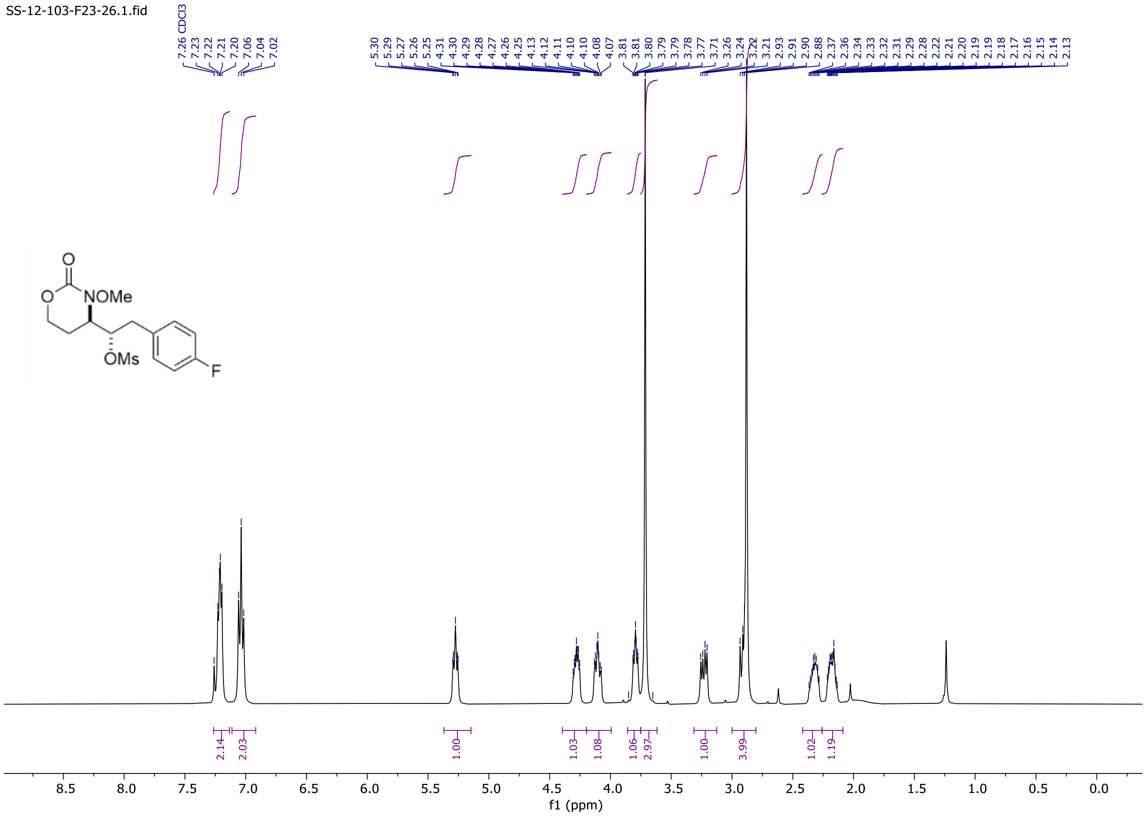

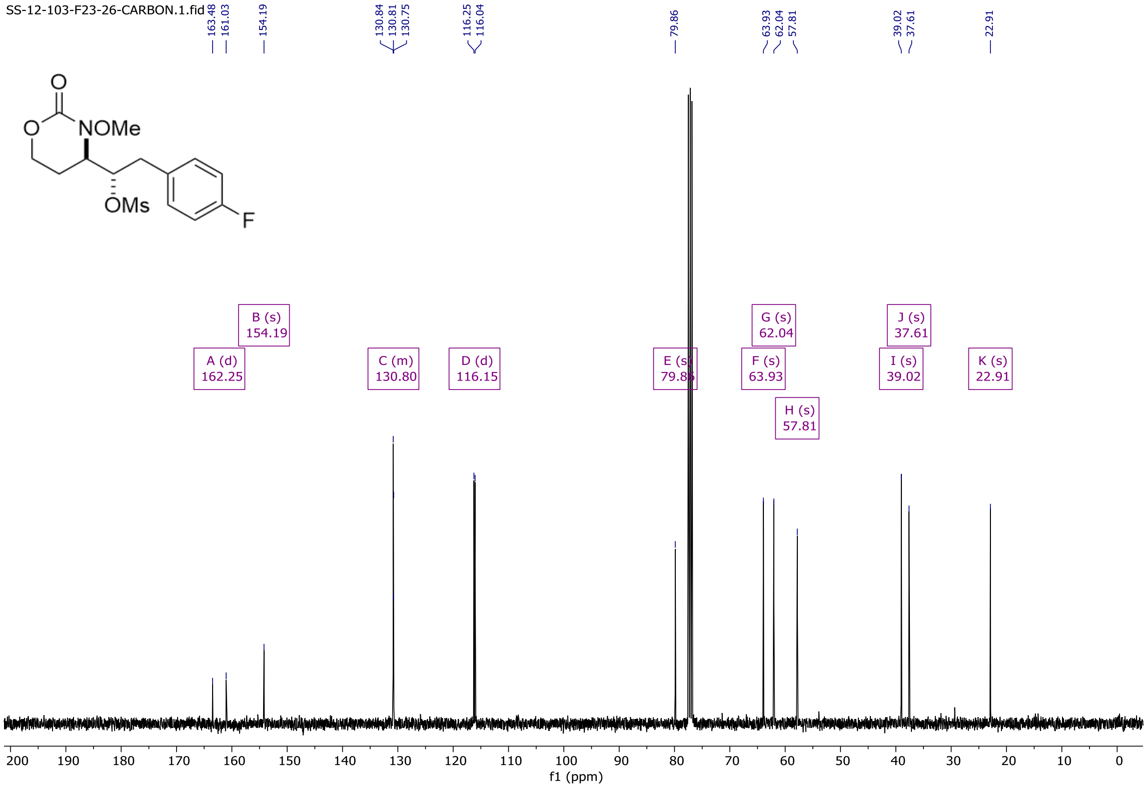

Compound 54 (CDCl₃, ^{1}H NMR: 600 MHz, ^{13}C{^{1}H} NMR: 101 MHz)

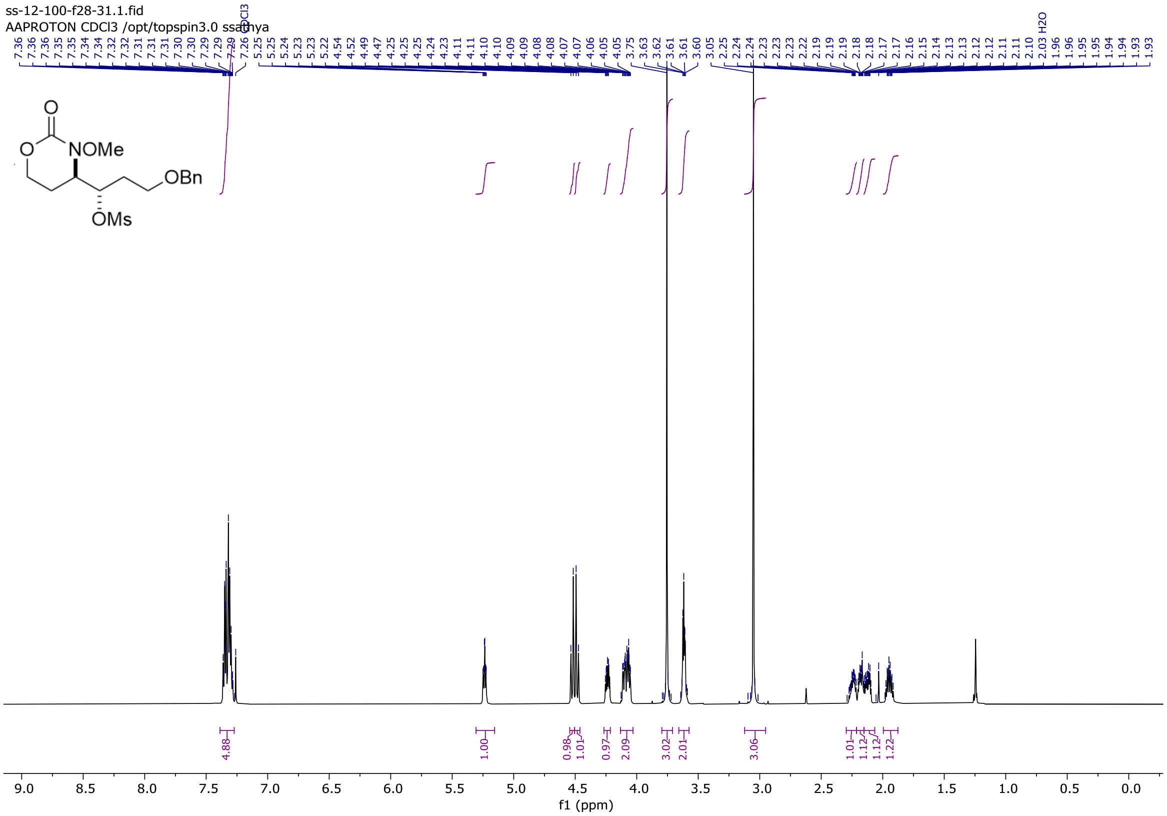

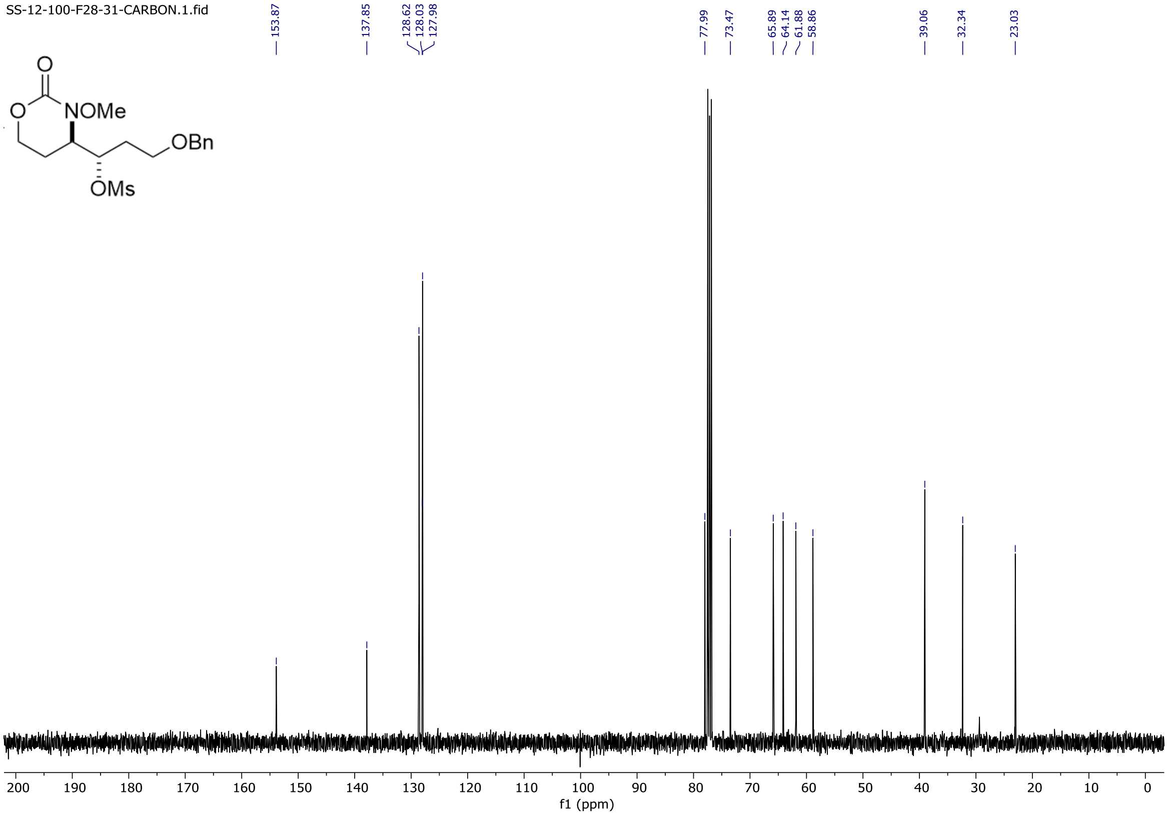

Compound 55 ($CDCl_3$, ^1H NMR: 400 MHz, $^{13}C\{^1H\}$ NMR: 101 MHz)

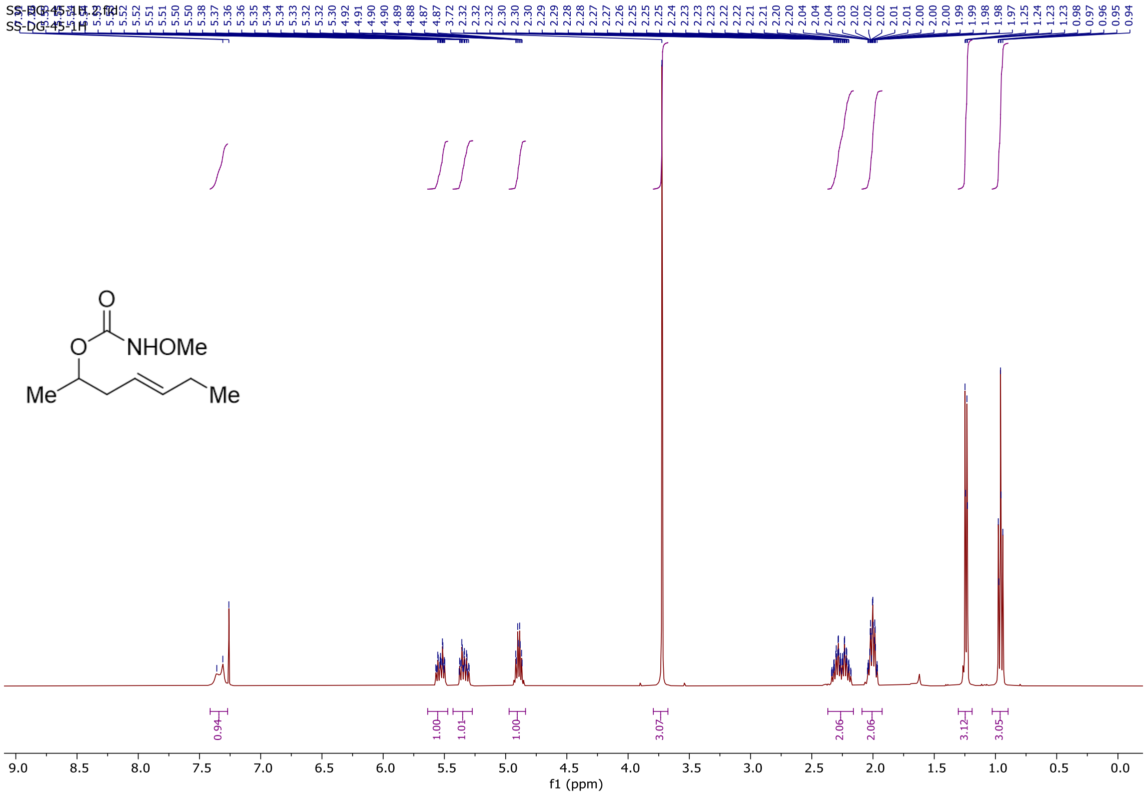

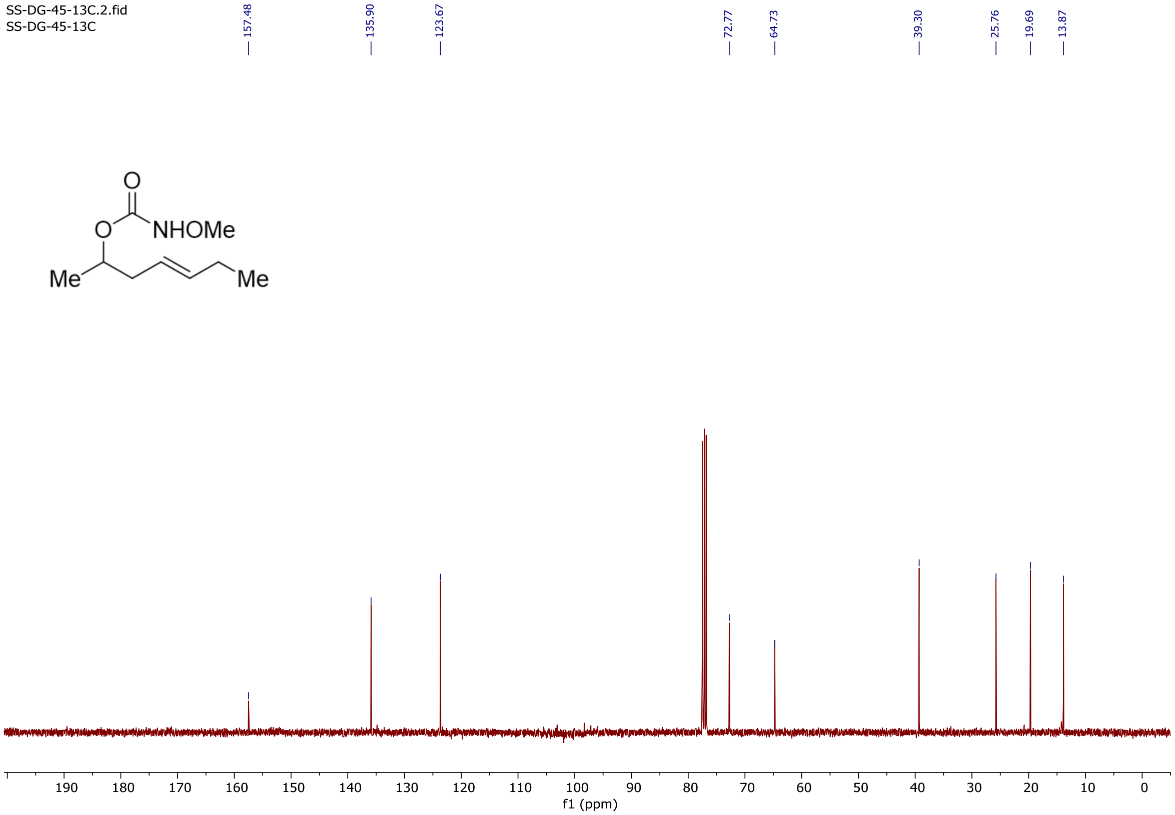

Compound 56 (CDCl₃, ^{1}H NMR: 400 MHz, ^{13}C{^{1}H} NMR: 101 MHz)

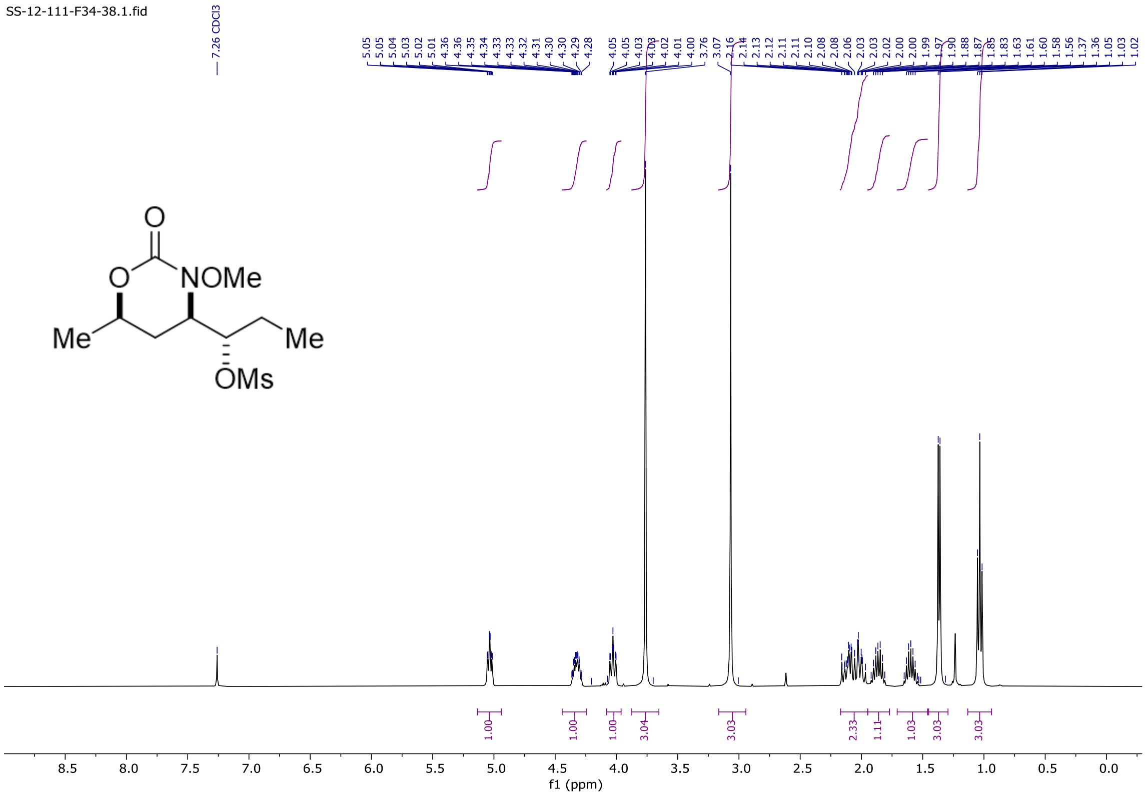

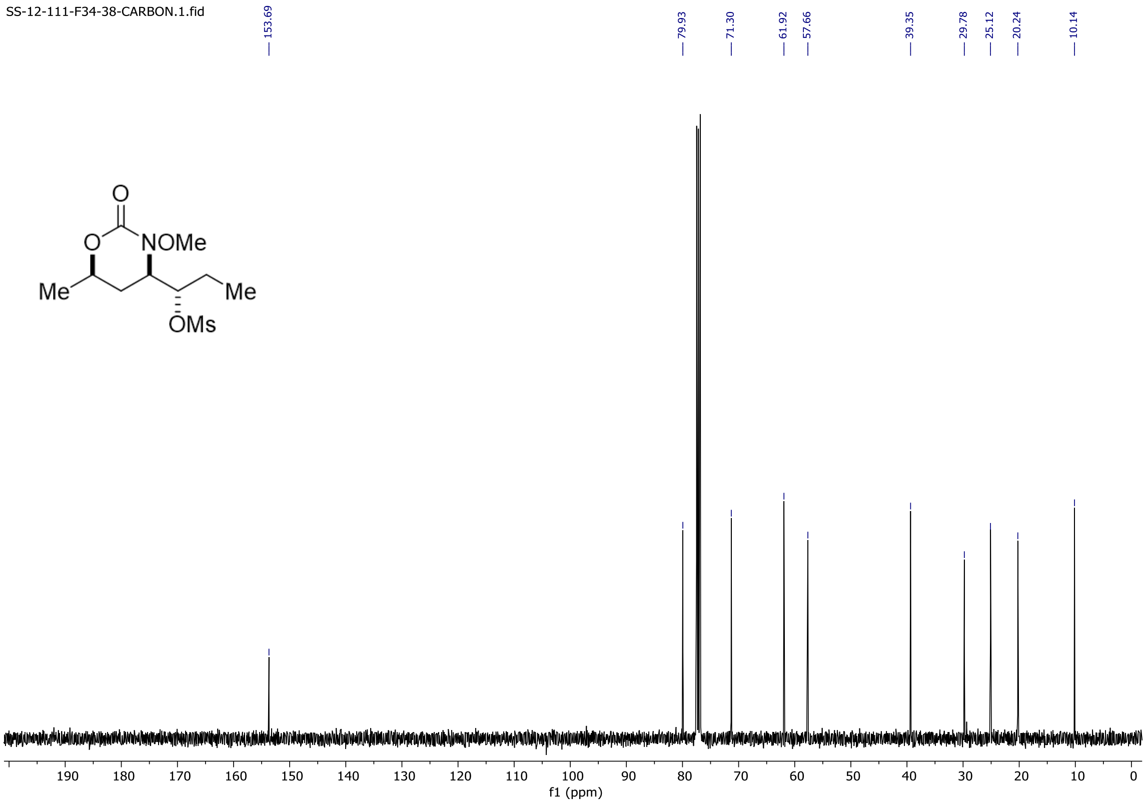

Compound 59 ($CDCl_3$, ^1H NMR: 600 MHz, $^{13}C\{^1H\}$ NMR: 101 MHz)

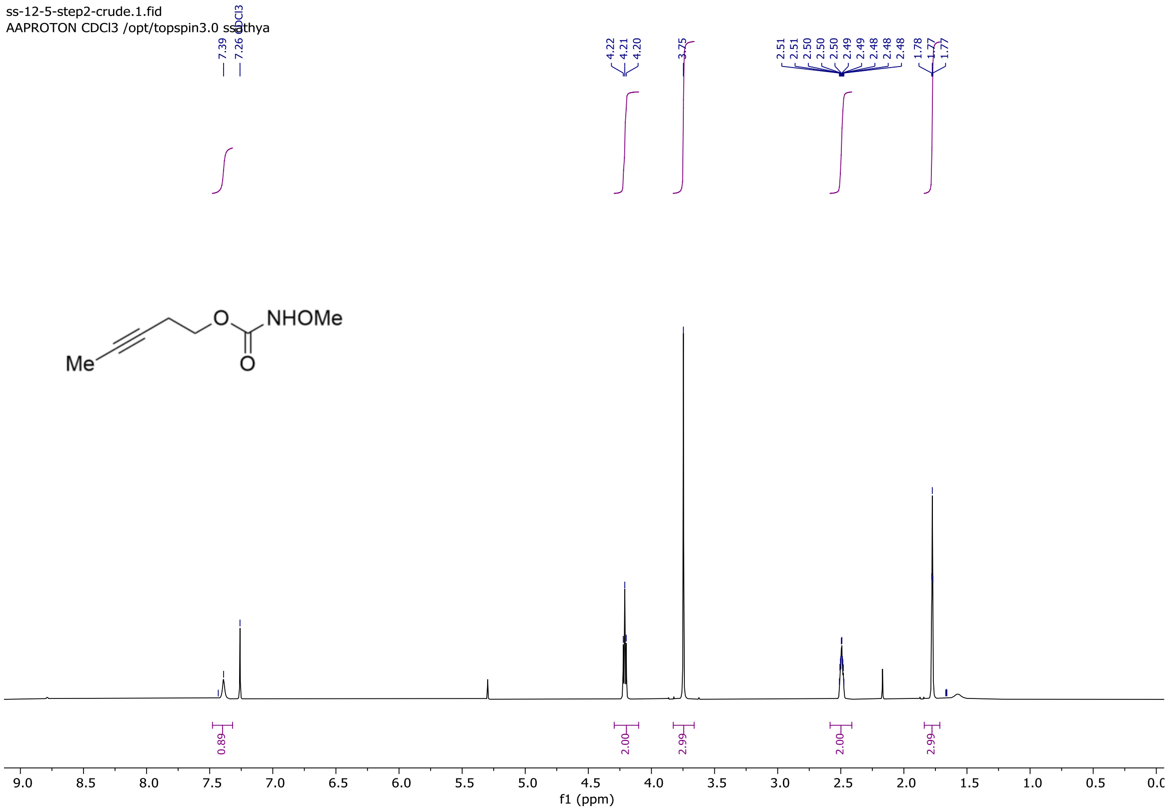

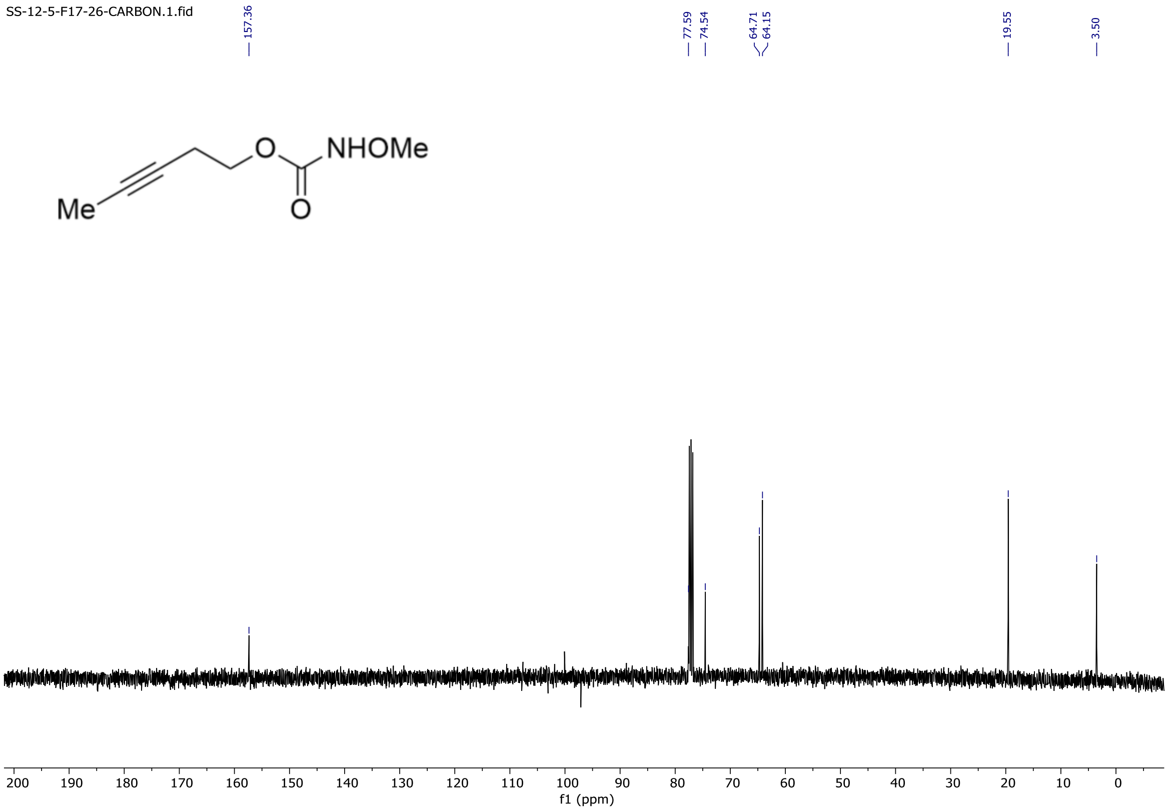

Compound 61 ($CDCl_3$, ^1H NMR: 400 MHz, $^{13}C\{^1H\}$ NMR: 101 MHz)

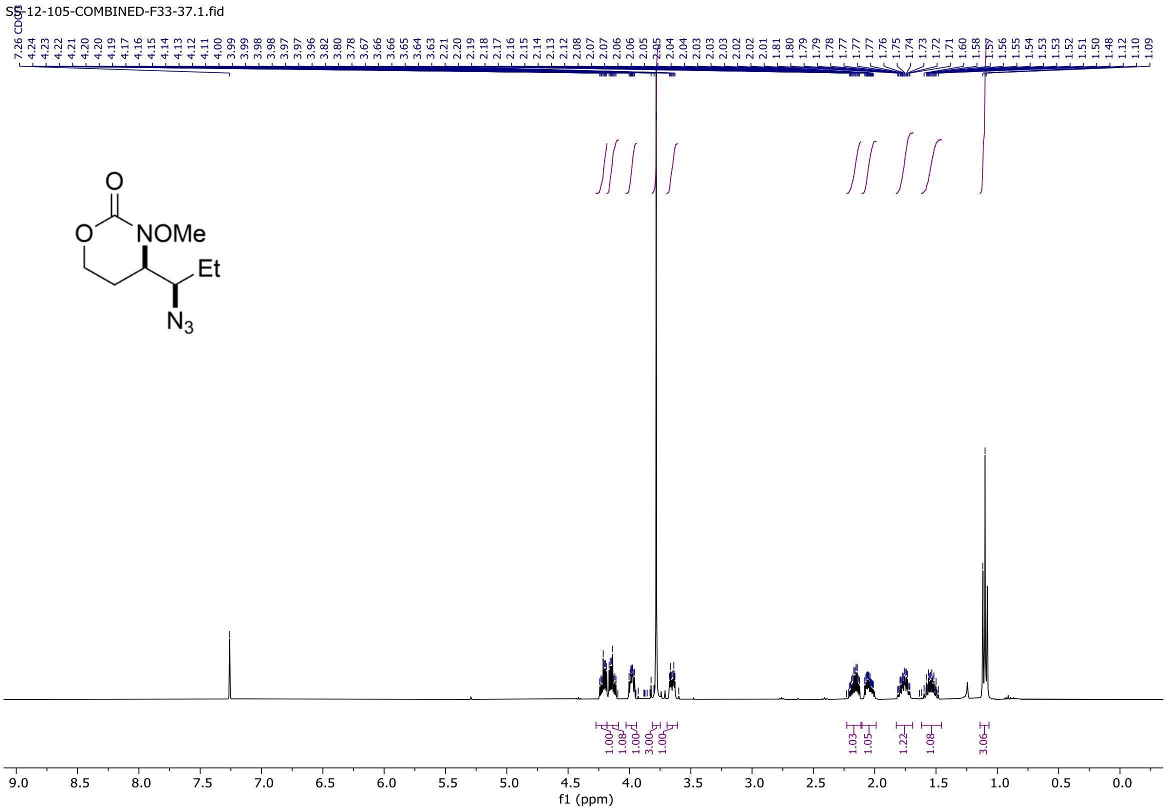

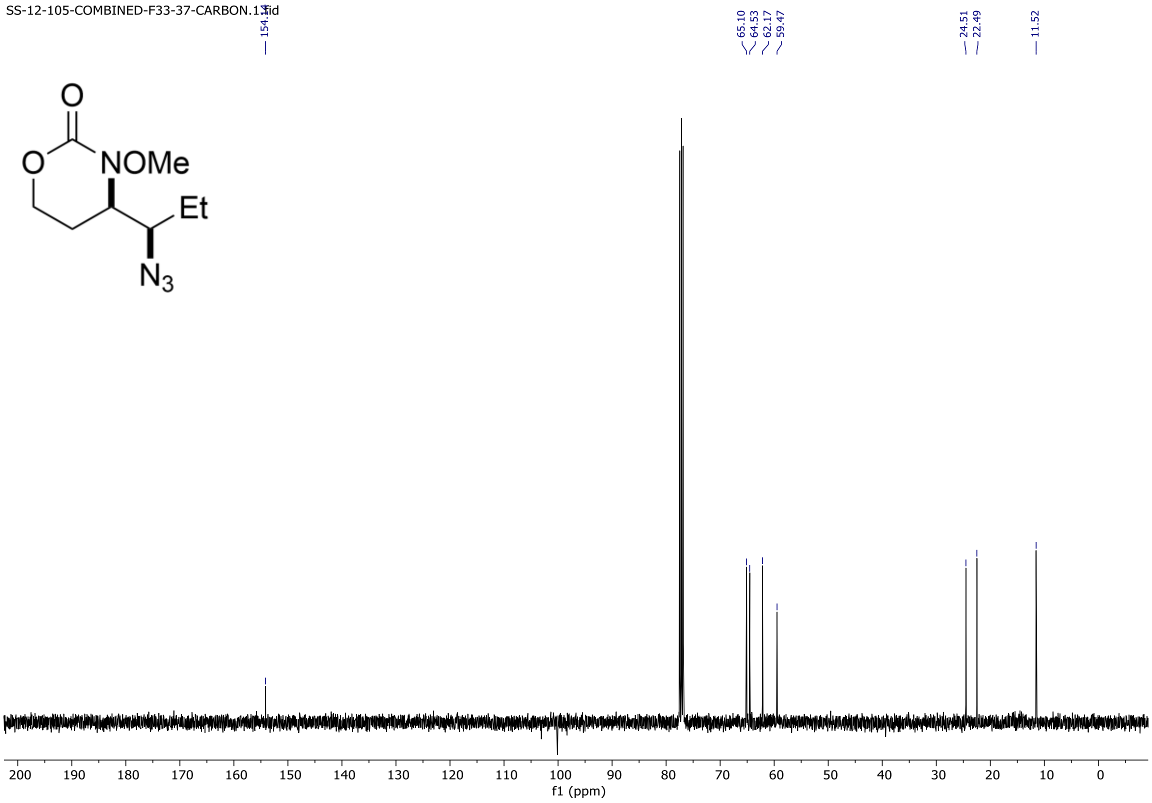

Compound 62 ($CDCl_3$, ^1H NMR: 500 MHz, $^{13}C\{^1H\}$ NMR: 126 MHz, 55 °C)

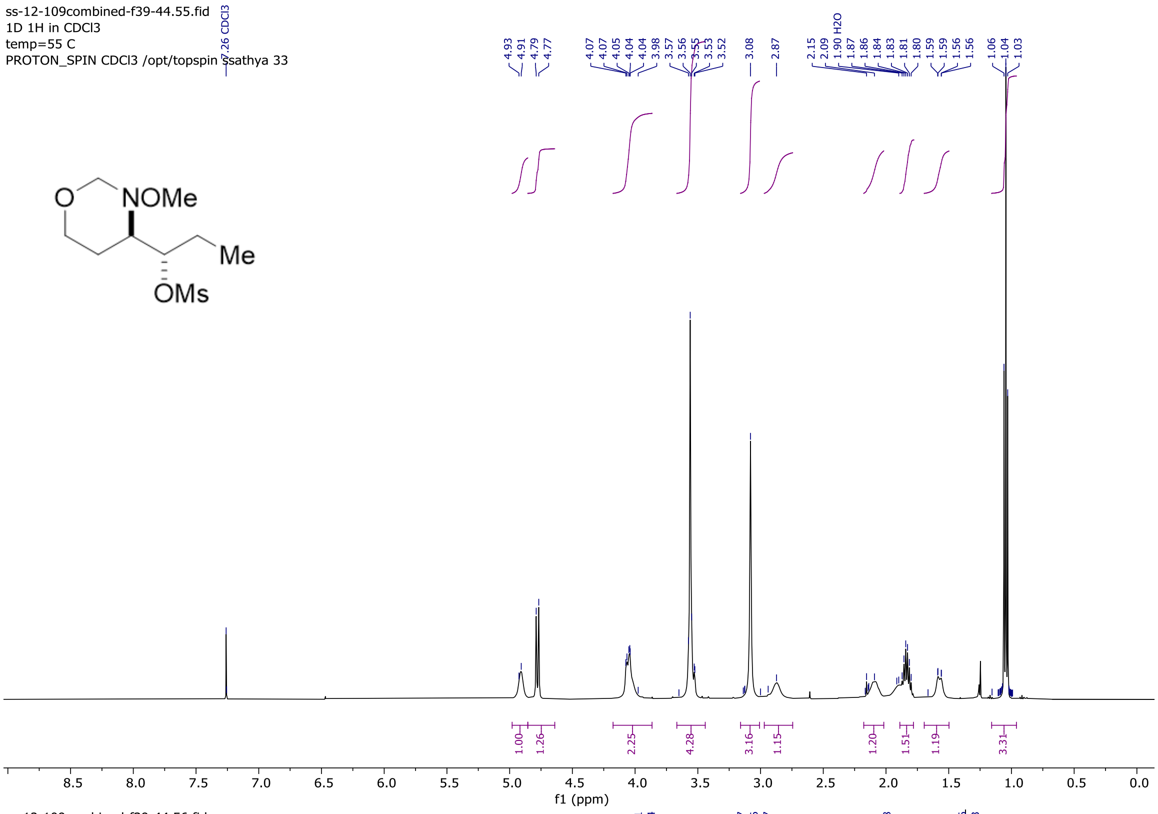

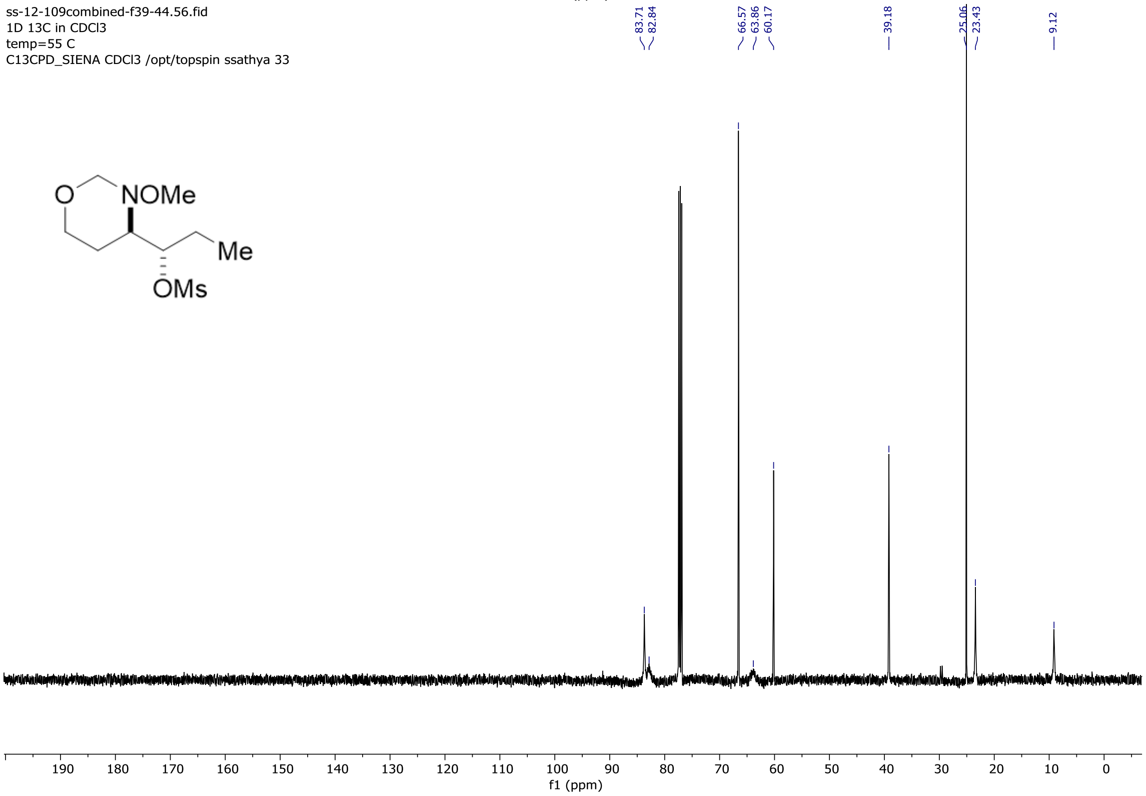

Supplement: File 1 — Additional experimental details including reaction procedures, X-ray crystallographic data, and NMR spectra of synthesized compounds. [file Beilstein_J_Org_Chem-21-947-s001.pdf]
